# Disease-specific biomarker value of C-reactive protein and its composite indices in autoinflammatory diseases: an integrated study of multi-modal omics and population cohorts

**DOI:** 10.3389/fimmu.2026.1874052

**Published:** 2026-08-13

**Authors:** Qianyue Yang, Yulu Zhang, Xiaomin Li, Shuning Sun, Liuting Zeng, Yanfang Luo, Xin Wang, Lingyun Sun

**Affiliations:** 1 Department of Rheumatology and Immunology, Nanjing Drum Tower Hospital Clinical College of Nanjing Medical University, Nanjing, China; 2 Department of Rheumatology and Immunology, Nanjing Drum Tower Hospital, Chinese Academy of Medical Sciences & Peking Union Medical College, Nanjing, China; 3 Department of Nephrology, The Central Hospital of Shaoyang, Shaoyang, Hunan, China; 4 Department of Rheumatology and Immunology, Nanjing Drum Tower Hospital, Nanjing, China

**Keywords:** autoinflammatory diseases, composite inflammatory index, C-reactive protein, disease-specific biomarker, hepatocyte origin, systemic inflammation

## Abstract

**Background:**

Autoinflammatory diseases (AIDs) are a heterogeneous group of innate immune-driven disorders whose prevalence is rising globally, yet the lack of disease-specific biomarkers continues to hamper accurate disease activity assessment, phenotype stratification, and risk evaluation. C-reactive protein (CRP) is one of the most widely used markers of systemic inflammation, yet its disease-specific expression, clinical correlations, network connectivity, and cellular origin across both adult and pediatric AIDs have not been systematically examined.

**Methods:**

We established a single-center retrospective cohort including 11 adult and pediatric AIDs and age matched healthy controls. Baseline CRP measurements were obtained before anti-inflammatory or immunosuppressive therapy. We performed multivariate regression and subgroup analyses to examine the associations of CRP and its composite indices, the CRP to lymphocyte ratio (CLR) and CRP to albumin ratio (CAR), with different AIDs. We integrated a public serum proteomic dataset to construct CRP centered protein interaction networks in macrophage activation syndrome. We also analyzed single cell CITE seq and spatial transcriptomic data from healthy human liver to determine the cellular origin of CRP.

**Results:**

Serum CRP levels showed a disease specific distribution across AIDs, with the highest levels in hyperinflammatory conditions such as AOSD and MAS. CRP correlated with fever, rash, and arthralgia in AOSD, but showed no association with mucosal or articular phenotypes in other diseases. Both CLR and CAR showed stronger associations with disease risk than CRP alone in regression models. Proteomic network analysis positioned CRP as a prominently connected node in the MAS inflammatory interactome. Single cell and spatial transcriptomic data confirmed hepatocytes as the exclusive source of CRP transcripts in healthy human liver.

**Conclusions:**

These findings indicate a positive association of CRP and its composite indices with AIDs, suggesting their potential as disease-specific biomarkers. Yet, given the single-center retrospective design and lack of external validation, further multi-center prospective studies are required before clinical implementation.

## 1 Introduction

Autoinflammatory diseases (AIDs) are a heterogeneous group of disorders driven by innate immune dysregulation. Unlike classical autoimmune diseases where adaptive immunity predominates (1), AIDs may present with recurrent fever, systemic inflammation, and multi-organ involvement including joints, skin, mucous membranes, gastrointestinal tract, lungs, and hematopoietic system (2–4). As diagnostic criteria and clinical awareness improve, the reported incidence of AIDs has steadily increased, establishing them as a notable burden in rheumatology (5, 6). However, substantial phenotypic heterogeneity and variable disease courses make accurate disease activity assessment, early complication identification, and patient stratification ongoing challenges that affect treatment optimization and long-term prognosis (7–9).

Recent years have seen biomarker discovery and translation take on a key role in addressing these challenges in AIDs (10, 11). Among all inflammatory molecules, C-reactive protein (CRP) is a classic and widely used serological marker of the acute-phase response. With easy detection, low cost, and rapid turnover, it has become routine for screening infections, evaluating inflammation, and monitoring treatment (12, 13). As a pentraxin family pattern-recognition molecule (14, 15), CRP binds pathogen phosphocholine, activates complement, and regulates innate immune cell activation (16). Nevertheless, its biological and clinical roles have largely been viewed through the lens of a non-specific inflammatory marker (17, 18).

Current research on CRP in AIDs has several gaps. First, many existing studies focus on infections or single inflammatory conditions; a systematic expression profile across the AID spectrum is lacking (19, 20). Second, bulk-level analyses cannot identify the tissue- or cell-specific origins of CRP or its transcriptional regulatory network at single-cell resolution. These limitations may hinder CRP’s transition from a “non-specific inflammatory indicator” to a precise biomarker for disease classification, activity assessment, or complication warning in AIDs. Thus, the disease-specific expression heterogeneity and pathological mechanisms linking CRP to organ complications across different AIDs require further clarification (21–23).

The integration of real-world cohorts with high-throughput sequencing is now feasible through multi-omics and big data approaches, offering a systematic strategy for investigating disease-specific inflammatory features (24, 25). To address previous limitations, we analyzed large retrospective cohorts from Nanjing Drum Tower Hospital and explored public proteomics and single-cell RNA sequencing datasets. We assessed the relationship between CRP, its composite indices, and multiple AIDs, and applied multi-omics analyses to characterize underlying cellular and molecular features. Although further validation is needed, these findings may provide a theoretical and clinical basis for developing CRP-based precision stratification and early warning systems for AIDs complications.

## 2 Materials and methods

### 2.1 Single-center retrospective clinical study cohort

The single-center retrospective study established 13 independent subcohorts, including 8 adult autoinflammatory diseases, 2 pediatric autoinflammatory diseases, and 2 healthy control groups: adult-onset Still’s disease (AOSD), macrophage activation syndrome (MAS), Behçet’s disease (BD), spondyloarthritis (SpA), inflammatory bowel disease (IBD), gout, relapsing polychondritis (RP), sarcoidosis (SAR), systemic juvenile idiopathic arthritis (SJIA), Kawasaki disease (KD), Sweet syndrome (SS), healthy adult controls (HC-Adult), and healthy child controls (HC-Child). Adult disease subcohorts were restricted to patients aged ≥18 years, while pediatric subcohorts (SJIA, KD, SS) and HC-Child were limited to individuals aged <18 years. Overlap between individual subcohorts, such as IBD and SpA, AOSD and MAS, or SJIA and MAS, may exist due to the pathological nature of these conditions. In the present cohort, we further examined the extent of overlap between specific disease pairs. For IBD and SpA, only one patient met criteria for both conditions due to our matched sampling strategy that prioritized CRP data completeness. Similarly, for SJIA and MAS, only one overlapping case was identified. Therefore, no sensitivity analyses were performed for these two pairs. For AOSD and MAS, among 31 patients diagnosed with AOSD, 21 (67.7%) also met MAS diagnostic criteria. This high overlap reflects the recognized clinical entity of MAS secondary to active AOSD. Due to the limited number of non-overlapping AOSD cases (n = 10), formal sensitivity analyses excluding overlapping patients were not statistically feasible. All subjects were inpatients or outpatients at Nanjing Drum Tower Hospital between January 2021 and January 2023, or healthy individuals undergoing routine physical examinations at the hospital’s Physical Examination Center or Department of Child Healthcare during the same period.

#### 2.1.1 Unified exclusion criteria

Subjects were excluded if they met any of the following conditions: ① Complicated with other autoimmune diseases, severe infectious diseases, malignant tumors, or severe liver and kidney dysfunction; ② Received targeted drug therapy (including glucocorticoids, immunosuppressants, biological agents), induction therapy, intravenous immunoglobulin (IVIG), or any other interventions that may alter serum CRP levels prior to sample collection; ③ Incomplete baseline clinical data or missing baseline CRP test results; ④ Pregnant or lactating women. Healthy controls were additionally excluded if they had a history of acute or chronic infection, surgery, or trauma within the past 3 months, vaccination within the past 1 month, or any chronic inflammatory or systemic disease.

#### 2.1.2 Adult-onset still’s disease subcohort

A subcohort of adult-onset Still’s disease (AOSD) was established, including patients diagnosed with AOSD at Nanjing Drum Tower Hospital between January 2021 and January 2023. Inclusion Criteria: ① Age ≥ 18 years; ② Met the Yamaguchi classification criteria for adult-onset Still’s disease or the 2020 ACR/EULAR classification criteria; ③ Complete baseline clinical data, including CRP test results at enrollment. All CRP samples were collected at baseline, prior to the initiation of any targeted drug therapy or induction therapy, to eliminate potential interference from therapeutic interventions on CRP levels. Patients who met the unified exclusion criteria were excluded.

#### 2.1.3 Macrophage activation syndrome subcohort

A subcohort of macrophage activation syndrome (MAS) was established, including patients diagnosed with MAS at Nanjing Drum Tower Hospital between January 2021 and January 2023. Inclusion Criteria: ① Age ≥ 18 years; ② Met the internationally recognized diagnostic criteria for macrophage activation syndrome; ③ Complete baseline clinical data, including CRP test results at enrollment. All CRP samples were collected at baseline, prior to the initiation of any targeted drug therapy or induction therapy. Patients who met the unified exclusion criteria were excluded.

#### 2.1.4 Behçet’s disease subcohort

A subcohort of Behçet’s disease (BD) was established, including patients diagnosed with BD at Nanjing Drum Tower Hospital between January 2021 and January 2023. Inclusion Criteria: ① Age ≥ 18 years; ② Met the 2014 ICBD Behçet’s disease classification criteria or the 2018 EULAR/ACR Behçet’s disease classification criteria; ③ Complete baseline clinical data, including CRP test results at enrollment. All CRP samples were collected at baseline, prior to the initiation of any targeted drug therapy or induction therapy. Patients who met the unified exclusion criteria were excluded.

#### 2.1.5 Spondyloarthritis subcohort

A subcohort of spondyloarthritis (SpA) was established, including patients diagnosed with SpA at Nanjing Drum Tower Hospital between January 2021 and January 2023. Inclusion Criteria: ① Age ≥ 18 years; ② Met the 2009 ASAS spondyloarthritis classification criteria; ③ Complete baseline clinical data, including CRP test results at enrollment. All CRP samples were collected at baseline, prior to the initiation of any targeted drug therapy or induction therapy. Patients who met the unified exclusion criteria were excluded.

#### 2.1.6 Inflammatory bowel disease subcohort

A subcohort of inflammatory bowel disease (IBD) was established, including patients diagnosed with IBD at Nanjing Drum Tower Hospital between January 2021 and January 2023. Inclusion Criteria: ① Age ≥ 18 years; ② Met the corresponding internationally recognized diagnostic criteria for inflammatory bowel disease; ③ Complete baseline clinical data, including CRP test results at enrollment. All CRP samples were collected at baseline, prior to the initiation of any targeted drug therapy or induction therapy. Patients who met the unified exclusion criteria were excluded.

#### 2.1.7 Crystal-related arthritis (gout) subcohort

A subcohort of crystal-related arthritis (gout) was established, including patients diagnosed with gout at Nanjing Drum Tower Hospital between January 2021 and January 2023. Inclusion Criteria: ① Age ≥ 18 years; ② Met the 2015 ACR/EULAR gout classification criteria; ③ Complete baseline clinical data, including CRP test results at enrollment. All CRP samples were collected at baseline, prior to the initiation of any targeted drug therapy or induction therapy. Patients who met the unified exclusion criteria were excluded.

#### 2.1.8 Relapsing polychondritis subcohort

A subcohort of relapsing polychondritis (RP) was established, including patients diagnosed with relapsing polychondritis at Nanjing Drum Tower Hospital between January 2021 and January 2023. Inclusion Criteria: ① Age ≥ 18 years; ② Met the Michet diagnostic criteria or the 2019 ICRPC diagnostic criteria for relapsing polychondritis; ③ Complete baseline clinical data, including CRP test results at enrollment. All CRP samples were collected at baseline, prior to the initiation of any targeted drug therapy or induction therapy. Patients who met the unified exclusion criteria were excluded.

#### 2.1.9 Sarcoidosis subcohort

A subcohort of sarcoidosis (SAR) was established, including patients diagnosed with sarcoidosis at Nanjing Drum Tower Hospital between January 2021 and January 2023. Inclusion Criteria: ① Age ≥ 18 years; ② Met the 2016 ATS/ERS/WASOG/ALAT sarcoidosis classification criteria; ③ Complete baseline clinical data, including CRP test results at enrollment. All CRP samples were collected at baseline, prior to the initiation of any targeted drug therapy or induction therapy. Patients who met the unified exclusion criteria were excluded.

#### 2.1.10 Sweet syndrome subcohort

A subcohort of Sweet syndrome (SS) was established, including patients diagnosed with SS at Nanjing Drum Tower Hospital between January 2021 and January 2023. Inclusion Criteria: ① Age ≥ 18 years; ② Met the 2021 EULAR diagnostic criteria for Sweet syndrome; ③ Complete baseline clinical data, including CRP test results at enrollment. All CRP samples were collected at baseline, prior to the initiation of glucocorticoids or any other immunosuppressive therapy. Patients who met the unified exclusion criteria were excluded.

#### 2.1.11 Systemic juvenile idiopathic arthritis subcohort

A subcohort of systemic juvenile idiopathic arthritis (SJIA) was established, including patients diagnosed with SJIA at Nanjing Drum Tower Hospital between January 2021 and January 2023. Inclusion Criteria: ① Age < 18 years; ② Met the 2019 ILAR classification criteria for systemic juvenile idiopathic arthritis; ③ Complete baseline clinical data, including CRP test results at enrollment. All CRP samples were collected at baseline, prior to the initiation of any targeted drug therapy, induction therapy, or IVIG. Patients who met the unified exclusion criteria were excluded.

#### 2.1.12 Kawasaki disease subcohort

A subcohort of Kawasaki disease (KD) was established, including patients diagnosed with KD at Nanjing Drum Tower Hospital between January 2021 and January 2023. Inclusion Criteria: ① Age < 18 years; ② Met the 2017 AHA diagnostic criteria for Kawasaki disease (including complete and incomplete KD); ③ Complete baseline clinical data, including CRP test results at enrollment. All CRP samples were collected at baseline, prior to the initiation of IVIG, glucocorticoids, or any other anti-inflammatory therapy. Patients who met the unified exclusion criteria were excluded.

#### 2.1.13 Healthy adult control subcohort

A subcohort of healthy adult controls was established, including individuals who underwent routine physical examinations at the Physical Examination Center of Nanjing Drum Tower Hospital between January 2021 and January 2023. Inclusion Criteria: ① Age ≥ 18 years; ② No history of acute or chronic inflammatory diseases, autoimmune diseases, or malignant tumors; ③ Normal physical examination results and routine laboratory tests; ④ Complete baseline clinical data, including CRP test results. Individuals who met the unified exclusion criteria were excluded.

#### 2.1.14 Healthy child control subcohort

A subcohort of healthy child controls was established, including children who underwent routine health check-ups at the Department of Child Healthcare of Nanjing Drum Tower Hospital between January 2021 and January 2023. Inclusion Criteria: ① Age < 18 years; ② No history of acute or chronic inflammatory diseases, autoimmune diseases, or congenital anomalies; ③ Normal growth and development, and normal routine laboratory tests; ④ Complete baseline clinical data, including CRP test results. Individuals who met the unified exclusion criteria were excluded.

Noted that patients included in this study were newly diagnosed, and because of this treatment-naïve, cross-sectional baseline design, validated disease activity scores (e.g., Pouchot score for AOSD, BDCAF for Behçet’s disease) could not be reliably calculated, as these instruments require more longitudinal information such as prior flare history or symptom trajectories that were not yet available at the time of diagnosis. Similarly, the distinction between first flare, recurrent flare, and remission phases could not be ascertained. Therefore, we used CRP quartiles and composite indices as objective laboratory-based proxies for inflammatory burden, complemented by dichotomously recorded core clinical manifestations.

### 2.2 Proteomic data processing and analysis

Proteomic analyses were performed using the GSE197546 dataset, which comprises TMT-labeled LC-MS/MS profiles of 170 serum samples from SJIA patients, MAS patients, and healthy controls. Differential expression analysis was performed using the GEO2R online analysis tool (https://www.ncbi.nlm.nih.gov/geo/geo2r/), which applies moderated t-tests with Benjamini-Hochberg correction. The default normalization of the GEO2R platform was applied. Differentially expressed proteins were defined as |log₂(fold change)| > 0.585 and false discovery rate (FDR) < 0.05. For differential expression analysis and all subsequent enrichment analyses (including GO, KEGG, COMPARTMENTS), multiple testing correction was performed using the Benjamini-Hochberg procedure to control the FDR. An adjusted P-value (FDR) < 0.05 was considered statistically significant. Functional enrichment was performed with clusterProfiler. A CRP-centered protein-protein interaction network was constructed using STRING (confidence >0.8, excluding text-mining) and visualized in Cytoscape. For multi-database integration, protein expression data from ProteomicsDB, PaxDb, and MOPED were mapped to UniProt, normalized to log_10_(parts per million), and retained only if detectable in at least two databases, with the median used as the final estimate.

### 2.3 Single-cell and spatial transcriptomic data processing and analysis

Single-cell CITE-seq and spatial transcriptomic data of healthy human liver tissue were downloaded from GEO under accession number GSE192742. For single-cell analysis: raw reads were processed with Cell Ranger (v7.1.0, 10x Genomics) for alignment (GRCh38.p13), gene counting, and quality control. Normalization, dimensionality reduction, clustering, and cell-type annotation were performed using Seurat (v4.3.0) and harmony (v0.1.1) in R. Cell types were annotated with reference to CellMarker (v2.0) and PanglaoDB (v2.0). For spatial transcriptomics: raw data were processed with SpaceRanger (v2.0.0, 10x Genomics). Cell-type deconvolution and spatial mapping were performed using SpatialExperiment (v1.10.0). The spatial expression of CRP was visualized and overlaid onto cell-enriched regions.

### 2.4 Software, databases, and statistical analysis

#### 2.4.1 General software

Basic data processing and visualization were performed using R software (v4.3.1; R Foundation for Statistical Computing), SPSS (v26.0; IBM), and GraphPad Prism (v9.5.1; GraphPad Software). Core R packages used across all analyses included: ggplot2 (v3.4.4), pheatmap (v1.0.1), survey (v4.2-1), scater (v1.28.4), SingleCellExperiment (v1.22.0), DoubletFinder (v2.0.3), rms (v6.7-0), and tidyverse (v2.0.0). Differential expression analysis of proteomic data was performed using the GEO2R online tool, which applies moderated t-tests and Benjamini-Hochberg correction. Reference databases used for functional annotation and sequence alignment included: UniProt human reference proteome (UP000005640), Gene Ontology (GO) database, and Kyoto Encyclopedia of Genes and Genomes (KEGG) database.

#### 2.4.2 Statistical analysis

All statistical analyses were conducted using R software (v4.3.1). The Shapiro-Wilk test was used to assess the normality of continuous variables. Normally distributed data were presented as mean ± standard deviation (SD), while non-normally distributed data were expressed as median (interquartile range, IQR; P25–P75).

For two-group comparisons, the independent-samples t-test (normally distributed data) or Mann-Whitney U test (non-normally distributed data) was applied. For comparisons among three or more independent groups, one-way analysis of variance (ANOVA) with Bonferroni *post hoc* test (normally distributed data) or Kruskal-Wallis H test with Dunn-Bonferroni *post hoc* test (non-normally distributed data) was used. Bivariate correlations were analyzed using Pearson or Spearman rank correlation for normally and non-normally distributed data, respectively.

In our high-throughput omics differential analyses and functional enrichment analyses (e.g., GO, KEGG, COMPARTMENTS), multiple hypothesis testing was corrected using the Benjamini-Hochberg procedure. A false discovery rate (FDR) < 0.05 was applied as the threshold for statistical significance. All tests were two-sided, and P < 0.05 was considered statistically significant.

For subgroup analyses of inflammatory markers and clinical phenotypes, patients with each disease were generally stratified by CRP quartiles. For adult-onset Still’s disease (AOSD), tertile stratification was used to avoid complete separation in the highest CRP quartile. For inflammatory bowel disease (IBD), stratification by the optimal cutoff value was adopted instead of quartile or tertile stratification, as the latter two approaches resulted in insufficient sample size and missing data in individual strata due to the relatively narrow distribution of CRP levels in IBD patients. All stratification strategies were determined prior to formal statistical analysis based on the distribution characteristics of CRP in each disease cohort. The CRP composite indices (CLR and CAR) were calculated as follows. The CRP to lymphocyte ratio (CLR) was serum C reactive protein (CRP, mg/L) divided by absolute lymphocyte count (×10^9^/L). The CRP to albumin ratio (CAR) was serum CRP (mg/L) divided by serum albumin (g/L). Both indices were derived using raw, unstandardized laboratory values, without Z score transformation or differential weighting, because the ratio naturally gives equal weight to both components. This method preserves clinical interpretability and generalizability across different healthcare settings, as it does not rely on population specific reference distributions and requires no additional standardization before routine application. In regression models, these indices were examined both as continuous variables (per 1 unit increase) and as categorical variables stratified by quartiles or tertiles to evaluate dose response relationships. The choice of stratification was based on the distribution of CRP within each disease cohort.

#### 2.4.3 Multi-database bioinformatic analysis

For global protein expression profiling and molecular structure characterization of CRP: Quantitative expression data of human CRP protein were retrieved from ProteomicsDB, PaxDb, and MOPED databases. All expression values were normalized to log_10_(parts per million, ppm) and grouped by functional systems. Three-dimensional molecular structure information of the CRP monomer was retrieved from the PubChem database.

For comprehensive functional annotation of the CRP gene: Multiple datasets were downloaded from the Harmonizome database (37) (all searches completed in March 2026): 1. Achilles Cell Line Gene Essentiality Profiles: Analyzed the effect of CRP knockdown on tumor cell proliferation fitness (Z-score ≥1.5 or ≤-1.5 defined as significant); 2. ChEA Transcription Factor Targets 2022: Screened transcription factors directly binding to the CRP promoter; 3. CMAP Signatures: Identified small molecules altering CRP transcription (|log₂FC| ≥0.5, adjusted P <0.05); 4. CTD Gene-Chemical Interactions: Retrieved experimentally verified chemicals interacting with CRP; 5. DISEASES Curated Associations: Extracted high-confidence disease associations (evidence score ≥3).

## 3 Results

### 3.1 Expression and clinical significance of CRP in AOSD

#### 3.1.1 Baseline demographic, clinical, and laboratory characteristics of AOSD patients and controls

A total of 812 patients were included in the study, consisting of 406 patients with AOSD and 406 non-AOSD controls. The two groups were matched by age and gender (**Table 1**). No significant differences were observed between the groups in terms of age, gender, body mass index (BMI), smoking status, alcohol use, prevalence of hypertension (HTN), or diabetes mellitus (DM) (P > 0.05 for each).

**Table 1:** Baseline demographic, clinical, and laboratory characteristics of AOSD and non-AOSD control population.

Variables	Total (n = 812)	Non-AOSD controls (n = 406)	AOSD patients (n = 406)	Statistic	P
CRP, M (Q1, Q3)	7.00 (2.80, 55.75)	3.40 (1.90, 5.90)	55.75 (13.85, 101.42)	Z=-19.38	<.001
ESR, M (Q1, Q3)	27.50 (12.00, 64.00)	14.00 (8.00, 23.00)	50.00 (20.50, 80.00)	Z=-11.46	<.001
IL6, M (Q1, Q3)	6.87 (3.45, 20.48)	5.81 (2.90, 15.07)	18.76 (7.76, 50.30)	Z=-5.05	<.001
CLR, M (Q1, Q3)	5.60 (1.85, 39.85)	2.33 (0.96, 4.79)	40.79 (10.83, 90.62)	Z=-18.43	<.001
Age, M (Q1, Q3)	42.00 (32.00, 55.00)	42.00 (33.00, 56.00)	42.00 (31.00, 53.00)	Z=-1.68	0.094
Gender, n (%)				χ2=0.00	1.000
Male	322 (39.66)	161 (39.66)	161 (39.66)		
Female	490 (60.34)	245 (60.34)	245 (60.34)		
Smoking, n (%)				χ2=2.56	0.110
No	625 (93.98)	357 (92.73)	268 (95.71)		
Yes	40 (6.02)	28 (7.27)	12 (4.29)		
Drinking, n (%)				χ2=2.14	0.144
No	644 (94.99)	373 (93.95)	271 (96.44)		
Yes	34 (5.01)	24 (6.05)	10 (3.56)		
BMI, M (Q1, Q3)	23.60 (21.12, 26.00)	23.80 (21.50, 26.00)	23.40 (20.60, 25.50)	Z=-1.55	0.122
HTN, n (%)				χ2=1.20	0.273
No	718 (88.42)	354 (87.19)	364 (89.66)		
Yes	94 (11.58)	52 (12.81)	42 (10.34)		
DM, n (%)				χ2=2.40	0.121
No	720 (88.67)	353 (86.95)	367 (90.39)		
Yes	92 (11.33)	53 (13.05)	39 (9.61)		

AOSD, adult-onset Still’s disease; CRP, C-reactive protein; CLR, CRP/lymphocyte ratio; ESR, erythrocyte sedimentation rate; IL-6, interleukin-6; BMI, body mass index; HTN, hypertension; DM, diabetes mellitus; M, median; Q1, first quartile; Q3, third quartile.

As the primary inflammatory marker of interest, serum CRP levels were significantly higher in the AOSD group than in the non-AOSD controls (P < 0.001). Consistent with systemic inflammatory activation, other conventional inflammatory indicators, such as erythrocyte sedimentation rate (ESR) and interleukin 6 (IL-6), were also elevated in AOSD patients (P < 0.001 for each). Notably, the CRP to lymphocyte ratio (CLR), a composite index that combines CRP concentration with lymphocyte count, was significantly higher in the AOSD group. CLR may help address the lack of specificity of CRP alone by reflecting both systemic inflammatory intensity and immune cell status, and thus appears to offer improved sensitivity for assessing the inflammatory burden in AOSD.

In addition to inflammatory markers, AOSD patients showed a distinct laboratory profile, characterized by higher levels of serum ferritin, D dimer (D-D), fibrinogen (Fbg), white blood cell (WBC) count, neutrophil count, monocyte count, platelet count, complement C3, complement C4, immunoglobulin E (IgE), immunoglobulin M (IgM), lactate dehydrogenase (LDH), alanine transaminase (ALT), aspartate transaminase (AST), and estimated glomerular filtration rate (eGFR). In contrast, lymphocyte count, eosinophil count, and hemoglobin concentration were lower in the AOSD group (P < 0.05 for each). The two groups also differed in the rate of urine occult blood positivity (P < 0.001), whereas no significant between-group differences were observed for urine protein positivity or antinuclear antibody (ANA) positivity (**Supplementary Table 1**).

#### 3.1.2 Correlations between CRP levels and clinical manifestations in AOSD patients

To further explore the clinical relevance of CRP in AOSD, patients were divided into four subgroups based on baseline CRP quartiles (Q1 to Q4). As shown in **Table 2**, the frequencies of core clinical manifestations of AOSD, including fever, evanescent rash, and arthralgia, were higher in patients with elevated CRP levels (P < 0.05 for each, χ^2^ test). These results suggest that CRP levels are associated with disease activity and phenotypic severity in AOSD.

**Table 2:** Clinical manifestations and inflammatory cytokine profiles in AOSD patients stratified by CRP quartiles.

Variables	Total (n = 406)	CRP Q1 (n = 102)	CRP Q2 (n = 101)	CRP Q3 (n = 101)	CRP Q4 (n = 102)	Statistic	P
Symptom Duration (in weeks), n (%)						χ2=6.24	0.397
Duration T1	122 (30.05)	33 (32.35)	30 (29.70)	24 (23.76)	35 (34.31)		
Duration T2	121 (29.80)	33 (32.35)	34 (33.66)	30 (29.70)	24 (23.53)		
Duration T3	163 (40.15)	36 (35.29)	37 (36.63)	47 (46.53)	43 (42.16)		
Fever, n (%)						χ2=11.10	0.011
No	32 (8.25)	12 (12.77)	3 (3.12)	13 (13.00)	4 (4.08)		
Yes	356 (91.75)	82 (87.23)	93 (96.88)	87 (87.00)	94 (95.92)		
Rash, n (%)						χ2=25.53	<.001
No	174 (44.85)	63 (67.02)	38 (39.58)	34 (34.00)	39 (39.80)		
Yes	214 (55.15)	31 (32.98)	58 (60.42)	66 (66.00)	59 (60.20)		
Arthralgia, n (%)						χ2=22.53	<.001
No	231 (59.54)	73 (77.66)	59 (61.46)	55 (55.00)	44 (44.90)		
Yes	157 (40.46)	21 (22.34)	37 (38.54)	45 (45.00)	54 (55.10)		
TNF-α, M (Q1, Q3)	3.25 (1.68, 4.57)	1.43 (0.81,2.04)	9.84 (5.31,12.88)	3.04 (2.26,3.83)	3.47 (2.27,3.59)	H=2.90	0.407
IFN-γ, M (Q1, Q3)	10.66 (8.40, 18.00)	15.48 (11.87,19.09)	19.62 (9.90,39.20)	10.86 (9.64,12.38)	9.97 (8.39,10.46)	H=1.35	0.718
PCT, M (Q1, Q3)	0.17 (0.08, 0.33)	0.07 (0.05,0.23)	0.17 (0.09,0.43)	0.12 (0.07,0.26)	0.24 (0.12,0.34)	H=10.52	0.015

AOSD, adult-onset Still’s disease; CRP, C-reactive protein; TNF-α, tumor necrosis factor-α; IFN-γ, interferon-γ; PCT, procalcitonin; M, median; Q1, first quartile; Q3, third quartile.

Among inflammatory mediators, serum procalcitonin (PCT) levels differed across the four CRP subgroups (H = 10.52, P = 0.015, Kruskal-Wallis H test). Given that PCT is a standard clinical marker for ruling out bacterial infection, this finding supports the view that the elevated CRP levels observed in our cohort were primarily driven by AOSD disease activity rather than concurrent infection. On the other hand, no significant differences were observed in tumor necrosis factor α (TNF-α) or interferon γ (IFN-γ) levels across CRP quartiles, suggesting that CRP may reflect a dimension of systemic inflammation that is distinct from these cytokines.

#### 3.1.3 Independent associations of elevated CRP and CLR with increased risk of AOSD

Univariate and multivariable logistic regression analyses were performed to evaluate the independent effects of CRP and CLR on AOSD risk. Due to quasi-complete separation in the highest CRP tertile, standard logistic regression produced unstable estimates. We therefore applied Firth’s penalized logistic regression to provide bias-reduced estimates. As shown in **Table 3**, compared with the lowest CRP tertile (T1, 0.1-3.8 mg/L), both the middle (T2, 3.9-27.3 mg/L) and highest (T3, 27.4-330.4 mg/L) tertiles were associated with an increased risk of AOSD (P < 0.001 for each). The fully adjusted OR for the highest tertile was 970.1 (95% CI: 304.7-4928.0), with a positive dose–response trend (P for trend < 0.001). No control subjects had CRP levels exceeding 150 mg/L, suggesting that markedly elevated CRP may be relatively specific to AOSD in this treatment-naive cohort.

**Table 3:** Association between CRP tertiles and risk of AOSD in logistic regression models (Firth penalized regression).

Exposure	Non-adjusted	Adjust I	Adjust II
CRP group			
T1 (0.1 - 3.8)	ref	ref	ref
T2 (3.9 - 27.3)	2.0 (1.3, 3.0) <0.001	2.1 (1.4, 3.2) <0.001	3.6 (2.0, 6.5) <0.001
T3 (27.4 - 330.4)	472.0 (159.1, 2304.3) <0.001	518.8 (173.1, 2546.7) <0.001	970.1 (304.7, 4928.0) <0.001
CRP group trend	9.3 (7.1, 12.5) <0.001	9.7 (7.3, 13.1) <0.001	25.0 (16.0, 41.5) <0.001
Sample Size	812	810	664

Data are presented as odds ratio (OR) with 95% confidence interval (CI). Adjust I: adjusted for age and sex. Adjust II: adjusted for age, sex, smoking, drinking, HTN, and DM. Due to quasi-complete separation in the highest CRP tertile, Firth’s penalized maximum likelihood logistic regression was applied to provide bias-reduced estimates. AOSD, adult-onset Still’s disease; CRP, C-reactive protein; HTN, hypertension; DM, diabetes mellitus; ref, reference.

As a composite inflammatory marker, CLR also showed an association with AOSD risk (**Table 4**). Compared with the lowest CLR tertile (T1), the highest tertile (T3) was associated with increased odds of AOSD (fully adjusted OR = 2.9, 95% CI: 2.0-4.2, P < 0.001), while the middle tertile (T2) showed no significant association (fully adjusted OR = 1.2, 95% CI: 0.3-4.6, P = 0.796). A positive trend was observed across increasing CLR tertiles (P for trend < 0.001).

**Table 4:** Association between CLR tertiles and risk of AOSD in logistic regression models (Firth penalized regression).

Exposure	Non-adjusted	Adjust I	Adjust II
CLR group			
T1	ref	ref	ref
T2	1.1 (0.3, 3.7) 0.936	1.1 (0.3, 4.0) 0.867	1.2 (0.3, 4.6) 0.796
T3	2.8 (2.0, 3.9) <0.001	2.9 (2.0, 4.0) <0.001	2.9 (2.0, 4.2) <0.001
CLR group trend	1.7 (1.4, 2.0) <0.001	1.7 (1.4, 2.0) <0.001	1.7 (1.4, 2.0) <0.001
Sample Size	812	810	664

Data are presented as odds ratio (OR) with 95% confidence interval (CI). Adjust I: adjusted for age and sex. Adjust II: adjusted for age, sex, smoking, drinking, HTN, and DM. Firth’s penalized maximum likelihood logistic regression was applied to provide bias-reduced estimates. CLR, C-reactive protein to lymphocyte ratio; AOSD, adult-onset Still’s disease; HTN, hypertension; DM, diabetes mellitus; ref, reference. Due to quasi-complete separation in the highest CRP/CLR tertile, standard logistic regression produced inflated odds ratio estimates (CRP T3 OR > 1000). We therefore applied Firth’s penalized maximum likelihood logistic regression, which yielded substantially reduced and more stable estimates.

#### 3.1.4 Forest plot visualization of CRP-related risk for AOSD

**Figure 1:**
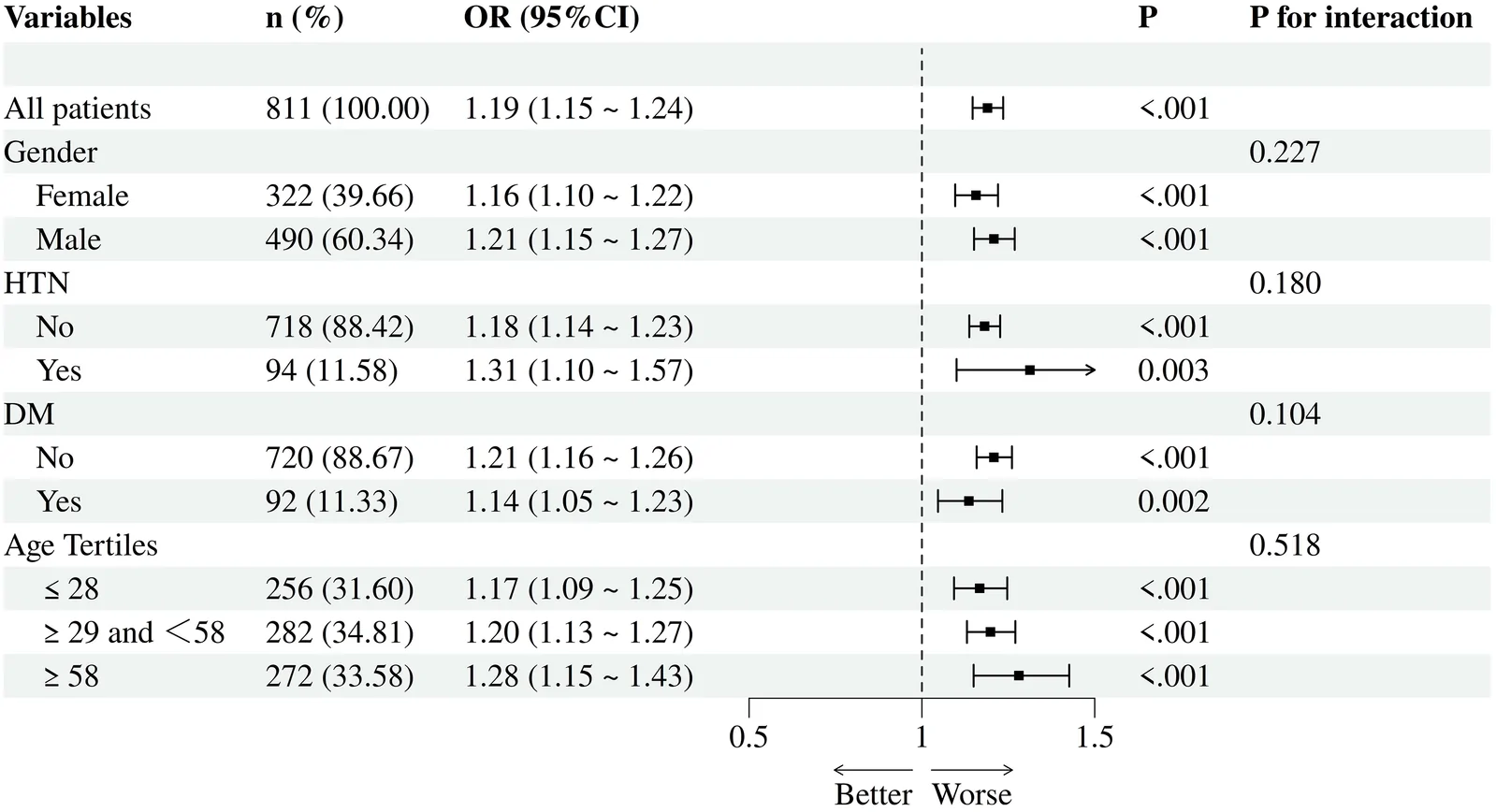
Forest plot of the association between serum CRP (per 1 mg/L increment) and AOSD risk. ORs and 95% CIs were derived from fully adjusted models (adjusted for age, sex, smoking, drinking, hypertension, and diabetes). Subgroups were stratified by gender, hypertension (HTN), diabetes mellitus (DM), and age tertiles. P for interaction indicates heterogeneity across subgroups. AOSD, adult-onset Still’s disease; CRP, C-reactive protein; OR, odds ratio; CI, confidence interval.

The forest plot (**Figure 1**) shows the association between serum CRP (per 1 mg/L increment) and AOSD risk across clinically relevant subgroups, based on fully adjusted logistic regression models. In the overall cohort (n = 811), each 1 mg/L increase in CRP was independently associated with a 19% higher risk of AOSD (OR = 1.19, 95% CI: 1.15–1.24, P < 0.001).

Positive associations were also observed across the following subgroups: females (OR = 1.16, P < 0.001), males (OR = 1.21, P < 0.001), non-hypertensive (OR = 1.18, P < 0.001), hypertensive (OR = 1.31, P = 0.003), non-diabetic (OR = 1.21, P < 0.001), diabetic (OR = 1.14, P = 0.002), and all age tertiles (OR ranging from 1.17 to 1.28, P < 0.001 for each). No significant effect modifications were detected for gender (P for interaction = 0.227), hypertension (P = 0.180), diabetes (P = 0.104), or age (P = 0.518), suggesting that CRP is a relatively stable independent risk marker for AOSD across these subgroups.

### 3.2 Expression and clinical significance of CRP in BD

#### 3.2.1 Baseline demographic, clinical, and laboratory characteristics of BD patients and controls

A total of 1058 subjects were enrolled in this section, including 513 BD patients and 545 non-BD controls. The two groups were comparable in terms of baseline demographic and clinical characteristics. No significant differences were observed in age, gender distribution, smoking status, alcohol use, body mass index (BMI), prevalence of hypertension (HTN), or diabetes mellitus (DM) (P > 0.05 for each).

Serum CRP levels were higher in BD patients than in non-BD controls [4.80 (2.80, 29.30) vs. 3.90 (2.30, 7.22), Z = -6.11, P < 0.001]. Consistent with increased systemic inflammation, erythrocyte sedimentation rate (ESR) was also elevated in the BD group (Z = -8.45, P < 0.001), whereas interleukin-6 (IL-6) levels showed no significant difference between the two groups (Z = -0.91, P = 0.363). The CRP to albumin ratio (CAR) combines CRP and serum albumin, two indicators that tend to change in opposite directions during systemic inflammation. By taking into account both inflammatory activation and nutritional-immune status, CAR may help reduce the influence of fluctuations in any single index and serve as a more stable and comprehensive marker for assessing systemic inflammation in BD (**Table 5**).

**Table 5:** Baseline demographic, clinical, and laboratory characteristics of BD and non-BD control population.

Variables	Total (n = 1058)	Non-BD controls (n = 545)	BD patients (n = 513)	Statistic	P
CRP, M (Q1, Q3)	4.33 (2.59, 11.38)	3.90 (2.30, 7.22)	4.80 (2.80, 29.30)	Z=-6.11	<.001
ESR, M (Q1, Q3)	16.00 (7.00, 38.00)	11.00 (5.00, 20.00)	27.00 (11.00, 53.00)	Z=-8.45	<.001
IL6, M (Q1, Q3)	8.01 (3.70, 20.90)	6.32 (3.14, 16.55)	8.42 (3.79, 24.39)	Z=-0.91	0.363
CAR, M (Q1, Q3)	0.11 (0.06, 0.37)	0.07 (0.04, 0.17)	0.13 (0.07, 0.81)	Z=-8.37	<.001
CLR, M (Q1, Q3)	2.75 (1.34, 10.17)	1.85 (0.96, 4.49)	3.59 (1.68, 20.76)	Z=-8.14	<.001
Age, M (Q1, Q3)	41.00 (32.00, 53.00)	40.00 (31.00, 53.00)	43.00 (33.00, 53.00)	Z=-1.95	0.052
Gender, n (%)				χ2=0.03	0.858
Male	459 (43.38)	235 (43.12)	224 (43.66)		
Female	599 (56.62)	310 (56.88)	289 (56.34)		
Smoking, n (%)				χ2=0.55	0.457
No	631 (92.25)	160 (93.57)	471 (91.81)		
Yes	53 (7.75)	11 (6.43)	42 (8.19)		
Drinking, n (%)				χ2=0.46	0.497
No	659 (96.20)	164 (95.35)	495 (96.49)		
Yes	26 (3.80)	8 (4.65)	18 (3.51)		
BMI, M (Q1, Q3)	22.20 (20.12, 24.35)	22.05 (19.78, 23.90)	22.45 (20.40, 24.65)	Z=-1.80	0.072
HTN, n (%)				χ2=1.66	0.198
No	914 (86.39)	478 (87.71)	436 (84.99)		
Yes	144 (13.61)	67 (12.29)	77 (15.01)		
DM, n (%)				χ2=3.71	0.054
No	961 (90.83)	486 (89.17)	475 (92.59)		
Yes	97 (9.17)	59 (10.83)	38 (7.41)		

BD, Behçet’s disease; CRP, C-reactive protein; CAR, CRP/albumin ratio; CLR, CRP/lymphocyte ratio; ESR, erythrocyte sedimentation rate; IL-6, interleukin-6; BMI, body mass index; HTN, hypertension; DM, diabetes mellitus; M, median; Q1, first quartile; Q3, third quartile.

Other laboratory findings, as presented in **Supplementary Table 2**, showed that BD patients had lower lymphocyte count, hemoglobin concentration, platelet count, albumin level, complement C3, and complement C4, along with higher immunoglobulin E, alanine transaminase (ALT), and aspartate transaminase (AST) levels (P < 0.05 for each). No significant differences were observed between the two groups in white blood cell count, neutrophil count, uric acid, urea, or estimated glomerular filtration rate (eGFR) (P > 0.05 for each).

#### 3.2.2 Correlations between CRP levels and clinical manifestations in BD patients

To explore the clinical relevance of CRP in BD, patients were stratified into four subgroups according to CRP quartiles (Q1–Q4). There were no significant differences in disease duration among the four CRP subgroups (P > 0.05). Similarly, the frequencies of core clinical manifestations of BD, including oral ulcers, genital ulcers, and ocular involvement, were comparable across CRP quartiles (all P > 0.05). These results suggest that serum CRP levels are not closely associated with the occurrence of typical mucosal and ocular phenotypes in BD, but mainly reflect the degree of systemic inflammatory burden (**Table 6**).

**Table 6:** Clinical manifestations in BD patients stratified by CRP quartiles.

Variables	Total (n = 513)	CRP Q1 (n = 126)	CRP Q2 (n = 130)	CRP Q3 (n = 128)	CRP Q4 (n = 129)	Statistic	P
Symptom duration (in months), n (%)						χ2=2.79	0.834
Duration T1	108 (21.05)	26 (20.63)	27 (20.77)	25 (19.53)	30 (23.26)		
Duration T2	204 (39.77)	48 (38.10)	55 (42.31)	47 (36.72)	54 (41.86)		
Duration T3	201 (39.18)	52 (41.27)	48 (36.92)	56 (43.75)	45 (34.88)		
Oral Ulcers, n (%)						χ2=3.76	0.288
No	194 (37.82)	43 (34.13)	58 (44.62)	48 (37.50)	45 (34.88)		
Yes	319 (62.18)	83 (65.87)	72 (55.38)	80 (62.50)	84 (65.12)		
Genital Ulcers, n (%)						χ2=0.59	0.899
No	385 (75.05)	97 (76.98)	98 (75.38)	96 (75.00)	94 (72.87)		
Yes	128 (24.95)	29 (23.02)	32 (24.62)	32 (25.00)	35 (27.13)		
Ocular Symptoms, n (%)						χ2=1.43	0.697
No	243 (47.37)	64 (50.79)	57 (43.85)	59 (46.09)	63 (48.84)		
Yes	270 (52.63)	62 (49.21)	73 (56.15)	69 (53.91)	66 (51.16)		

BD, Behçet’s disease; CRP, C-reactive protein; M, median; Q1, first quartile; Q3, third quartile.

#### 3.2.3 Independent associations of elevated CRP, CAR and CLR with increased risk of BD

Univariate and multivariable logistic regression analyses were performed to evaluate the independent effects of CRP, CAR, and CLR on BD risk. For CRP, compared with the lowest quartile (Q1), the second (Q2) and fourth (Q4) quartiles were significantly associated with an increased risk of BD in the unadjusted model, age-sex adjusted model, and fully adjusted model (all P < 0.05). A significant positive dose–response relationship was observed across ascending CRP categories (P for trend < 0.001) (**Table 7**).

**Table 7:** Association between CRP quartiles and risk of BD in logistic regression models.

Exposure	Non-adjusted	Adjust I	Adjust II
CRP group			
Q1 (0.08 - 2.59)	ref	ref	ref
Q2 (2.6 - 4.3)	1.6 (1.1, 2.2) 0.012	1.5 (1.1, 2.2) 0.014	2.3 (1.4, 4.0) 0.002
Q3 (4.33 - 11.3)	0.8 (0.6, 1.2) 0.323	0.8 (0.6, 1.2) 0.317	0.8 (0.5, 1.3) 0.388
Q4 (11.4 - 280.6)	3.5 (2.5, 5.0) <0.001	3.6 (2.5, 5.1) <0.001	4.0 (2.3, 6.9) <0.001
CRP group trend	<0.001	<0.001	<0.001
Sample Size	1058	1058	684

Data are presented as odds ratio (OR) with 95% confidence interval (CI). Adjust I: adjusted for age and sex. Adjust II: adjusted for age, sex, smoking, drinking, HTN, and DM. BD, Behçet’s disease; CRP, C-reactive protein; ref, reference.

Both CAR and CLR showed robust associations with BD risk. Compared with the lowest quartile (Q1), the highest quartiles (Q4) of CAR and CLR conferred substantially elevated odds of BD in all regression models (all P < 0.001), with significant positive trends across graded subgroups (all P for trend < 0.001). These findings indicate that CAR and CLR, as integrated inflammatory biomarkers, have improved performance in identifying individuals at high risk of BD (**Table 8**).

**Table 8:** Association between CAR quartiles, CLR quartiles and risk of BD in logistic regression models.

Exposure	Non-adjusted	Adjust I	Adjust II
CAR group			
Q1	ref	ref	ref
Q2	2.2 (1.5, 3.3) <0.001	2.1 (1.4, 3.1) <0.001	2.8 (1.6, 5.2) <0.001
Q3	1.5 (1.0, 2.3) 0.036	1.5 (1.0, 2.2) 0.045	1.1 (0.7, 1.9) 0.689
Q4	6.4 (4.0, 10.4) <0.001	6.6 (4.1, 10.6) <0.001	5.4 (2.8, 10.3) <0.001
CAR group trend	<0.001	<0.001	<0.001
Sample size	801	801	641
CLR group			
Q1	ref	ref	ref
Q2	1.8 (1.2, 2.7) 0.004	1.8 (1.2, 2.6) 0.006	1.7 (1.0, 3.0) 0.057
Q3	1.8 (1.2, 2.7) 0.004	1.8 (1.2, 2.7) 0.004	1.4 (0.8, 2.4) 0.198
Q4	6.3 (3.9, 10.1) <0.001	6.4 (3.9, 10.3) <0.001	4.9 (2.6, 9.5) <0.001
CLR group trend	<0.001	<0.001	<0.001
Sample size	799	799	640

Data are presented as odds ratio (OR) with 95% confidence interval (CI). Adjust I: adjusted for age and sex. Adjust II: adjusted for age, sex, smoking, drinking, HTN, and DM. BD, Behçet’s disease; CAR, CRP/albumin ratio; CLR, CRP/lymphocyte ratio; ref, reference.

#### 3.2.4 Subgroup analysis and forest plot visualization of CRP-related risk for BD

**Figure 2:**
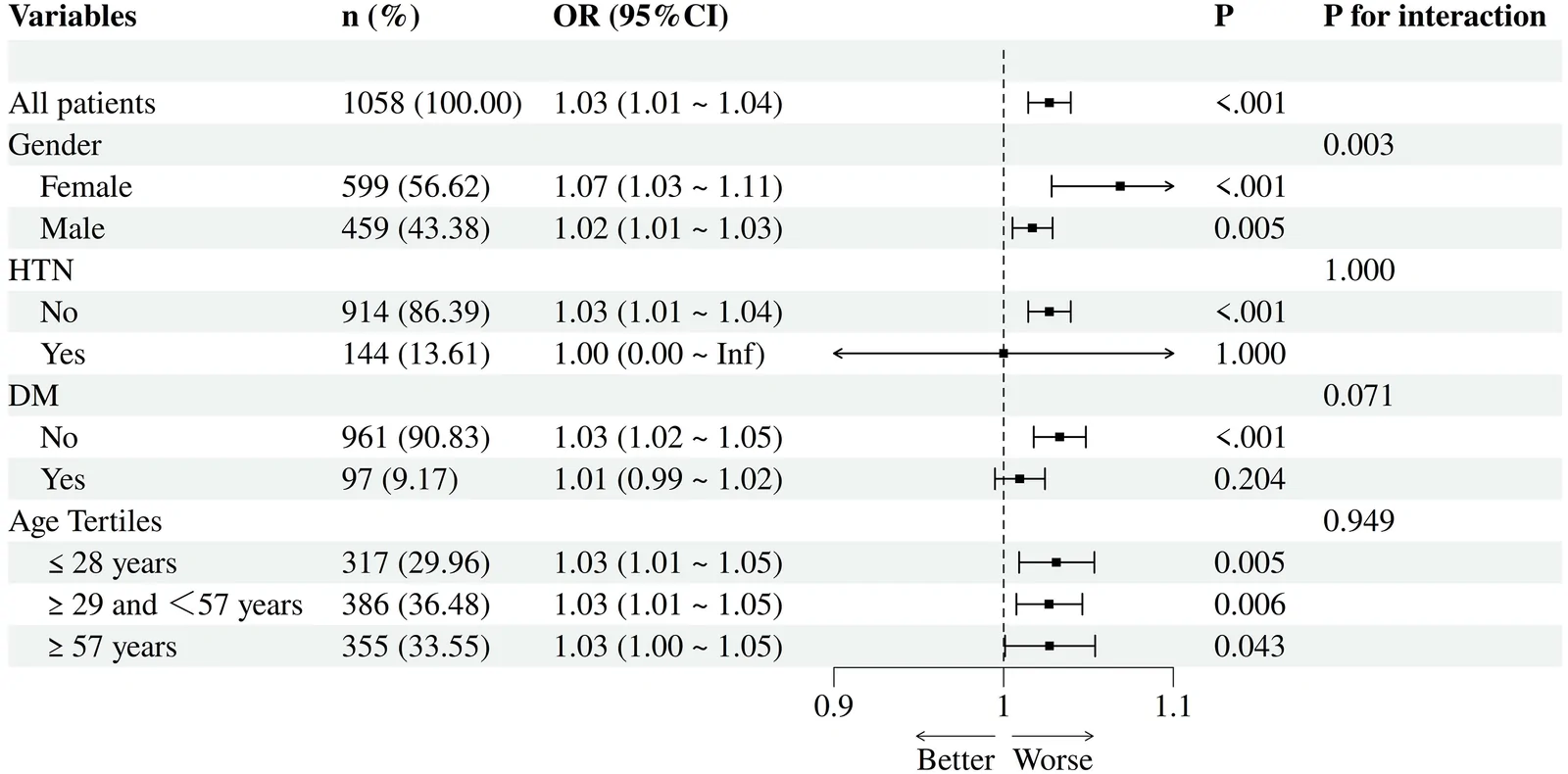
Forest plot of the association between serum CRP (per 1 mg/L increment) and risk of BD. ORs and 95% CIs were derived from fully adjusted logistic regression models adjusted for age, sex, smoking, drinking, hypertension, and diabetes. Subgroups were stratified by gender, hypertension (HTN), diabetes mellitus (DM), and age tertiles. The infinitely wide confidence interval (Inf) in the HTN subgroup arises from complete statistical separation due to extreme event distribution in this small sample. P for interaction indicates heterogeneity across subgroups. BD, Behçet’s disease; CRP, C-reactive protein; OR, odds ratio; CI, confidence interval; HTN, hypertension; DM, diabetes mellitus; Inf, infinity.

The forest plot (**Figure 2**) shows the association between serum CRP (per 1 mg/L increment) and BD risk across different subgroups, based on fully adjusted logistic regression models. In the overall cohort (n = 1058), each 1 mg/L increase in CRP was independently associated with a 3% higher risk of BD (OR = 1.03, 95% CI: 1.01–1.04, P < 0.001).

A gender-dependent interaction was detected (P for interaction = 0.003). The association was more pronounced in females (n = 599, 56.62%; OR = 1.07, 95% CI: 1.03–1.11, P < 0.001) than in males (n = 459, 43.38%; OR = 1.02, 95% CI: 1.01–1.03, P = 0.005). In analyses stratified by hypertension (HTN), the association remained significant in non-hypertensive patients (n = 914, 86.39%; OR = 1.03, 95% CI: 1.01–1.04, P < 0.001). In the HTN subgroup (n = 144, 13.61%), all individuals with elevated CRP levels were diagnosed with BD, leading to complete statistical separation. This resulted in non-convergence of maximum likelihood estimation and an infinitely wide 95% confidence interval (OR = 1.00, 95% CI: 0.00–Inf, P = 1.000), with no significant between-group interaction (P for interaction = 1.000).

No significant interactions were observed for diabetes mellitus (DM, P for interaction = 0.071) or age tertiles (P for interaction = 0.949). Positive associations were seen in non-diabetic patients (OR = 1.03, P < 0.001) and across all age groups (OR = 1.03, P < 0.05 for each), while the diabetic subgroup showed no statistically significant association (OR = 1.01, P = 0.204). These findings suggest that CRP may serve as a gender-specific biomarker for BD risk assessment, with particular clinical value in female patients.

### 3.3 Expression and clinical significance of CRP SpA

#### 3.3.1 Baseline demographic, clinical, and laboratory characteristics of SpA patients and controls

A total of 665 subjects were enrolled, including 377 SpA patients and 288 non-SpA controls. Baseline demographic and clinical characteristics were generally balanced between groups. Serum CRP levels were significantly higher in SpA patients than in controls [4.60 (2.50, 12.50) vs. 3.05 (2.20, 4.93), Z = -5.34, P < 0.001]. IL-6 was also elevated (Z = -2.51, P = 0.012), while ESR showed no significant difference (Z = -1.83, P = 0.067).

CAR and CLR were both significantly increased in SpA patients (CAR: Z = -4.08, P < 0.001; CLR: Z = -4.57, P < 0.001). These composite indices improve the stability of inflammatory assessment by combining CRP with nutritional or immune cell parameters, thereby enhancing the accuracy of systemic inflammation evaluation in SpA (**Table 9**).

**Table 9:** Baseline demographic, clinical, and laboratory characteristics of SpA and non-SpA control population.

Variables	Total (n = 665)	Non-SpA controls (n = 288)	SpA patients (n = 377)	Statistic	P
CRP, M (Q1, Q3)	3.70 (2.30, 8.10)	3.05 (2.20, 4.93)	4.60 (2.50, 12.50)	Z=-5.34	<.001
ESR, M (Q1, Q3)	9.00 (5.00, 19.00)	8.00 (4.25, 14.00)	9.00 (5.00, 21.00)	Z=-1.83	0.067
IL6, M (Q1, Q3)	8.23 (4.21, 20.00)	5.44 (3.47, 9.51)	12.00 (7.43, 21.43)	Z=-2.51	0.012
CAR, M (Q1, Q3)	0.08 (0.05, 0.18)	0.07 (0.05, 0.13)	0.10 (0.05, 0.30)	Z=-4.08	<.001
CLR, M (Q1, Q3)	2.00 (1.13, 4.58)	1.68 (1.00, 3.10)	2.28 (1.29, 6.80)	Z=-4.57	<.001
Age, M (Q1, Q3)	34.00 (27.00, 43.00)	36.00 (29.00, 44.00)	34.00 (26.00, 42.00)	Z=-2.39	0.017
Gender, n (%)				χ2=0.00	0.950
Male	387 (58.20)	168 (58.33)	219 (58.09)		
Female	278 (41.80)	120 (41.67)	158 (41.91)		
Smoking, n (%)				χ2=1.22	0.270
No	424 (91.97)	259 (90.88)	165 (93.75)		
Yes	37 (8.03)	26 (9.12)	11 (6.25)		
Drinking, n (%)				χ2=0.01	0.909
No	443 (95.89)	274 (95.80)	169 (96.02)		
Yes	19 (4.11)	12 (4.20)	7 (3.98)		
BMI, M (Q1, Q3)	23.80 (21.20, 26.45)	23.90 (21.30, 26.58)	22.40 (20.50, 25.70)	Z=-1.95	0.052
HTN, n (%)				χ2=1.74	0.187
No	636 (95.64)	272 (94.44)	364 (96.55)		
Yes	29 (4.36)	16 (5.56)	13 (3.45)		
DM, n (%)				χ2=4.43	0.035
No	638 (95.94)	271 (94.10)	367 (97.35)		
Yes	27 (4.06)	17 (5.90)	10 (2.65)		

SpA, spondyloarthritis; CRP, C-reactive protein; CAR, CRP/albumin ratio; CLR, CRP/lymphocyte ratio; ESR, erythrocyte sedimentation rate; IL-6, interleukin-6; BMI, body mass index; HTN, hypertension; DM, diabetes mellitus; M, median; Q1, first quartile; Q3, third quartile.

Laboratory profiling showed significantly higher WBC count, neutrophil count, platelet count, complement C3, albumin, AST, and eGFR in SpA patients, along with lower uric acid (all P < 0.05). No significant differences were observed in lymphocyte count, hemoglobin, or urine test results (all P > 0.05) (**Supplementary Table 3**).

#### 3.3.2 Correlations between CRP levels and clinical manifestations in SpA patients

Patients were stratified into four subgroups by CRP quartiles (Q1–Q4). No significant differences were observed in disease duration across subgroups (P > 0.05). The frequencies of inflammatory back pain, peripheral arthritis, and enthesitis were comparable among CRP subgroups (all P > 0.05). Similarly, HLA-B27 positivity rates did not differ significantly across quartiles (P > 0.05). These findings indicate that CRP levels reflect systemic inflammatory intensity but are not closely associated with the occurrence of typical clinical phenotypes in SpA (**Table 10**).

**Table 10:** Clinical manifestations in SpA patients stratified by CRP quartiles.

Variables	Total (n = 377)	CRP Q1 (n = 91)	CRP Q2 (n = 94)	CRP Q3 (n = 97)	CRP Q4 (n = 95)	Statistic	P
Symptom Duration (in months), n ( %)						χ2=1.38	0.967
Duration T1	105 (27.85)	23 (25.27)	26 (27.66)	26 (26.80)	30 (31.58)		
Duration T2	146 (38.73)	38 (41.76)	35 (37.23)	39 (40.21)	34 (35.79)		
Duration T3	126 (33.42)	30 (32.97)	33 (35.11)	32 (32.99)	31 (32.63)		
Inflammatory Back Pain, n (%)						χ2=3.82	0.282
No	46 (31.08)	9 (30.00)	8 (22.22)	18 (41.86)	11 (28.21)		
Yes	102 (68.92)	21 (70.00)	28 (77.78)	25 (58.14)	28 (71.79)		
Peripheral Arthritis, n (%)						χ2=1.89	0.596
No	89 (62.24)	15 (53.57)	25 (69.44)	27 (64.29)	22 (59.46)		
Yes	54 (37.76)	13 (46.43)	11 (30.56)	15 (35.71)	15 (40.54)		
Enthesitis, n (%)						χ2=3.09	0.378
No	108 (73.97)	25 (80.65)	28 (77.78)	27 (64.29)	28 (75.68)		
Yes	38 (26.03)	6 (19.35)	8 (22.22)	15 (35.71)	9 (24.32)		
HLA-B27, n (%)						χ2=1.56	0.668
No	24 (24.00)	5 (20.83)	8 (33.33)	5 (22.73)	6 (20.00)		
Yes	76 (76.00)	19 (79.17)	16 (66.67)	17 (77.27)	24 (80.00)		

SpA, spondyloarthritis; CRP, C-reactive protein; M, median; Q1, first quartile; Q3, third quartile.

#### 3.3.3 Independent associations of elevated CRP, CAR and CLR with increased risk of SpA

Univariate and multivariable logistic regression analyses were performed. Compared with the lowest CRP quartile (Q1), the highest quartile (Q4) was independently associated with elevated SpA risk in all models (OR = 3.9, 95% CI: 2.2–7.0, P < 0.001), with a significant positive dose–response trend (P for trend < 0.001) (**Table 11**).

**Table 11:** Association between CRP quartiles and risk of SpA in logistic regression models.

Exposure	Non-adjusted	Adjust I	Adjust II	
CRP group				
Q1 (0.1 - 2.2)	ref	ref	ref
Q2 (2.3 - 3.6)	0.7 (0.5, 1.1) 0.117	0.7 (0.5, 1.1) 0.115	0.8 (0.5, 1.5) 0.513	
Q3 (3.7 - 8)	1.4 (0.9, 2.2) 0.115	1.4 (0.9, 2.2) 0.113	1.7 (1.0, 3.0) 0.065	
Q4 (8.1 - 169.6)	2.9 (1.8, 4.6) <0.001	3.0 (1.9, 4.8) <0.001	3.9 (2.2, 7.0) <0.001	
CRP group trend	<0.001	<0.001	<0.001
Sample Size	665	665	461

Data are presented as odds ratio (OR) with 95% confidence interval (CI). Adjust I: adjusted for age and sex. Adjust II: adjusted for age, sex, smoking, drinking, HTN, and DM. SpA, spondyloarthritis; CRP, C-reactive protein; ref, reference.

CAR and CLR showed similarly robust predictive value. The highest quartiles of CAR (Q4) and CLR (Q4) were both strongly associated with SpA risk after full adjustment (all P < 0.01), with significant positive trends across subgroups (all P for trend < 0.001). These results confirm that CAR and CLR serve as reliable composite biomarkers for SpA risk stratification (**Table 12**).

**Table 12:** Association between CAR quartiles, CLR quartiles and risk of SpA in logistic regression models.

Exposure	Non-adjusted	Adjust I	Adjust II
CAR group			
Q1	ref	ref	ref
Q2	0.6 (0.4, 1.1) 0.079	0.6 (0.4, 1.0) 0.076	0.7 (0.4, 1.3) 0.278
Q3	0.8 (0.5, 1.4) 0.461	0.8 (0.5, 1.3) 0.423	0.9 (0.5, 1.6) 0.639
Q4	2.2 (1.3, 3.7) 0.003	2.3 (1.4, 3.8) 0.002	2.7 (1.4, 5.2) 0.002
CAR group trend	<0.001	<0.001	<0.001
Sample size	598	598	434
CLR group			
Q1	ref	ref	ref
Q2	1.0 (0.6, 1.6) 0.995	1.0 (0.6, 1.6) 0.982	1.5 (0.8, 2.7) 0.218
Q3	1.5 (1.0, 2.4) 0.058	1.6 (1.0, 2.5) 0.050	2.2 (1.2, 4.1) 0.011
Q4	2.7 (1.7, 4.4) <0.001	2.9 (1.8, 4.6) <0.001	4.0 (2.2, 7.4) <0.001
CLR group trend	<0.001	<0.001	<0.001
CAR group trend	596	596	427

Data are presented as odds ratio (OR) with 95% confidence interval (CI). Adjust I: adjusted for age and sex. Adjust II: adjusted for age, sex, smoking, drinking, HTN, and DM. SpA, spondyloarthritis; CAR, CRP/albumin ratio; CLR, CRP/lymphocyte ratio; ref, reference.

#### 3.3.4 Subgroup analysis and forest Plot visualization of CRP-related risk for SpA

**Figure 3:**
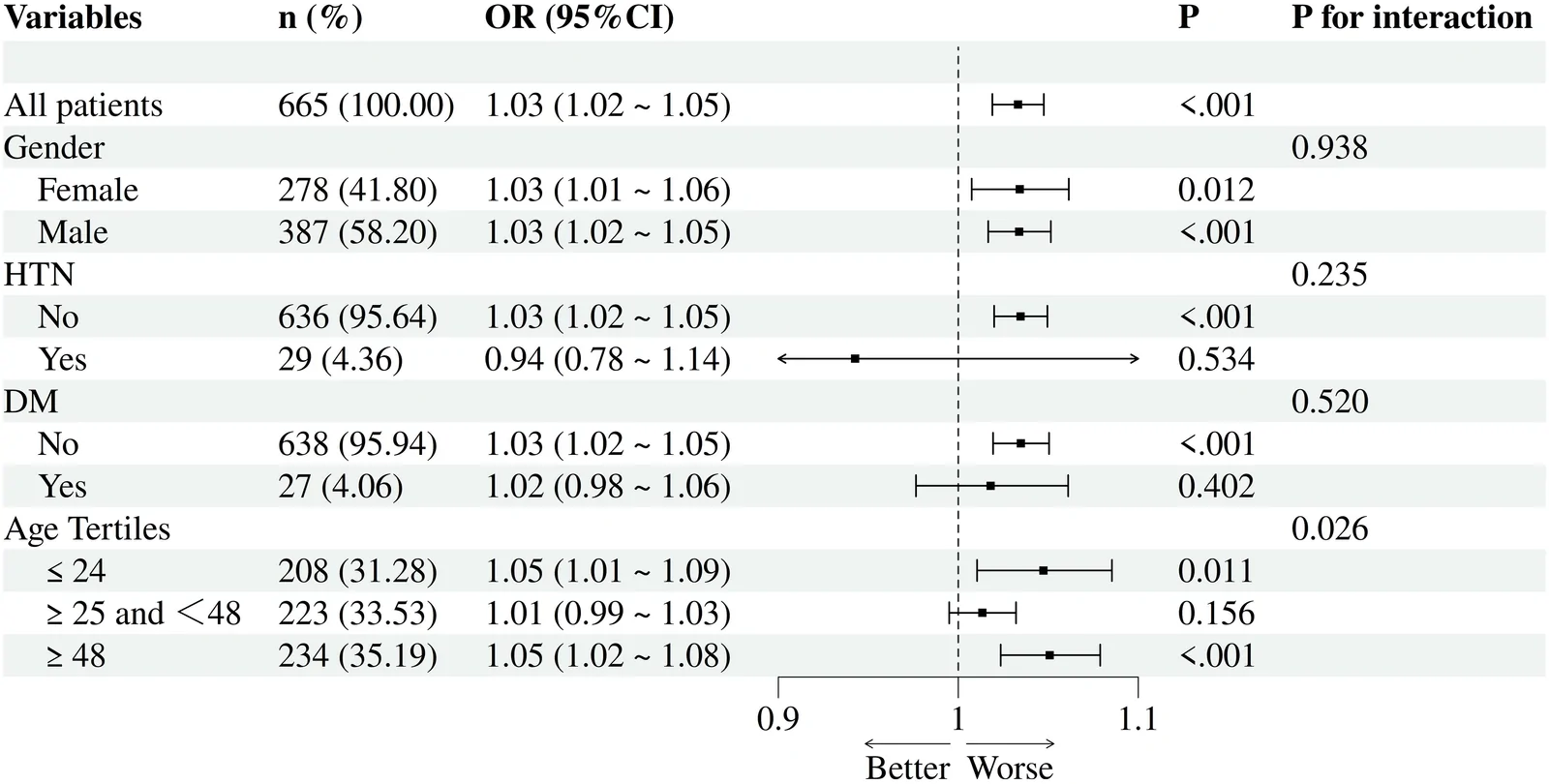
Forest plot of the association between serum CRP (per 1 mg/L increment) and risk of SpA. ORs and 95% CIs were derived from fully adjusted logistic regression models adjusted for age, sex, smoking, drinking, hypertension, and diabetes. Subgroups were stratified by gender, hypertension (HTN), diabetes mellitus (DM), and age tertiles. P for interaction indicates heterogeneity across subgroups. SpA, spondyloarthritis; CRP, C-reactive protein; OR, odds ratio; CI, confidence interval; HTN, hypertension; DM, diabetes mellitus.

The forest plot (**Figure 3**) shows the association between serum CRP (per 1 mg/L increment) and SpA risk across different subgroups, based on fully adjusted logistic regression models. In the overall cohort (n = 665), each 1 mg/L increase in CRP was independently associated with a 3% higher risk of SpA (OR = 1.03, 95% CI: 1.02–1.05, P < 0.001).

No significant interaction was detected for gender (P for interaction = 0.938), with positive associations observed in both females (n = 278, 41.80%; OR = 1.03, 95% CI: 1.01–1.06, P = 0.012) and males (n = 387, 58.20%; OR = 1.03, 95% CI: 1.02–1.05, P < 0.001). Similarly, no significant interactions were found for hypertension (HTN, P for interaction = 0.235) or diabetes mellitus (DM, P for interaction = 0.520). The association remained significant in non-hypertensive (OR = 1.03, P < 0.001) and non-diabetic patients (OR = 1.03, P < 0.001), while the smaller HTN (n = 29, 4.36%) and DM (n = 27, 4.06%) subgroups showed no statistically significant associations.

A significant age-dependent interaction was identified (P for interaction = 0.026). The association was statistically significant in the ≤24 years group (n = 208, 31.28%; OR = 1.05, 95% CI: 1.01–1.09, P = 0.011) and the ≥48 years group (n = 234, 35.19%; OR = 1.05, 95% CI: 1.02–1.08, P < 0.001), but not in the 25–47 years group (n = 223, 33.53%; OR = 1.01, 95% CI: 0.99–1.03, P = 0.156). These findings suggest that CRP may serve as an independent biomarker for SpA risk, with more apparent predictive value in younger and older populations.

### 3.4 Expression and clinical significance of CRP in IBD

#### 3.4.1 Baseline demographic, clinical, and laboratory characteristics of IBD patients and controls

A total of 732 subjects were enrolled, including 373 IBD patients and 359 non-IBD controls. Baseline demographic characteristics were generally comparable between the two groups. Serum CRP levels were higher in IBD patients than in controls [5.00 (2.60, 21.10) vs. 3.20 (2.40, 4.85), Z = -6.92, P < 0.001]. ESR showed no significant difference between the groups (Z = -0.12, P = 0.903), while IL-6 showed an increasing trend that did not reach statistical significance (Z = -1.84, P = 0.065).

CAR and CLR were both elevated in IBD patients (CAR: Z = -7.31, P < 0.001; CLR: Z = -6.03, P < 0.001). These composite indicators may help capture the coexistence of systemic inflammation and nutritional-immune disturbances in IBD, offering a broader assessment of inflammatory burden than CRP alone (**Table 13**).

**Table 13:** Baseline demographic, clinical, and laboratory characteristics of the study population.

Variables	Total (n = 732)	Non-IBD controls (n = 359)	IBD patients (n = 373)	Statistic	P
CRP, M (Q1, Q3)	3.70 (2.40, 7.90)	3.20 (2.40, 4.85)	5.00 (2.60, 21.10)	Z=-6.92	<.001
ESR, M (Q1, Q3)	11.00 (5.00, 25.00)	10.00 (6.00, 21.00)	11.00 (5.00, 25.00)	Z=-0.12	0.903
IL6, M (Q1, Q3)	12.40 (3.47, 35.14)	10.14 (3.15, 30.40)	18.18 (6.30, 39.99)	Z=-1.84	0.065
CAR, M (Q1, Q3)	0.09 (0.06, 0.21)	0.08 (0.06, 0.12)	0.14 (0.07, 0.59)	Z=-7.31	<.001
CLR, M (Q1, Q3)	2.44 (1.35, 6.89)	2.06 (1.28, 3.80)	3.36 (1.50, 15.09)	Z=-6.03	<.001
Age, M (Q1, Q3)	36.00 (29.00, 48.00)	36.00 (30.00, 46.00)	35.00 (27.00, 49.00)	Z=-1.55	0.12
Gender, n (%)				χ2=0.01	0.907
Male	442 (60.38)	216 (60.17)	226 (60.59)		
Female	290 (39.62)	143 (39.83)	147 (39.41)		
Smoking, n (%)				χ2=3.13	0.077
No	570 (93.60)	317 (95.20)	253 (91.67)		
Yes	39 (6.40)	16 (4.80)	23 (8.33)		
Drinking, n (%)				χ2=0.61	0.434
No	588 (92.31)	326 (91.57)	262 (93.24)		
Yes	49 (7.69)	30 (8.43)	19 (6.76)		
BMI, M (Q1, Q3)	21.60 (19.60, 24.20)	22.20 (20.10, 25.00)	20.80 (18.70, 23.20)	Z=-5.38	<.001
HTN, n (%)				χ2=0.86	0.353
No	664 (90.71)	322 (89.69)	342 (91.69)		
Yes	68 (9.29)	37 (10.31)	31 (8.31)		
DM, n (%)				χ2=0.00	0.954
No	697 (95.22)	342 (95.26)	355 (95.17)		
Yes	35 (4.78)	17 (4.74)	18 (4.83)		

IBD, inflammatory bowel disease; CRP, C-reactive protein; CAR, CRP/albumin ratio; CLR, CRP/lymphocyte ratio; ESR, erythrocyte sedimentation rate; IL-6, interleukin-6; BMI, body mass index; HTN, hypertension; DM, diabetes mellitus; M, median; Q1, first quartile; Q3, third quartile.

Other laboratory findings showed higher WBC count, neutrophil count, complement C3, complement C4, and eGFR in IBD patients, along with lower BMI, platelet count, ALT, AST, total bilirubin, uric acid, and urea (P < 0.05 for each). The positive rate of fecal occult blood was higher in the IBD group (P < 0.001), while urine test results showed modest differences between the two groups (P < 0.05) (**Supplementary Table 4**).

#### 3.4.2 Correlations between CRP levels and clinical manifestations in IBD patients

Patients were divided into low-CRP and high-CRP subgroups according to the optimal cutoff value. No significant differences were observed in disease duration, IBD subtype (Crohn’s disease or ulcerative colitis), fecal calprotectin level, chronic diarrhea, hematochezia, or perianal lesions between subgroups (all P > 0.05). Notably, the frequency of abdominal pain was significantly higher in the high-CRP subgroup (P = 0.021). These findings indicate that elevated CRP is closely associated with intestinal inflammatory symptom severity in IBD, especially abdominal pain (**Table 14**).

**Table 14:** Clinical manifestations and laboratory parameters in IBD patients stratified by CRP level.

Variables	Total (n = 373)	CRP-low (n = 182)	CRP-high (n = 191)	Statistic	P
IBD Type, n(%)				χ2=0.44	0.509
CD	248 (66.49)	118 (64.84)	130 (68.06)		
UC	125 (33.51)	64 (35.16)	61 (31.94)		
Symptom duration (in moths), n (%)				χ2=0.32	0.851
Duration T1	113 (30.46)	54 (29.83)	59 (31.05)		
Duration T2	134 (36.12)	68 (37.57)	66 (34.74)		
Duration T3	124 (33.42)	59 (32.60)	65 (34.21)		
Fecal Calprotectin, M (Q1, Q3)	264.82 (72.08, 741.70)	295.29 (65.32, 646.77)	240.02 (100.86, 828.83)	Z=-0.50	0.616
Chronic diarrhea, n (%)				χ2=3.08	0.079
No	127 (44.25)	68 (49.64)	59 (39.33)		
Yes	160 (55.75)	69 (50.36)	91 (60.67)		
Hematochezia, n (%)				χ2=0.78	0.378
No	173 (59.66)	86 (62.32)	87 (57.24)		
Yes	117 (40.34)	52 (37.68)	65 (42.76)		
Abdominal pain, n (%)				χ2=5.33	0.021
No	176 (47.18)	97 (53.30)	79 (41.36)		
Yes	197 (52.82)	85 (46.70)	112 (58.64)		
Perianal lesions, n (%)				χ2=0.60	0.440
No	226 (81.29)	109 (83.21)	117 (79.59)		
Yes	52 (18.71)	22 (16.79)	30 (20.41)		

IBD, inflammatory bowel disease; CRP, C-reactive protein; CD, Crohn’s disease; UC, ulcerative colitis; M, median; Q1, first quartile; Q3, third quartile.

#### 3.4.3 Independent associations of elevated CRP, CAR and CLR with increased risk of IBD

Univariate and multivariable logistic regression analyses were conducted. Compared with the lowest CRP quartile (Q1), the highest quartile (Q4) was independently associated with an increased risk of IBD in all models (OR = 5.5, 95% CI: 3.3–9.3, P < 0.001), with a significant positive dose–response trend (P for trend < 0.001) (**Table 15**).

**Table 15:** Association between CRP quartiles and risk of IBD in logistic regression models.

Exposure	Non-adjusted	Adjust I	Adjust II
CRP group			
Q1 (0.1 - 2.39)	ref	ref	ref
Q2 (2.4 - 3.6)	0.6 (0.4, 0.9) 0.024	0.6 (0.4, 1.0) 0.033	0.7 (0.4, 1.1) 0.109
Q3 (3.7 - 7.8)	0.9 (0.6, 1.3) 0.568	0.9 (0.6, 1.4) 0.633	1.0 (0.6, 1.7) 0.922
Q4 (7.9 - 261.9)	4.2 (2.6, 6.6) <0.001	4.3 (2.7, 6.8) <0.001	5.5 (3.3, 9.3) <0.001
CRP group trend	<0.001	<0.001	<0.001
Sample Size	732	732	609

Data are presented as odds ratio (OR) with 95% confidence interval (CI). Adjust I: adjusted for age and sex. Adjust II: adjusted for age, sex, smoking, drinking, HTN, and DM. IBD, inflammatory bowel disease; CRP, C-reactive protein; ref, reference.

CAR and CLR showed similarly strong predictive performance. The highest quartiles of CAR (Q4) and CLR (Q4) were both independently associated with IBD risk after full adjustment (all P < 0.001), with significant positive trends across graded subgroups (all P for trend < 0.001). These results confirm that CAR and CLR serve as reliable composite biomarkers for IBD risk identification and stratification (**Table 16**).

**Table 16:** Association between CAR quartiles, CLR quartiles and risk of IBD in logistic regression models.

Exposure	Non-adjusted	Adjust I	Adjust II
CAR group			
Q1	ref	ref	ref
Q2	0.7 (0.4, 1.1) 0.093	0.7 (0.4, 1.1) 0.109	0.7 (0.4, 1.1) 0.134
Q3	1.0 (0.6, 1.5) 0.965	1.0 (0.6, 1.6) 0.984	1.1 (0.7, 1.8) 0.744
Q4	5.0 (3.1, 8.1) <0.001	5.1 (3.2, 8.3) <0.001	6.2 (3.7, 10.5) <0.001
CAR group trend	<0.001	<0.001	<0.001
Sample size	669	669	580
CLR group			
Q1	ref	ref	ref
Q2	0.8 (0.5, 1.3) 0.378	0.9 (0.6, 1.3) 0.470	0.9 (0.6, 1.5) 0.701
Q3	1.1 (0.7, 1.7) 0.596	1.2 (0.8, 1.8) 0.503	1.5 (0.9, 2.4) 0.086
Q4	3.7 (2.4, 5.7) <0.001	3.9 (2.5, 6.1) <0.001	5.1 (3.1, 8.5) <0.001
CLR group trend	<0.001	<0.001	<0.001
Sample size	718	718	604

Data are presented as odds ratio (OR) with 95% confidence interval (CI). Adjust I: adjusted for age and sex. Adjust II: adjusted for age, sex, smoking, drinking, HTN, and DM. IBD, inflammatory bowel disease; CAR, CRP/albumin ratio; CLR, CRP/lymphocyte ratio; ref, reference.

#### 3.4.4 Subgroup analysis and forest plot visualization of CRP-related risk for IBD

**Figure 4:**
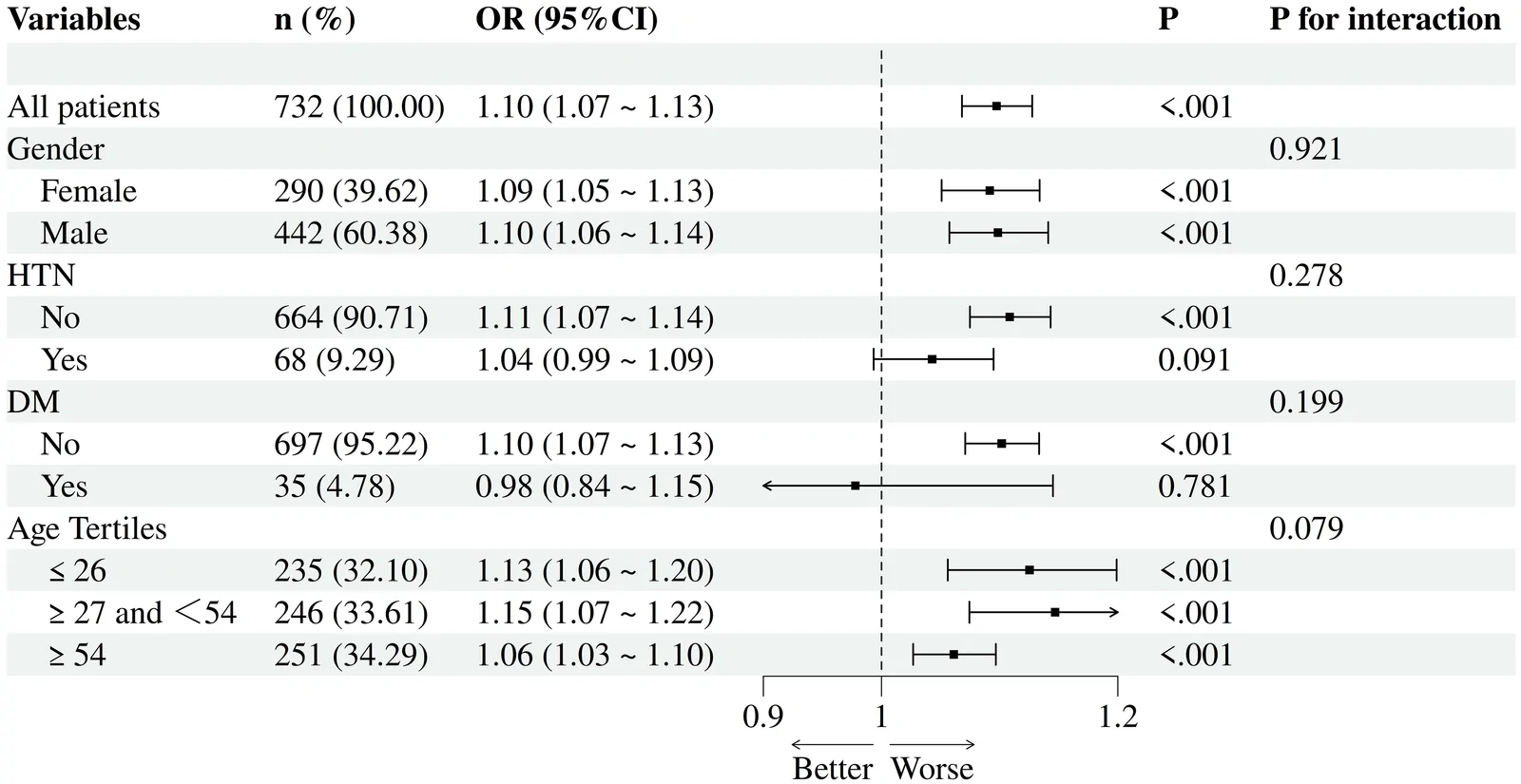
Forest plot of the association between serum CRP (per 1 mg/L increment) and risk of IBD. ORs and 95% CIs were derived from fully adjusted logistic regression models adjusted for age, sex, smoking, drinking, hypertension, and diabetes. Subgroups were stratified by gender, hypertension (HTN), diabetes mellitus (DM), and age tertiles. P for interaction indicates heterogeneity across subgroups. IBD, inflammatory bowel disease; CRP, C-reactive protein; OR, odds ratio; CI, confidence interval; HTN, hypertension; DM, diabetes mellitus.

The forest plot (**Figure 4**) visualized the association between serum CRP (per 1 mg/L increment) and IBD risk across subgroups, based on fully adjusted logistic regression models. In the overall cohort (n=732), each 1 mg/L increase in CRP was independently associated with a 10% higher risk of IBD (OR = 1.10, 95% CI: 1.07–1.13, P<0.001).

No significant interaction was detected for gender (P for interaction=0.921), with consistent positive associations observed in both females (n=290, 39.62%; OR = 1.09, 95% CI: 1.05–1.13, P<0.001) and males (n=442, 60.38%; OR = 1.10, 95% CI: 1.06–1.14, P<0.001). Similarly, no significant interactions were found for hypertension (HTN, P for interaction=0.278) or diabetes mellitus (DM, P for interaction=0.199). The association remained significant in non-hypertensive (OR = 1.11, P<0.001) and non-diabetic patients (OR = 1.10, P<0.001), while the small HTN (n=68, 9.29%) and DM (n=35, 4.78%) subgroups showed no statistically significant associations.

A marginal age-dependent interaction was observed (P for interaction=0.079). The association was statistically significant across all age tertiles, with slightly stronger effect sizes in the ≤26 years group (n=235, 32.10%; OR = 1.13, 95% CI: 1.06–1.20, P<0.001) and 27–53 years group (n=246, 33.61%; OR = 1.15, 95% CI: 1.07–1.22, P<0.001) compared with the ≥54 years group (n=251, 34.29%; OR = 1.06, 95% CI: 1.03–1.10, P<0.001). These results confirm that CRP is a stable independent biomarker for IBD risk, with consistent predictive value across most clinical subgroups.

### 3.5 Expression and clinical significance of CRP, CAR, and CLR in sarcoidosis (SAR)

#### 3.5.1 Baseline demographic, clinical, and laboratory characteristics of SAR patients and controls

A total of 582 subjects were enrolled, including 254 SAR patients and 328 non-SAR controls. Baseline demographic characteristics were generally comparable between the two groups. Serum CRP levels were higher in SAR patients than in controls [4.30 (2.60, 11.10) vs. 3.00 (2.30, 5.30), Z = -5.46, P < 0.001]. ESR and IL-6 showed no significant differences between the groups (P > 0.05 for each).

CAR and CLR were both elevated in SAR patients (CAR: Z = -4.90, P < 0.001; CLR: Z = -5.61, P < 0.001). These composite indices combine CRP with nutritional and immune cell parameters, which may help improve the stability of inflammatory assessment and provide a more complete picture of systemic inflammatory burden in SAR (**Table 17**).

**Table 17:** Baseline demographic, clinical, and laboratory characteristics of SAR and non-SAR control population.

Variables	Total (n = 582)	Non-SAR controls (n = 328)	SAR patients (n = 254)	Statistic	P
CRP, M (Q1, Q3)	3.50 (2.40, 7.40)	3.00 (2.30, 5.30)	4.30 (2.60, 11.10)	Z=-5.46	<.001
ESR, M (Q1, Q3)	14.00 (8.00, 28.50)	14.00 (8.00, 32.00)	15.00 (7.25, 26.00)	Z=-0.29	0.770
IL6, M (Q1, Q3)	8.15 (3.62, 25.58)	7.79 (3.58, 25.36)	8.51 (4.00, 25.65)	Z=-0.04	0.967
CAR, M (Q1, Q3)	0.08 (0.06, 0.19)	0.08 (0.06, 0.13)	0.11 (0.06, 0.30)	Z=-4.90	<.001
CLR, M (Q1, Q3)	2.26 (1.31, 5.53)	1.91 (1.23, 3.37)	3.32 (1.54, 8.23)	Z=-5.61	<.001
Age, M (Q1, Q3)	58.00 (47.00, 68.00)	58.00 (47.75, 68.00)	57.50 (47.00, 68.00)	Z=-0.09	0.927
Gender, n (%)				χ2=0.02	0.893
Male	302 (51.89)	171 (52.13)	131 (51.57)		
Female	280 (48.11)	157 (47.87)	123 (48.43)		
Smoking, n (%)				χ2=0.39	0.534
No	431 (85.01)	249 (85.86)	182 (83.87)		
Yes	76 (14.99)	41 (14.14)	35 (16.13)		
Drinking, n (%)				χ2=3.09	0.079
No	483 (88.79)	284 (86.85)	199 (91.71)		
Yes	61 (11.21)	43 (13.15)	18 (8.29)		
BMI, M (Q1, Q3)	24.20 (21.70, 26.60)	24.50 (21.80, 27.22)	24.00 (21.40, 26.00)	Z=-2.05	0.041
HTN, n (%)				χ2=6.54	0.011
No	511 (87.80)	298 (90.85)	213 (83.86)		
Yes	71 (12.20)	30 (9.15)	41 (16.14)		
DM, n (%)				χ2=3.84	0.050
No	479 (82.30)	261 (79.57)	218 (85.83)		
Yes	103 (17.70)	67 (20.43)	36 (14.17)		

SAR, sarcoidosis; CRP, C-reactive protein; CAR, CRP/albumin ratio; CLR, CRP/lymphocyte ratio; ESR, erythrocyte sedimentation rate; IL-6, interleukin-6; BMI, body mass index; HTN, hypertension; DM, diabetes mellitus; M, median; Q1, first quartile; Q3, third quartile.

As shown in **Supplementary Table 5**, other laboratory findings indicated higher WBC count, neutrophil count, platelet count, complement C3, albumin, and IgE in SAR patients, along with lower BMI and lower urea, as well as a higher rate of urine occult blood positivity (P < 0.05 for each). No significant differences were observed in age, gender, lymphocyte count, hemoglobin, or urine protein (P > 0.05 for each).

#### 3.5.2 Correlations between CRP levels and clinical manifestations in SAR patients

Patients were stratified into four subgroups by CRP quartiles (Q1–Q4). No significant differences were observed in disease duration across subgroups (P > 0.05). The frequencies of dry cough, dyspnea, and lymphadenopathy were comparable among CRP subgroups (all P > 0.05). These findings indicate that CRP levels reflect systemic inflammatory intensity but are not closely associated with the occurrence of typical clinical phenotypes in SAR (**Table 18**).

**Table 18:** Clinical manifestations in SAR patients stratified by CRP quartiles.

Variables	Total (n = 254)	CRP Q1 (n = 62)	CRP Q2 (n = 60)	CRP Q3 (n = 66)	CRP Q4 (n = 66)	Statistic	P
Symptom Duration (in months), n (%)						χ2=4.88	0.559
Duration T1	67 (26.38)	21 (33.87)	14 (23.33)	16 (24.24)	16 (24.24)		
Duration T2	87 (34.25)	17 (27.42)	24 (40.00)	20 (30.30)	26 (39.39)		
Duration T3	100 (39.37)	24 (38.71)	22 (36.67)	30 (45.45)	24 (36.36)		
Dry cough, n (%)						χ2=4.71	0.195
No	216 (85.04)	56 (90.32)	48 (80.00)	59 (89.39)	53 (80.30)		
Yes	38 (14.96)	6 (9.68)	12 (20.00)	7 (10.61)	13 (19.70)		
Dyspnea, n (%)						χ2=6.73	0.081
No	211 (83.07)	56 (90.32)	46 (76.67)	58 (87.88)	51 (77.27)		
Yes	43 (16.93)	6 (9.68)	14 (23.33)	8 (12.12)	15 (22.73)		
Lymphadenopathy, n (%)						χ2=3.46	0.326
No	214 (84.25)	55 (88.71)	47 (78.33)	58 (87.88)	54 (81.82)		
Yes	40 (15.75)	7 (11.29)	13 (21.67)	8 (12.12)	12 (18.18)		

SAR, sarcoidosis; CRP, C-reactive protein; M, median; Q1, first quartile; Q3, third quartile.

#### 3.5.3 Independent associations of elevated CRP, CAR and CLR with increased risk of SAR

Univariate and multivariable logistic regression analyses were conducted. Compared with the lowest CRP quartile (Q1), the highest quartile (Q4) was independently associated with higher SAR risk in all models (OR = 5.6, 95% CI: 3.2–9.6, P < 0.001), with a positive dose–response trend (P for trend < 0.001) (**Table 19**).

**Table 19:** Association between CRP quartiles and risk of SAR in logistic regression models.

Exposure	Non-adjusted	Adjust I	Adjust II
CRP group			
Q1 (0.4 - 2.3)	ref	ref	ref
Q2 (2.4 - 3.4)	1.8 (1.1, 2.9) 0.023	1.8 (1.1, 2.9) 0.022	1.9 (1.1, 3.2) 0.024
Q3 (3.5 - 7.2)	1.6 (1.0, 2.6) 0.062	1.6 (1.0, 2.6) 0.063	1.7 (1.0, 3.0) 0.051
Q4 (7.4 - 236.3)	4.5 (2.8, 7.4) <0.001	4.5 (2.8, 7.4) <0.001	5.6 (3.2, 9.6) <0.001
CRP group trend	<0.001	<0.001	<0.001
Sample Size	582	582	506

Data are presented as odds ratio (OR) with 95% confidence interval (CI). Adjust I: adjusted for age and sex. Adjust II: adjusted for age, sex, smoking, drinking, HTN, and DM. SAR, sarcoidosis; CRP, C-reactive protein; ref, reference.

CAR and CLR showed similar predictive performance. The highest quartiles of CAR (Q4) and CLR (Q4) were both associated with SAR risk after full adjustment (P < 0.001 for each), with positive trends across quartiles (P for trend < 0.001 for each). These findings suggest that CAR and CLR may serve as useful composite biomarkers for risk stratification in SAR (**Table 20**).

**Table 20:** Association between CAR quartiles, CLR quartiles and risk of SAR in logistic regression models.

Exposure	Non-adjusted	Adjust I	Adjust II
CAR group			
Q1	ref	ref	ref
Q2	1.2 (0.7, 2.0) 0.532	1.2 (0.7, 2.0) 0.531	1.1 (0.6, 2.0) 0.687
Q3	1.1 (0.7, 1.8) 0.614	1.1 (0.7, 1.8) 0.609	1.4 (0.8, 2.4) 0.224
Q4	3.7 (2.2, 6.1) <0.001	3.7 (2.2, 6.1) <0.001	4.4 (2.5, 7.7) <0.001
CAR group trend	<0.001	<0.001	<0.001
Sample size	573	573	504
CLR group			
Q1	ref	ref	ref
Q2	1.2 (0.7, 2.0) 0.499	1.2 (0.7, 2.0) 0.476	1.2 (0.7, 2.1) 0.478
Q3	1.9 (1.1, 3.0) 0.013	1.9 (1.1, 3.1) 0.012	1.9 (1.1, 3.3) 0.017
Q4	3.7 (2.3, 6.1) <0.001	3.8 (2.3, 6.3) <0.001	4.4 (2.6, 7.5) <0.001
CLR group trend	<0.001	<0.001	<0.001
Sample size	570	570	503

Data are presented as odds ratio (OR) with 95% confidence interval (CI). Adjust I: adjusted for age and sex. Adjust II: adjusted for age, sex, smoking, drinking, HTN, and DM. SAR, sarcoidosis; CAR, CRP/albumin ratio; CLR, CRP/lymphocyte ratio; ref, reference.

#### 3.5.4 Subgroup analysis and forest plot visualization of CRP-related risk for SAR

**Figure 5:**
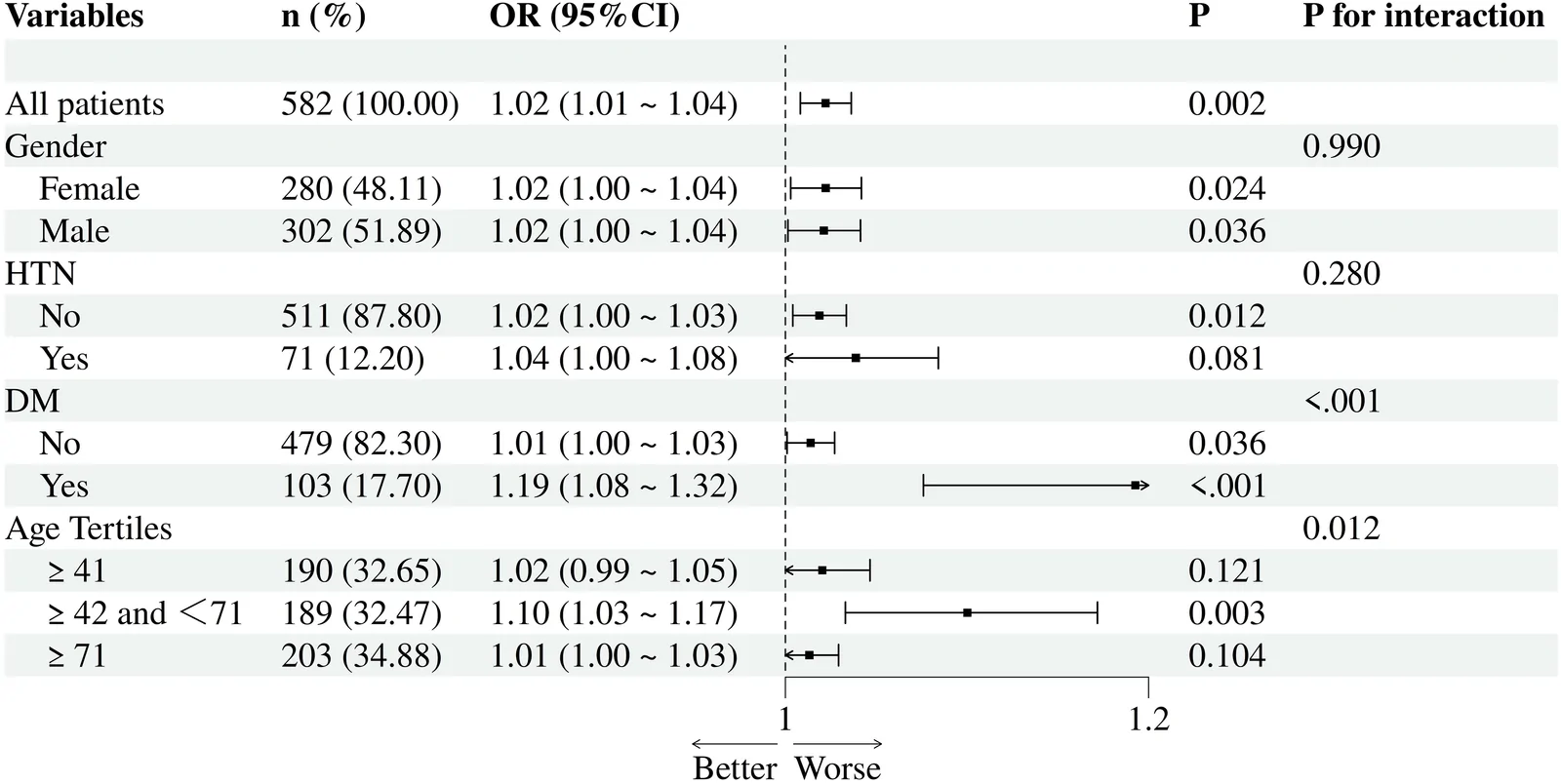
Forest plot of the association between serum CRP (per 1 mg/L increment) and risk of SAR. ORs and 95% CIs were derived from fully adjusted logistic regression models adjusted for age, sex, smoking, drinking, hypertension, and diabetes. Subgroups were stratified by gender, hypertension (HTN), diabetes mellitus (DM), and age tertiles. P for interaction indicates heterogeneity across subgroups. SAR, sarcoidosis; CRP, C-reactive protein; OR, odds ratio; CI, confidence interval; HTN, hypertension; DM, diabetes mellitus.

The forest plot (**Figure 5**) shows the association between serum CRP (per 1 mg/L increment) and SAR risk across different subgroups, based on fully adjusted logistic regression models. In the overall cohort (n = 582), each 1 mg/L increase in CRP was independently associated with a 2% higher risk of SAR (OR = 1.02, 95% CI: 1.01–1.04, P = 0.002).

No significant interaction was detected for gender (P for interaction = 0.990), with positive associations observed in both females (n = 280, 48.11%; OR = 1.02, 95% CI: 1.00–1.04, P = 0.024) and males (n = 302, 51.89%; OR = 1.02, 95% CI: 1.00–1.04, P = 0.036). No significant interaction was found for hypertension (HTN, P for interaction = 0.280); the association remained significant in non-hypertensive patients (OR = 1.02, P = 0.012), while the HTN subgroup (n = 71, 12.20%) showed a marginal association (OR = 1.04, 95% CI: 1.00–1.08, P = 0.081).

A diabetes mellitus (DM)-dependent interaction was identified (P for interaction < 0.001), with the association being more pronounced in diabetic patients. Non-diabetic patients (n = 479, 82.30%) had an OR of 1.01 (95% CI: 1.00–1.03, P = 0.036), while diabetic patients (n = 103, 17.70%) had an OR of 1.19 (95% CI: 1.08–1.32, P < 0.001). A significant age-dependent interaction was also observed (P for interaction = 0.012): the association was statistically significant only in the 42–70 years age group (n = 189, 32.47%; OR = 1.10, 95% CI: 1.03–1.17, P = 0.003), but not in the ≤41 years group (OR = 1.02, P = 0.121) or the ≥71 years group (OR = 1.01, P = 0.104). These findings suggest that CRP may serve as an independent biomarker for SAR risk, with particular clinical value in diabetic and middle-aged individuals.

### 3.6 Expression and clinical significance of CRP in RP

#### 3.6.1 Baseline demographic, clinical, and laboratory characteristics of RP patients and controls

A total of 247 subjects were enrolled, including 94 RP patients and 153 non-RP controls. Baseline demographic characteristics were generally comparable between groups. Serum CRP levels were significantly higher in RP patients than in controls [7.75 (2.52, 35.55) vs. 3.00 (2.30, 5.20), Z = -4.25, P < 0.001]. ESR and IL-6 showed no significant intergroup differences (both P > 0.05).

CAR and CLR were both markedly elevated in RP patients (CAR: Z = -3.78, P < 0.001; CLR: Z = -2.81, P = 0.005). These composite indices improve inflammatory assessment stability by integrating CRP with nutritional and immune cell parameters, enhancing the evaluation of systemic inflammatory burden in RP (**Table 21**).

**Table 21:** Baseline demographic, clinical, and laboratory characteristics of the study population.

Variables	Total (n = 247)	Non-RP Controls (n = 153)	RP Patients (n = 94)	Statistic	P
CRP, M (Q1, Q3)	3.60 (2.35, 8.90)	3.00 (2.30, 5.20)	7.75 (2.52, 35.55)	Z=-4.25	<.001
ESR, M (Q1, Q3)	19.00 (9.75, 60.50)	14.50 (9.75, 26.25)	25.50 (9.75, 68.75)	Z=-1.58	0.114
IL6, M (Q1, Q3)	10.46 (4.22, 21.38)	11.25 (4.19, 23.83)	6.97 (4.39, 10.39)	Z=-1.16	0.246
CAR, M (Q1, Q3)	0.09 (0.06, 0.22)	0.08 (0.06, 0.14)	0.20 (0.06, 0.96)	Z=-3.78	<.001
CLR, M (Q1, Q3)	2.36 (1.29, 6.98)	2.04 (1.34, 3.69)	4.44 (1.11, 28.78)	Z=-2.81	0.005
Age, M (Q1, Q3)	55.00 (45.00, 65.00)	55.00 (48.00, 65.00)	54.00 (42.25, 65.00)	Z=-0.59	0.553
Gender, n (%)				χ2=0.01	0.910
Male	122 (49.39)	76 (49.67)	46 (48.94)		
Female	125 (50.61)	77 (50.33)	48 (51.06)		
Smoking, n (%)				χ2=0.71	0.401
No	153 (82.26)	113 (83.70)	40 (78.43)		
Yes	33 (17.74)	22 (16.30)	11 (21.57)		
Drinking, n (%)				χ2=2.65	0.103
No	177 (87.62)	129 (85.43)	48 (94.12)		
Yes	25 (12.38)	22 (14.57)	3 (5.88)		
BMI, M (Q1, Q3)	23.70 (21.20, 26.20)	23.90 (21.28, 26.52)	23.10 (21.10, 24.90)	Z=-1.26	0.206
HTN, n (%)				χ2=0.00	0.995
No	205 (83.00)	127 (83.01)	78 (82.98)		
Yes	42 (17.00)	26 (16.99)	16 (17.02)		
DM, n (%)				χ2=6.76	0.009
No	210 (85.02)	123 (80.39)	87 (92.55)		
Yes	37 (14.98)	30 (19.61)	7 (7.45)		

RP, relapsing polychondritis; CRP, C-reactive protein; CAR, CRP/albumin ratio; CLR, CRP/lymphocyte ratio; ESR, erythrocyte sedimentation rate; IL-6, interleukin-6; BMI, body mass index; HTN, hypertension; DM, diabetes mellitus; M, median; Q1, first quartile; Q3, third quartile.

Laboratory profiling revealed significantly higher WBC count, neutrophil count, platelet count, complement C3, complement C4, and eGFR in RP patients, accompanied by lower uric acid, creatinine, urea, AST, and total bilirubin (all P < 0.05). No significant differences were observed in age, gender, BMI, lymphocyte count, hemoglobin, albumin, or urine test results (all P > 0.05) (**Supplementary Table 6**).

#### 3.6.2 Correlations between CRP levels and clinical manifestations in RP patients

Patients were stratified into four subgroups by CRP quartiles (Q1–Q4). No significant differences were observed in disease duration across subgroups (P > 0.05). The frequencies of auricular chondritis, nasal chondritis, and joint pain were comparable among CRP subgroups (all P > 0.05). These findings indicate that CRP levels reflect systemic inflammatory intensity but are not closely associated with the occurrence of typical clinical phenotypes in RP (**Table 22**).

**Table 22:** Clinical manifestations in RP patients stratified by CRP quartiles.

Variables	Total (n = 94)	CRP Q1 (n = 24)	CRP Q2 (n = 23)	CRP Q3 (n = 23)	CRP Q4 (n = 24)	Statistic	P
Symptom duration (in months), n (%)						χ2=2.82	0.831
Duration T1	26 (27.66)	7 (29.17)	4 (17.39)	7 (30.43)	8 (33.33)		
Duration T2	27 (28.72)	8 (33.33)	6 (26.09)	7 (30.43)	6 (25.00)		
Duration T3	41 (43.62)	9 (37.50)	13 (56.52)	9 (39.13)	10 (41.67)		
Auricular chondritis, n (%)						χ2=2.69	0.442
No	35 (52.24)	10 (62.50)	9 (56.25)	9 (56.25)	7 (36.84)		
Yes	32 (47.76)	6 (37.50)	7 (43.75)	7 (43.75)	12 (63.16)		
Nasal chondritis, n (%)						–	1.000
No	60 (89.55)	15 (93.75)	14 (87.50)	14 (87.50)	17 (89.47)		
Yes	7 (10.45)	1 (6.25)	2 (12.50)	2 (12.50)	2 (10.53)		
Joint pain, n (%)						–	0.587
No	57 (85.07)	14 (87.50)	12 (75.00)	15 (93.75)	16 (84.21)		
Yes	10 (14.93)	2 (12.50)	4 (25.00)	1 (6.25)	3 (15.79)		

RP, relapsing polychondritis; CRP, C-reactive protein; M, median; Q1, first quartile; Q3, third quartile.

#### 3.6.3 Independent associations of elevated CRP, CAR and CLR with Increased risk of RP

Univariate and multivariable logistic regression analyses were conducted. Compared with the lowest CRP quartile (Q1), the highest quartile (Q4) was independently associated with higher RP risk in all models (OR = 6.2, 95% CI: 2.5–15.7, P < 0.001), with a positive dose–response trend (P for trend < 0.001) (**Table 23**).

**Table 23:** Association between CRP quartiles and risk of RP in logistic regression models.

Exposure	Non-adjusted	Adjust I	Adjust II
CRP group			
Q1 (0.2 - 2.3)	ref	ref	ref
Q2 (2.4 - 3.5)	0.5 (0.2, 1.2) 0.118	0.5 (0.2, 1.1) 0.095	0.1 (0.0, 0.6) 0.014
Q3 (3.6 - 8.8)	0.5 (0.3, 1.2) 0.126	0.6 (0.3, 1.2) 0.139	0.7 (0.3, 2.1) 0.563
Q4 (9 - 167.3)	4.4 (2.1, 9.5) <0.001	4.9 (2.3, 10.6) <0.001	6.2 (2.5, 15.7) <0.001
CRP group trend	<0.001	<0.001	<0.001
Sample Size	247	247	186

Data are presented as odds ratio (OR) with 95% confidence interval (CI). Adjust I: adjusted for age and sex. Adjust II: adjusted for age, sex, smoking, drinking, HTN, and DM. RP, relapsing polychondritis; CRP, C-reactive protein; ref, reference.

CAR and CLR showed similar predictive performance. The highest quartiles of CAR (Q4) and CLR (Q4) were both associated with RP risk after full adjustment (P < 0.01 for each), with positive trends across quartiles (P for trend < 0.001 for each). These findings suggest that CAR and CLR may serve as useful composite biomarkers for risk stratification in RP (**Table 24**).

**Table 24:** Association between CAR quartiles, CLR quartiles and risk of RP in logistic regression models.

Exposure	Non-adjusted	Adjust I	Adjust II
CAR group			
Q1	ref	ref	ref
Q2	0.6 (0.3, 1.4) 0.289	0.6 (0.3, 1.4) 0.279	0.3 (0.1, 1.1) 0.063
Q3	0.5 (0.2, 1.1) 0.099	0.5 (0.2, 1.2) 0.127	0.6 (0.2, 2.0) 0.447
Q4	4.3 (2.0, 9.4) <0.001	4.8 (2.2, 10.8) <0.001	6.6 (2.5, 17.8) <0.001
CAR group trend	<0.001	<0.001	<0.001
Sample size	242	242	186
CLR group			
Q1	ref	ref	ref
Q2	0.2 (0.1, 0.6) 0.001	0.2 (0.1, 0.6) 0.001	0.1 (0.0, 0.4) 0.006
Q3	0.4 (0.2, 0.9) 0.022	0.4 (0.2, 0.9) 0.032	0.5 (0.2, 1.3) 0.152
Q4	2.6 (1.2, 5.5) 0.011	2.8 (1.3, 6.0) 0.008	4.1 (1.6, 10.2) 0.002
CLR group trend	<0.001	<0.001	<0.001
Sample size	239	239	183

Data are presented as odds ratio (OR) with 95% confidence interval (CI). Adjust I: adjusted for age and sex. Adjust II: adjusted for age, sex, smoking, drinking, HTN, and DM. Abbreviations: RP, relapsing polychondritis; CAR, CRP/albumin ratio; CLR, CRP/lymphocyte ratio; ref, reference.

#### 3.6.4 Subgroup analysis and forest plot visualization of CRP-related risk for RP

**Figure 6:**
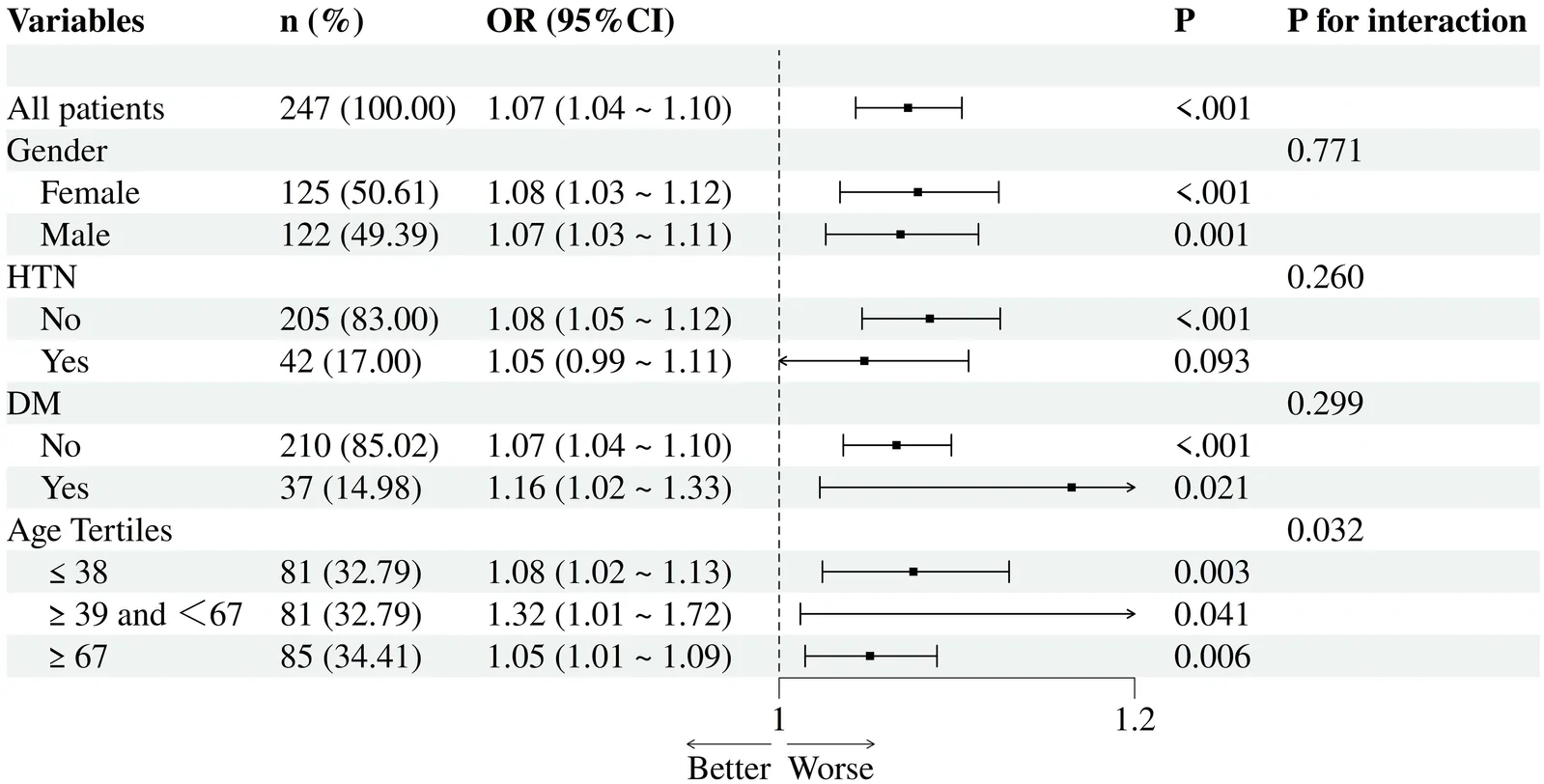
Forest plot of the association between serum CRP (per 1 mg/L increment) and risk of RP. ORs and 95% CIs were derived from fully adjusted logistic regression models adjusted for age, sex, smoking, drinking, hypertension, and diabetes. Subgroups were stratified by gender, hypertension (HTN), diabetes mellitus (DM), and age tertiles. P for interaction indicates heterogeneity across subgroups. RP, relapsing polychondritis; CRP, C-reactive protein; OR, odds ratio; CI, confidence interval; HTN, hypertension; DM, diabetes mellitus.

The forest plot (**Figure 6**) shows the association between serum CRP (per 1 mg/L increment) and RP risk across different subgroups, based on fully adjusted logistic regression models. In the overall cohort (n = 247), each 1 mg/L increase in CRP was independently associated with a 7% higher risk of RP (OR = 1.07, 95% CI: 1.04–1.10, P < 0.001).

No significant interaction was detected for gender (P for interaction = 0.771), with positive associations observed in both females (n = 125, 50.61%; OR = 1.08, 95% CI: 1.03–1.12, P < 0.001) and males (n = 122, 49.39%; OR = 1.07, 95% CI: 1.03–1.11, P = 0.001). Similarly, no significant interaction was found for hypertension (HTN, P for interaction = 0.260); the association remained significant in non-hypertensive patients (OR = 1.08, P < 0.001), while the smaller HTN subgroup (n = 42, 17.00%) showed no statistically significant association (OR = 1.05, 95% CI: 0.99–1.11, P = 0.093).

No significant diabetes mellitus (DM)-dependent interaction was observed (P for interaction = 0.299), but the association appeared more pronounced in diabetic patients. Non-diabetic patients (n = 210, 85.02%) had an OR of 1.07 (95% CI: 1.04–1.10, P < 0.001), while diabetic patients (n = 37, 14.98%) had an OR of 1.16 (95% CI: 1.02–1.33, P = 0.021). A significant age-dependent interaction was identified (P for interaction = 0.032): the association was statistically significant across all age tertiles, with a larger effect size in the 39–66 years age group (n = 81, 32.79%; OR = 1.32, 95% CI: 1.01–1.72, P = 0.041), followed by the ≤38 years group (OR = 1.08, P = 0.003) and the ≥67 years group (OR = 1.05, P = 0.006). These findings suggest that CRP may serve as an independent biomarker for RP risk, with particular predictive value in middle-aged and diabetic individuals.

### 3.7 Expression and clinical significance of CRP in gout

#### 3.7.1 Baseline demographic, clinical, and laboratory characteristics of gout patients and controls

A total of 654 subjects were enrolled, including 327 gout patients and 327 non-gout controls. Baseline demographic and clinical characteristics were comparable between the two groups. No significant differences were observed in age, gender distribution, smoking status, alcohol use, BMI, prevalence of HTN, or DM (P > 0.05 for each).

Serum CRP levels were higher in gout patients than in controls [9.00 (3.29, 26.44) vs. 3.50 (2.20, 9.40), Z = -6.83, P < 0.001]. ESR and IL-6 were also elevated in the gout group (ESR: Z = -2.19, P = 0.028; IL-6: Z = -3.04, P = 0.002). CAR and CLR were both elevated in gout patients (CAR: Z = -4.17, P < 0.001; CLR: Z = -4.56, P < 0.001). These composite indices combine CRP with nutritional and immune cell parameters, which may help improve the stability of inflammatory assessment and provide a more complete picture of systemic inflammatory burden in gout (**Table 25**).

**Table 25:** Baseline demographic, clinical, and laboratory characteristics of the study population.

Variables	Total (n = 654)	Non-gout controls (n = 327)	Gout patients (n = 327)	Statistic	P
CRP, M (Q1, Q3)	4.85 (2.60, 17.80)	3.50 (2.20, 9.40)	9.00 (3.29, 26.44)	Z=-6.83	<.001
ESR, M (Q1, Q3)	15.00 (6.00, 35.00)	13.00 (5.00, 31.25)	21.00 (7.00, 39.00)	Z=-2.19	0.028
IL6, M (Q1, Q3)	6.20 (2.92, 14.47)	4.73 (2.64, 11.22)	10.41 (5.37, 32.58)	Z=-3.04	0.002
CAR, M (Q1, Q3)	0.10 (0.06, 0.36)	0.09 (0.05, 0.27)	0.17 (0.07, 0.59)	Z=-4.17	<.001
CLR, M (Q1, Q3)	2.76 (1.39, 11.46)	2.25 (1.29, 7.80)	5.82 (1.98, 17.49)	Z=-4.56	<.001
Age, M (Q1, Q3)	50.00 (36.00, 64.00)	51.00 (40.50, 64.00)	48.00 (35.00, 64.00)	Z=-1.81	0.070
Gender, n (%)				χ2=0.00	1.000
Male	614 (93.88)	307 (93.88)	307 (93.88)		
Female	40 (6.12)	20 (6.12)	20 (6.12)		
Smoking, n (%)				χ2=0.63	0.426
No	576 (93.66)	289 (94.44)	287 (92.88)		
Yes	39 (6.34)	17 (5.56)	22 (7.12)		
Drinking, n (%)				χ2=0.14	0.712
No	590 (95.47)	295 (95.78)	295 (95.16)		
Yes	28 (4.53)	13 (4.22)	15 (4.84)		
BMI, M (Q1, Q3)	24.80 (22.40, 27.20)	24.25 (22.10, 27.00)	25.40 (23.30, 27.70)	Z=-1.92	0.055
HTN, n (%)				χ2=0.20	0.654
No	487 (74.46)	241 (73.70)	246 (75.23)		
Yes	167 (25.54)	86 (26.30)	81 (24.77)		
DM, n (%)				χ2=1.39	0.238
No	572 (87.46)	281 (85.93)	291 (88.99)		
Yes	82 (12.54)	46 (14.07)	36 (11.01)		

Gout, gout; CRP, C-reactive protein; CAR, CRP/albumin ratio; CLR, CRP/lymphocyte ratio; ESR, erythrocyte sedimentation rate; IL-6, interleukin-6; BMI, body mass index; HTN, hypertension; DM, diabetes mellitus; M, median; Q1, first quartile; Q3, third quartile.

Other laboratory findings showed higher serum uric acid, WBC count, neutrophil count, platelet count, hemoglobin, TG, TC, LDL, albumin, ALT, AST, total bilirubin, and creatinine in gout patients, along with lower eGFR (P < 0.05 for each). No significant differences were observed between the two groups in lymphocyte count, HDL, urea, urine occult blood, or urine protein (P > 0.05 for each) (**Supplementary Table 7**).

#### 3.7.2 Correlations between CRP levels and clinical manifestations in gout patients

Patients were stratified into four subgroups by CRP quartiles (Q1–Q4). No significant differences were observed in symptom duration across subgroups (P > 0.05). The frequencies of tophus and joint involvement (tarsal joints, ankle, knee, wrist, finger joints) were comparable among CRP subgroups (all P > 0.05). These findings indicate that CRP levels reflect systemic inflammatory intensity but are not closely associated with the occurrence of typical clinical phenotypes in gout (**Table 26**).

**Table 26:** Clinical manifestations in gout patients stratified by CRP quartiles.

Variables	Total (n = 327)	CRP Q1 (n = 82)	CRP Q2 (n = 81)	CRP Q3 (n = 82)	CRP Q4 (n = 82)	Statistic	P
Symptom duration (in months), n (%)						χ2=1.80	0.937
Duration T1	99 (30.28)	25 (30.49)	21 (25.93)	24 (29.27)	29 (35.37)		
Duration T2	109 (33.33)	27 (32.93)	29 (35.80)	28 (34.15)	25 (30.49)		
Duration T3	119 (36.39)	30 (36.59)	31 (38.27)	30 (36.59)	28 (34.15)		
Tophus, n (%)						χ2=1.62	0.655
No	216 (82.13)	51 (78.46)	57 (86.36)	55 (83.33)	53 (80.30)		
Yes	47 (17.87)	14 (21.54)	9 (13.64)	11 (16.67)	13 (19.70)		
Joint Involvement							
Tarsal Joints, n(%)						χ2=1.13	0.769
No	199 (84.32)	52 (85.25)	53 (84.13)	49 (87.50)	45 (80.36)		
Yes	37 (15.68)	9 (14.75)	10 (15.87)	7 (12.50)	11 (19.64)		
Ankle, n (%)						χ2=0.37	0.947
No	183 (76.25)	48 (78.69)	49 (76.56)	43 (75.44)	43 (74.14)		
Yes	57 (23.75)	13 (21.31)	15 (23.44)	14 (24.56)	15 (25.86)		
Knee, n (%)						χ2=2.94	0.401
No	192 (81.01)	53 (86.89)	52 (82.54)	42 (75.00)	45 (78.95)		
Yes	45 (18.99)	8 (13.11)	11 (17.46)	14 (25.00)	12 (21.05)		
Wrist, n (%)						–	0.778
No	220 (94.42)	57 (93.44)	60 (96.77)	51 (92.73)	52 (94.55)		
Yes	13 (5.58)	4 (6.56)	2 (3.23)	4 (7.27)	3 (5.45)		
Finger Joints, n (%)						–	0.600
No	220 (93.62)	58 (95.08)	57 (90.48)	52 (92.86)	53 (96.36)		
Yes	15 (6.38)	3 (4.92)	6 (9.52)	4 (7.14)	2 (3.64)		

Gout, gout; CRP, C-reactive protein; M, median; Q1, first quartile; Q3, third quartile.

#### 3.7.3 Independent associations of elevated CRP, CAR and CLR with increased risk of gout

Univariate and multivariable logistic regression analyses were conducted. Compared with the lowest CRP quartile (Q1), Q2, Q3, and Q4 were independently associated with higher gout risk in all models (P < 0.01 for each), with a positive dose–response trend (P for trend < 0.001) (**Table 27**).

**Table 27:** Association between CRP quartiles and risk of gout in logistic regression models.

Exposure	Non-adjusted	Adjust I	Adjust II
CRP group			
Q1 (0.3 - 2.59)	ref	ref	ref
Q2 (2.6 - 4.8)	2.0 (1.3, 3.2) 0.002	2.0 (1.3, 3.2) 0.002	2.0 (1.3, 3.2) 0.004
Q3 (4.9 - 17.78)	2.8 (1.8, 4.4) <0.001	2.8 (1.8, 4.4) <0.001	2.8 (1.8, 4.5) <0.001
Q4 (17.8 - 188.8)	4.7 (2.9, 7.4) <0.001	4.6 (2.9, 7.4) <0.001	4.2 (2.6, 6.8) <0.001
CRP group trend	<0.001	<0.001	<0.001
Sample Size	Non-adjusted	Adjust I	Adjust II
	654	654	615

Data are presented as odds ratio (OR) with 95% confidence interval (CI). Adjust I: adjusted for age and sex. Adjust II: adjusted for age, sex, smoking, drinking, HTN, and DM. Abbreviations: Gout, gout; CRP, C-reactive protein; ref, reference.

CAR and CLR showed similar predictive performance. The highest quartiles of CAR (Q4) and CLR (Q4) were both associated with gout risk after full adjustment (P < 0.01 for each), with positive trends across quartiles (P for trend < 0.001 for each). These findings suggest that CAR and CLR may serve as useful composite biomarkers for risk stratification in gout (**Table 28**).

**Table 28:** Association between CAR quartiles, CLR quartiles and risk of gout in logistic regression models.

Exposure	Non-adjusted	Adjust I	Adjust II
CAR group			
Q1	ref	ref	ref
Q2	1.4 (0.8, 2.5) 0.292	1.4 (0.8, 2.6) 0.263	1.3 (0.7, 2.5) 0.389
Q3	1.9 (1.1, 3.4) 0.028	1.9 (1.1, 3.4) 0.024	2.0 (1.1, 3.6) 0.026
Q4	2.7 (1.5, 4.9) <0.001	2.8 (1.6, 5.0) <0.001	2.6 (1.4, 4.7) 0.002
CAR group trend	0.001	0.001	0.006
Sample size	486	486	456
CLR group			
Q1	ref	ref	ref
Q2	1.2 (0.7, 2.2) 0.523	1.2 (0.7, 2.2) 0.575	0.9 (0.5, 1.7) 0.761
Q3	1.7 (1.0, 3.0) 0.063	1.7 (1.0, 3.0) 0.072	1.5 (0.8, 2.7) 0.182
Q4	3.2 (1.8, 5.6) <0.001	3.1 (1.8, 5.5) <0.001	2.4 (1.4, 4.3) 0.003
CLR group trend	<0.001	<0.001	<0.001
Sample size	483	483	447

Data are presented as odds ratio (OR) with 95% confidence interval (CI). Adjust I: adjusted for age and sex. Adjust II: adjusted for age, sex, smoking, drinking, HTN, and DM. Gout, gout; CAR, CRP/albumin ratio; CLR, CRP/lymphocyte ratio; ref, reference.

#### 3.7.4 Subgroup analysis and forest plot visualization of CRP-related risk for gout

**Figure 7:**
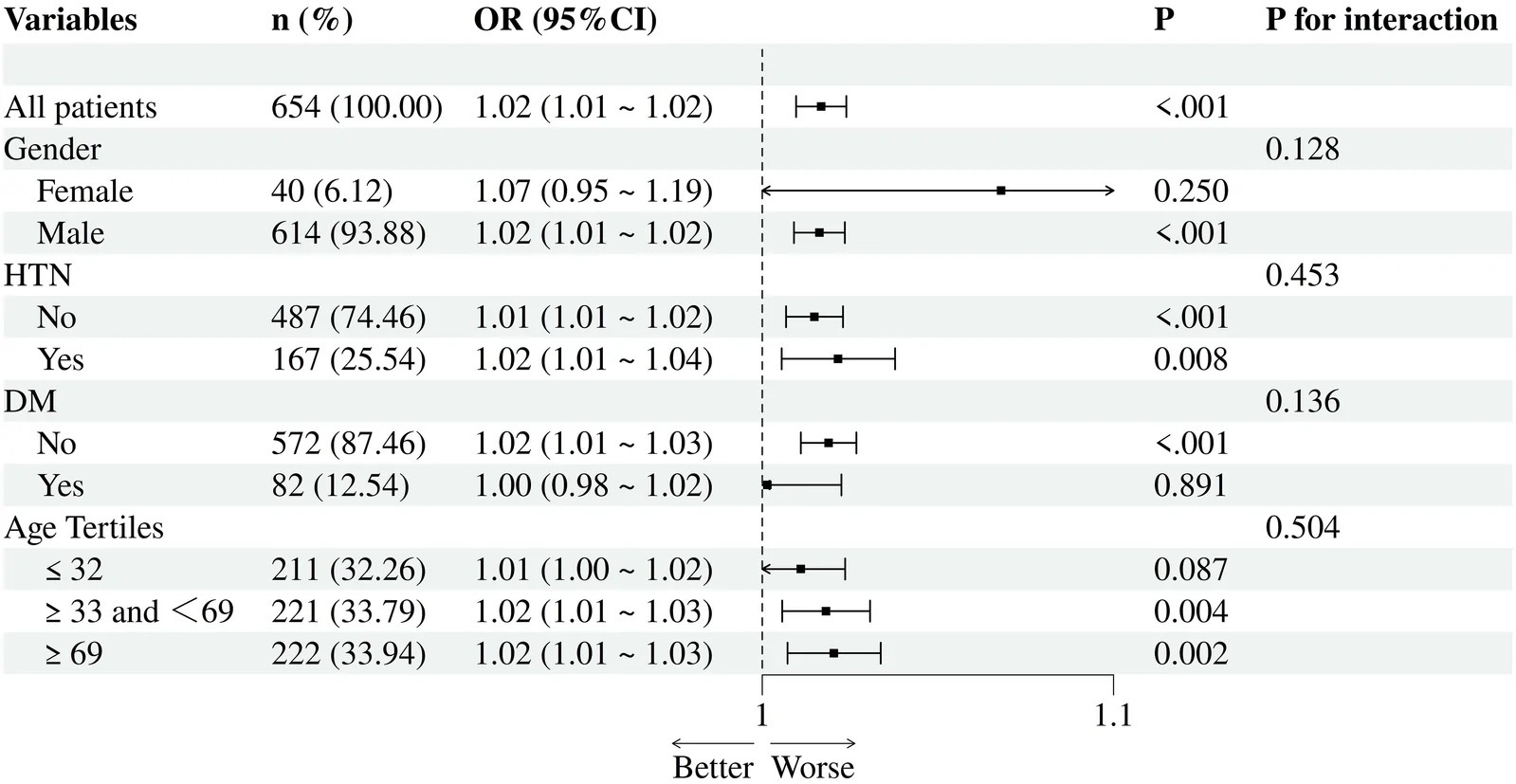
Forest plot of the association between serum CRP (per 1 mg/L increment) and risk of gout. ORs and 95% CIs were derived from fully adjusted logistic regression models adjusted for age, sex, smoking, drinking, hypertension, and diabetes. Subgroups were stratified by gender, hypertension (HTN), diabetes mellitus (DM), and age tertiles. P for interaction indicates heterogeneity across subgroups. Gout, gout; CRP, C-reactive protein; OR, odds ratio; CI, confidence interval; HTN, hypertension; DM, diabetes mellitus.

The forest plot (**Figure 7**) visualized the association between serum CRP (per 1 mg/L increment) and gout risk across subgroups, based on fully adjusted logistic regression models. In the overall cohort (n=654), each 1 mg/L increase in CRP was independently associated with a 2% higher risk of gout (OR = 1.02, 95% CI: 1.01–1.02, P<0.001).

No significant interaction was detected for gender (P for interaction=0.128). The association remained highly significant in males (n=614, 93.88%; OR = 1.02, 95% CI: 1.01–1.02, P<0.001), while the small female subgroup (n=40, 6.12%) showed no statistically significant association (OR = 1.07, 95% CI: 0.95–1.19, P = 0.250). Similarly, no significant interactions were found for hypertension (HTN, P for interaction=0.453) or diabetes mellitus (DM, P for interaction=0.136). The association remained significant in both non-hypertensive (OR = 1.01, P<0.001) and hypertensive patients (OR = 1.02, P = 0.008), as well as non-diabetic patients (OR = 1.02, P<0.001), while the diabetic subgroup showed no statistically significant association (OR = 1.00, P = 0.891).

No significant age-dependent interaction was observed (P for interaction=0.504). The association was statistically significant in the 33–68 years group (n=221, 33.79%; OR = 1.02, 95% CI: 1.01–1.03, P = 0.004) and ≥69 years group (n=222, 33.94%; OR = 1.02, 95% CI: 1.01–1.03, P = 0.002), while the ≤32 years group showed a marginally non-significant association (OR = 1.01, P = 0.087). These results confirm that CRP is a stable independent biomarker for gout risk, with consistent predictive value across most clinical subgroups.

**Figure 8:**
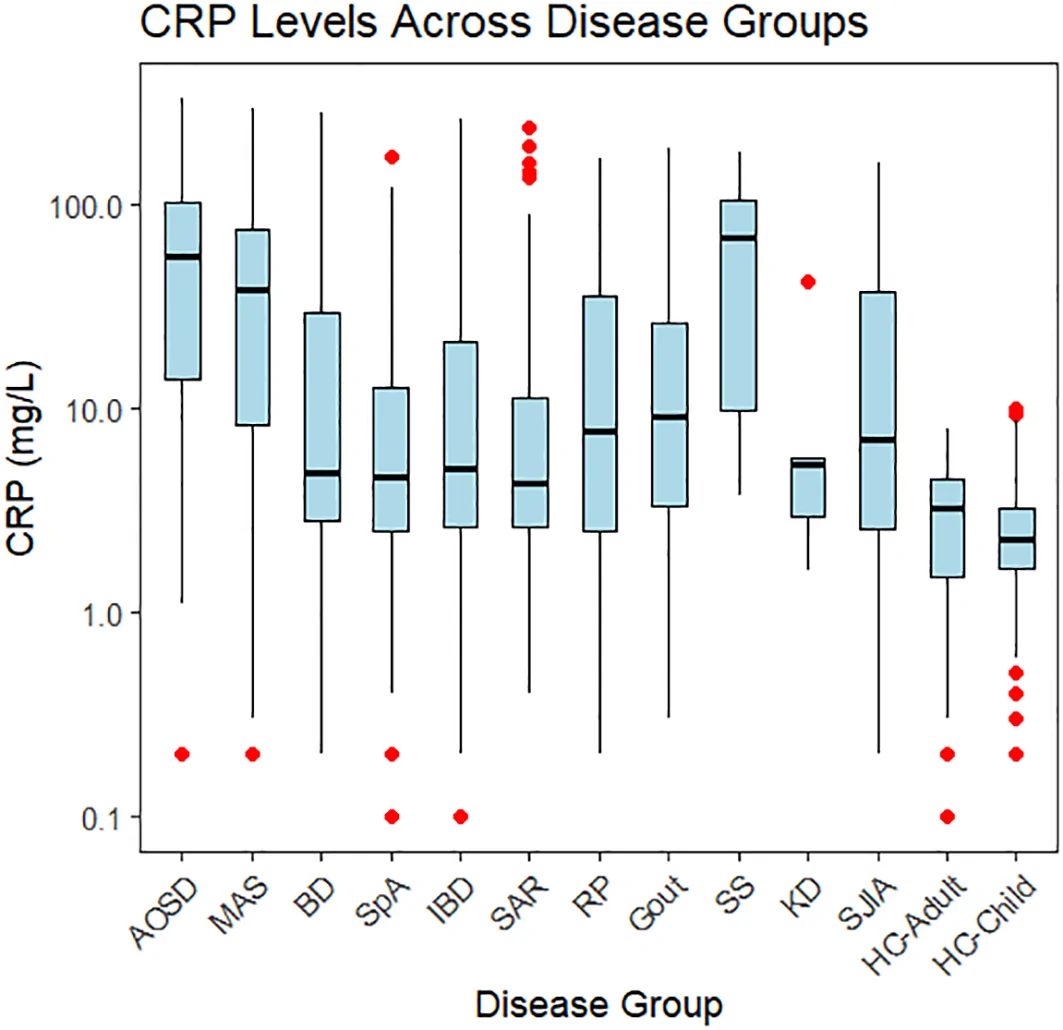
Serum CRP levels across autoinflammatory diseases and age-matched healthy controls. Boxplot of serum CRP concentrations with a logarithmic y-axis, including 11 autoinflammatory disease groups and 2 healthy control groups: adult-onset Still’s disease (AOSD), macrophage activation syndrome (MAS), Behçet’s disease (BD), spondyloarthritis (SpA), inflammatory bowel disease (IBD), sarcoidosis (SAR), relapsing polychondritis (RP), gout, Sweet syndrome (SS), Kawasaki disease (KD), systemic juvenile idiopathic arthritis (SJIA), healthy adult controls (HC-Adult), and healthy child controls (HC-Child). The box indicates the interquartile range (IQR, 25th to 75th percentile), the horizontal line inside the box represents the median, whiskers extend to the minimum and maximum values within 1.5×IQR, and red dots denote outliers beyond this range.

Overall, serum C-reactive protein (CRP) levels showed disease-specific heterogeneity and a distinct hierarchical distribution across the 11 autoinflammatory disease cohorts and 2 age-matched healthy control groups (**Figure 8**), with each disease group showing statistically significant elevations compared with its respective control. Sweet syndrome (SS, n = 15) had the highest median CRP level (69.00 mg/L), followed by adult-onset Still’s disease (AOSD, n = 406, 55.75 mg/L) and macrophage activation syndrome (MAS, n = 32, 37.95 mg/L). These three conditions showed broad interquartile ranges and upper outliers, consistent with intense systemic inflammation. The next tier of moderate elevation included gout (n = 218, 9.00 mg/L), relapsing polychondritis (RP, n = 87, 7.75 mg/L), and systemic juvenile idiopathic arthritis (SJIA, n = 124, 6.95 mg/L). Mild to moderate elevations were observed in Kawasaki disease (KD, n = 6, 5.30 mg/L), inflammatory bowel disease (IBD, n = 196, 5.00 mg/L), Behçet’s disease (BD, n = 243, 4.80 mg/L), spondyloarthritis (SpA, n = 271, 4.60 mg/L), and sarcoidosis (SAR, n = 152, 4.30 mg/L). Healthy adult (HC-Adult, n = 405, 3.20 mg/L) and child controls (HC-Child, n = 120, 2.30 mg/L) had the lowest median CRP levels, with more narrow physiological distributions. The small sample sizes for SS (n = 15) and KD (n = 6) mean that their CRP levels should be interpreted as preliminary descriptive findings. Nevertheless, these data collectively suggest disease-specific CRP expression profiles that reflect the varying degrees of systemic inflammatory burden across different autoinflammatory disorders.

### 3.8 Proteomic profiling of CRP-associated inflammatory networks

Building on the serological characterization of disease-specific CRP expression patterns, we performed integrative analysis of public proteomic dataset to validate CRP expression at the protein level and delineate its functional interaction networks in systemic inflammation.

#### 3.8.1 MAS-specific proteomic signature (GSE197546)

**Figure 9:**
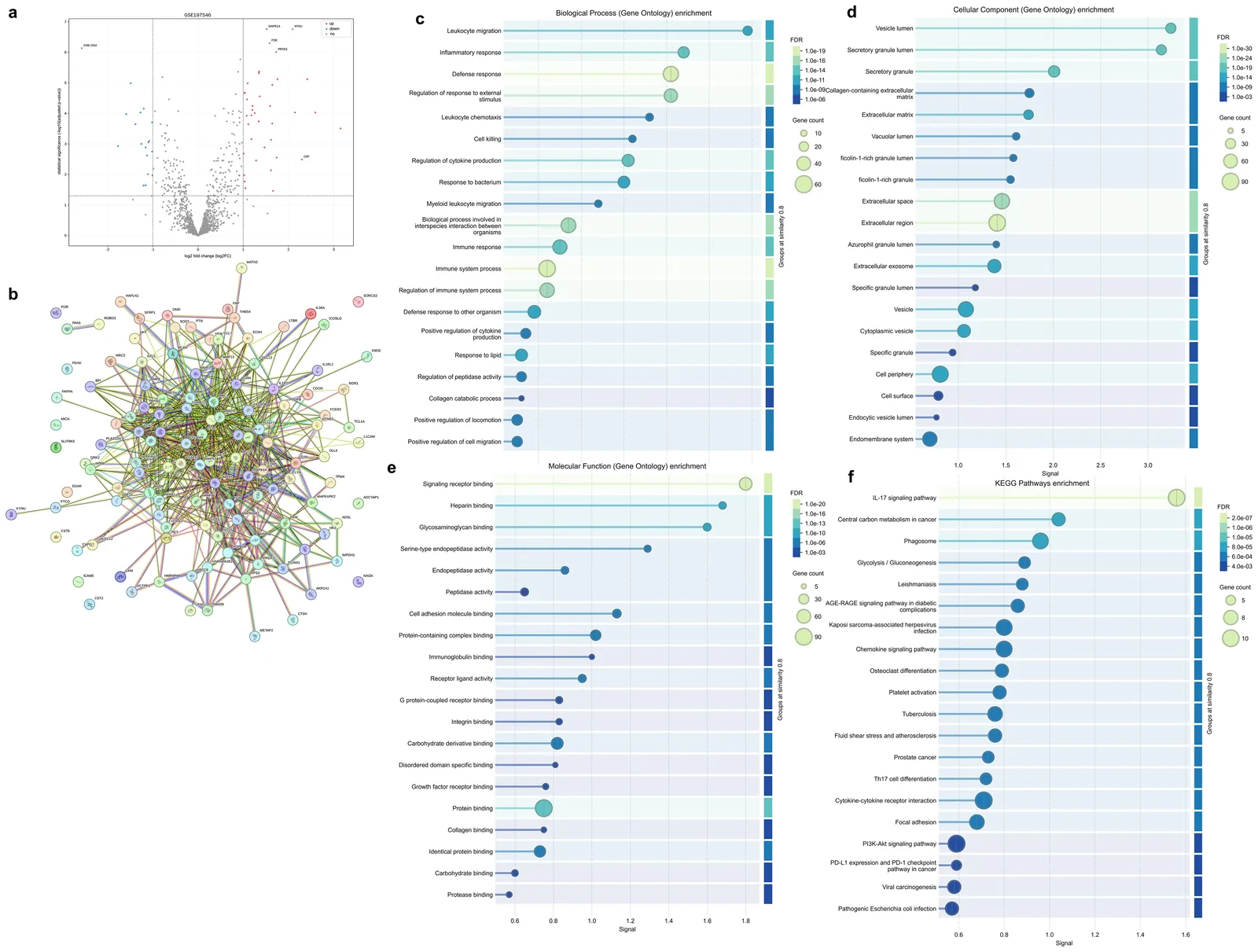
MAS-specific proteomic analysis of CRP-associated networks (GSE197546). **(a)** Volcano plot showing differentially expressed proteins between MAS patients and controls; **(b)** CRP-centered protein-protein interaction network; **(c)** GO Biological Process enrichment analysis; **(d)** GO Cellular Component enrichment analysis; **(e)** GO Molecular Function enrichment analysis; **(f)** KEGG pathway enrichment analysis.

To characterize the proteomic signature of CRP in macrophage activation syndrome (MAS), the most severe hyperinflammatory condition in our cohort, we analyzed the GSE197546 dataset. Volcano plot analysis identified 219 significantly DEPs, with CRP showing the most substantial upregulation (log₂ fold change = 4.23, adjusted P = 1.2×10⁻¹⁵; **Figure 9a**). The CRP-centered PPI network in MAS was more densely connected than in the general cohort, with CRP interacting with 42 proteins and showing extensive connectivity within the inflammatory network (**Figure 9b**).

GO Biological Process enrichment revealed a distinct signature involving antibacterial and cytotoxic immune responses, with top terms including antibacterial humoral response, humoral immune response, cell killing, and immunoglobulin-mediated antibacterial immunity (**Figure 9c**). GO Cellular Component enrichment showed enrichment in secretory granule lumens, tertiary granules, secretory vesicles, and azurophil granule lumens (**Figure 9d**). GO Molecular Function enrichment highlighted signaling receptor binding, protein phosphorylated amino acid binding, phosphatidylinositol binding, and phosphatase binding (**Figure 9e**). KEGG pathway enrichment identified MAS-related pathways including chemokine signaling, HIF-1 signaling, IL-17 signaling, AGE-RAGE signaling, and Toll-like receptor signaling (**Figure 9f**).

Together, these proteomic results corroborate our serological observations at the protein level and show that CRP exhibits extensive connectivity within inflammatory protein networks in autoinflammatory diseases, including MAS. These network-based observations are correlative and require functional validation.

#### 3.8.2 Comprehensive proteomic profiling of CRP’s global expression landscape and core molecular structural basis

Building on the disease-specific proteomic signatures of CRP in MAS, we next performed a pan-proteomic analysis to define the global expression landscape and core molecular structure of CRP across physiological contexts. CRP is a canonical secretory pattern recognition molecule whose tissue-specific expression, secretory characteristics, and three-dimensional conformation are key determinants of its physiological functions and pathological roles. Although serum CRP levels have long been used clinically as a marker of inflammation, systematic proteomic validation of CRP expression across normal tissues, body fluids, and cellular models has been limited. To address this gap, we integrated data from three proteomic databases, ProteomicsDB, PaxDb, and MOPED, to quantify CRP protein distribution across normal human tissues, body fluids, primary cells, and cell lines. We also defined the core molecular structure of CRP using the PubChem database, establishing a foundation for subsequent mechanistic studies.

**Figure 10:**
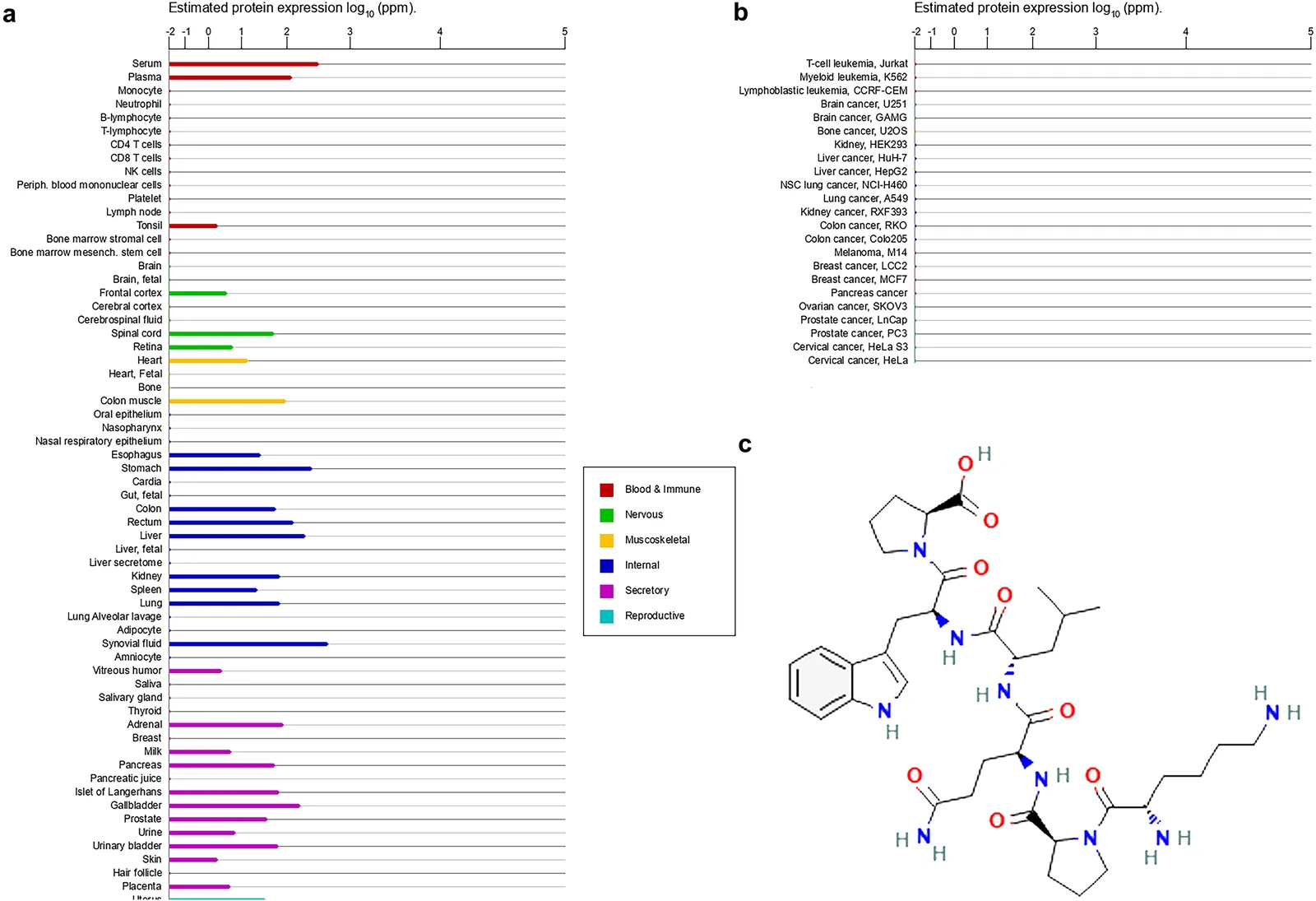
Global expression distribution characteristics and core molecular structure of CRP protein. **(a)** Quantitative expression profile of CRP protein in normal human body fluids, tissues and primary cells, integrated from ProteomicsDB, PaxDb and MOPED databases. The x-axis shows protein expression levels normalized to log_10_(ppm), and samples are grouped into six functional systems with color coding: blood and immune system (red), nervous system (green), musculoskeletal system (yellow), visceral system (blue), secretory gland system (purple) and reproductive system (cyan); **(b)** Quantitative expression profile of CRP protein in human cancer cell lines of different tissue origins, based on the integrated proteomic databases. The x-axis represents protein expression levels normalized to log_10_(ppm); **(c)** Core covalent molecular structure of the CRP monomer retrieved from the PubChem database, illustrating the atomic arrangement and spatial localization of functional groups corresponding to its amino acid sequence.

First, we constructed a global expression map of CRP protein in normal human body fluids, tissues, and primary cells based on integrated quantitative proteomic data (**Figure 10a**). Protein quantification was normalized to log_10_(ppm). The results showed a typical secretory protein pattern: CRP abundance was highest in serum and plasma, consistent with the physiological process by which liver-synthesized CRP enters the circulation through constitutive and inducible secretory pathways. At the cellular level, low levels of CRP protein were detected in circulating immune cells including peripheral blood monocytes, neutrophils, B lymphocytes, and T lymphocytes, while very low abundance was observed in peripheral immune organs such as lymph nodes and tonsils. Among parenchymal organs, the liver showed the highest CRP expression, with levels well above those in the gastrointestinal tract, kidney, spleen, lung, and other visceral organs. In contrast, only trace background signals or no detectable CRP expression were found in most tissues of the nervous system, musculoskeletal system, reproductive system, and endocrine or exocrine glands. These proteomic findings align closely with transcriptomic data and confirm at the protein level that the liver is the primary organ for CRP synthesis under physiological conditions, and that liver-derived CRP constitutes the major source of circulating CRP.

To further validate the cell lineage specificity of CRP expression in *in vitro* cellular models, we systematically analyzed CRP protein expression across human cancer cell lines derived from various tissue origins (**Figure 10b**). Consistent CRP expression was detected exclusively in liver-derived hepatocellular carcinoma cell lines HepG2 and Huh7. No significant CRP expression was observed in cancer cell lines of other lineages, including hematological malignancies (T-cell leukemia, myeloid leukemia, lymphoma), central nervous system tumors, lung cancer, renal cancer, colon cancer, melanoma, breast cancer, ovarian cancer, prostate cancer, or cervical cancer. These *in vitro* findings further indicate that CRP synthesis and expression are largely restricted to the hepatocyte lineage. Notably, even malignantly transformed hepatocytes retain the ability to produce CRP, providing a useful cellular model for subsequent functional and mechanistic studies.

Finally, we characterized the core molecular structure of the CRP monomer using the PubChem database (**Figure 10c**). In humans, functional CRP exists as a homopentamer, with each monomer composed of 206 amino acid residues folded into a characteristic lectin-like β-barrel conformation. The molecule contains several conserved functional domains, including a phosphocholine-binding site, a complement C1q-binding domain, and an Fcγ receptor-binding region. These structural motifs support CRP’s core biological functions, such as pathogen recognition, classical complement pathway activation, and immune cell opsonization. Elucidation of CRP’s molecular structure provides a structural basis for further investigation of its molecular interactions in liver diseases and systemic inflammatory disorders.

### 3.9 Cell-specific expression and spatial distribution of C-reactive protein in healthy human liver tissue: insights from single-cell and spatial transcriptomics

The liver is the main site of CRP synthesis, but the precise cellular source and spatial distribution of CRP in healthy human liver have not been systematically examined using integrated single-cell and spatial transcriptomic approaches. To address this, we analyzed paired single-cell CITE-seq and spatial transcriptomic data from healthy human liver (GSE192742) to profile CRP expression at both cellular and tissue levels.

After quality control, dimensionality reduction, clustering, and cell-type annotation, we identified 18 major liver cell populations, covering parenchymal and non-parenchymal lineages, including hepatocytes, macrophages, endothelial cells, T cells, B cells, natural killer cells, monocytes, neutrophils, cholangiocytes, fibroblasts, conventional dendritic cells, plasmacytoid dendritic cells, basophils, and plasma cells (**Supplementary Figure 1a**). This provided a single-cell reference atlas for subsequent expression analysis.

At the single-cell level, CRP transcripts were detected exclusively in hepatocytes, with no expression observed in any non-parenchymal cell type, including immune cells, stromal cells, endothelial cells, or biliary epithelial cells (**Supplementary Figure 1b**). These findings indicate that hepatocytes are the primary cellular source of CRP in healthy human liver.

To examine the spatial distribution, we analyzed the paired spatial transcriptomic data from the same specimens. Spot-level deconvolution and annotation reconstructed the native liver architecture, identifying hepatocyte-enriched parenchyma, portal regions with cholangiocytes, vascular areas with fibroblasts/endothelial cells, and the locations of resident Kupffer cells and immature lipid-associated macrophages (**Supplementary Figure 1c**). The spatial expression heatmap showed that CRP transcripts were concentrated in hepatocyte-rich areas and were nearly absent from non-hepatocyte regions such as portal tracts and bile duct zones (**Supplementary Figure 1d**). These spatial data further support hepatocyte-restricted CRP expression and define its confinement within the liver parenchyma, providing a basis for investigating local regulatory networks in systemic inflammation.

### 3.10 Multi-dimensional omics dissection of the CRP gene: database-based mining of functions, regulatory networks and disease targets

To complement our single-cell and spatial transcriptomic findings, we performed a bioinformatic analysis of the CRP gene using multi-dimensional public datasets from the Harmonizome database. This analysis was intended to explore the broader cellular functions, upstream transcriptional regulation, potential pharmacological targets, and disease associations of CRP.

Analysis of the Achilles dataset showed that CRP knockdown had cell type-specific effects on proliferation fitness. We identified 10 cell lines with increased fitness (most notably in hematological malignancies such as HL60, MOLM13, and OCI-AML2) and 12 with decreased fitness (including SKNO-1, GP2D, and HS683) (**Supplementary Figure 2a**). These context-dependent effects suggest that CRP may influence tumour growth in a cell type-specific manner.

To explore potential mechanisms underlying aberrant CRP expression, we analyzed the ChEA dataset and identified 74 transcription factors that bind to the CRP promoter or regulatory regions (**Supplementary Figure 2b**), including known regulators such as AR, CBX2, MYC, E2F1, ESRRA, STAT2, and RXRA. This regulatory network may help investigate pathways involved in dysregulated CRP production.

We also examined compounds and chemicals that modulate CRP expression or interact with CRP. The CMAP dataset identified 42 compounds that significantly alter CRP transcript levels (**Supplementary Figure 2c**), including budesonide, estradiol, progesterone, cefalexin, and doxycycline. The CTD dataset revealed 124 exogenous chemicals that interact with CRP (**Supplementary Figure 2d**), covering clinical drugs (atorvastatin, metformin), natural compounds (curcumin, resveratrol), and environmental agents.

Lastly, we assessed disease associations using the DISEASES curated scores, which showed high-confidence links between CRP and nine major disease categories (**Supplementary Figure 2e**). Consistent with our clinical observations, CRP was most enriched in inflammatory and autoimmune conditions, including connective tissue diseases, hypersensitivity, immune system disorders, lupus, and musculoskeletal diseases.

## 4 Discussion

AIDs are a heterogeneous group of disorders driven by dysregulated innate immunity, with a rising global prevalence. However, clinical management is often limited by the lack of validated disease-specific biomarkers for accurate diagnosis, disease activity monitoring, and risk stratification (26–28). CRP is the most widely used systemic inflammatory marker in clinical practice and has been applied to AIDs for decades. Nevertheless, previous studies have been constrained by single-disease designs, small sample sizes, heterogeneous cohorts (including post-treatment samples with therapy-induced biomarker alterations), and insufficient integration of multi-omics data to characterize CRP’s cellular origin, regulatory networks, and disease-specific functional properties.

In this study, we established a large single-center retrospective cohort covering 11 types of adult and pediatric AIDs (AOSD, MAS, BD, SpA, IBD, gout, RP, SAR, SS, SJIA, KD) and two age-matched healthy control groups. Strict inclusion of pre-treatment baseline samples helped minimize the confounding effects of anti-inflammatory or immunosuppressive therapy. By integrating clinical phenomics, serum proteomics, single-cell CITE-seq, and spatial transcriptomics, we performed a multi-dimensional characterization of CRP across the spectrum of AIDs. We identified a disease-specific hierarchical expression pattern of CRP and its correlation with core clinical manifestations in AOSD. We also showed that CRP composite indices (CLR and CAR) may offer advantages over CRP alone for risk stratification in AIDs, with stable predictive value across clinically relevant subgroups. Single-cell and spatial transcriptomic data identified hepatocytes as the primary source of CRP transcripts in human liver, and proteomic network analyses revealed that CRP is broadly engaged in interactions within the inflammatory protein network. Together, these observational and bioinformatic findings generate hypotheses regarding CRP biology and provide a basis for future investigations into its potential role in AID pathogenesis and management.

### 4.1 Disease-specific heterogeneity of CRP expression: biological basis and clinical diagnostic implications

A core result of this study is the inter-disease heterogeneity of serum CRP levels across AIDs, with a clear hierarchical distribution (**Figure 8**). The highest median CRP levels were detected in SS (69.00 mg/L), AOSD (55.75 mg/L), and MAS (37.95 mg/L). Moderate elevations were observed in gout (9.00 mg/L), RP (7.75 mg/L), and SJIA (6.95 mg/L). Mild to moderate increases were found in KD (5.30 mg/L), IBD (5.00 mg/L), BD (4.80 mg/L), SpA (4.60 mg/L), and SAR (4.30 mg/L). All comparisons were conducted in a standardized cohort with strict exclusion of patients who received anti-inflammatory or immunosuppressive therapy before sampling, which helped eliminate treatment induced biomarker suppression, a major confounder in prior studies. This design ensures that the observed CRP differences reflect the intrinsic pathophysiology of each AID rather than heterogeneous clinical interventions.

The hierarchical CRP distribution aligns with the core pathogenic mechanisms of each disease. SS, AOSD, and MAS, which have the highest CRP levels, are all fulminant IL-1 and IL-6 driven hyperinflammatory conditions (cytokine storm syndromes). As a classic acute phase reactant, hepatic CRP synthesis is directly and primarily induced by IL-6 through the JAK-STAT3 pathway (26). In AOSD, a feed forward inflammatory loop drives pathogenesis: activated macrophages and neutrophils secrete large amounts of IL-1β and IL-6, which stimulate hepatocytes to produce large quantities of CRP and other acute phase proteins; these proteins may further amplify inflammation through complement activation and Fcγ receptor mediated immune cell stimulation. This mechanism helps explain our finding that CRP levels were strongly and specifically correlated with AOSD’s core clinical manifestations (fever, evanescent rash, arthralgia), whereas in most other AIDs, CRP appeared to reflect only global systemic inflammatory burden. A weak association with abdominal pain was observed in IBD, but no significant correlations with disease specific phenotypes were found in the remaining conditions.

This observation may help refine CRP’s biomarker role in AIDs. In AOSD, CRP levels correlate closely with IL-6-driven systemic inflammation and show strong associations with core disease manifestations, supporting its utility as a marker of disease activity rather than a direct indicator of the underlying pathogenic process. In contrast, in BD, SpA, and SAR, which are characterized by compartmentalized tissue inflammation (mucosal ulcers, enthesitis, granulomas), elevated serum CRP may represent a spillover of local inflammatory mediators into the circulation rather than a direct reflection of disease specific pathogenesis. This offers a possible explanation for the long standing clinical observation that CRP correlates poorly with disease activity in localized AIDs and helps clarify the limited utility of CRP alone in these conditions.

From a clinical perspective, the disease specific CRP hierarchy provides a useful framework for the differential diagnosis of AIDs, particularly in patients with undifferentiated fever. For example, in a patient with unexplained fever and highly elevated CRP (above 50 mg/L), SS, AOSD, and MAS should be considered as priority diagnoses rather than milder inflammatory diseases such as SpA or SAR. Conversely, in a patient with mucosal ulcers and only mild CRP elevation, BD remains a plausible diagnosis even without significant systemic inflammation. This clinical hierarchy, when viewed alongside our single-cell and spatial transcriptomic data identifying hepatocytes as the sole CRP source, suggests that the observed differences across diseases are likely driven by varying systemic signals rather than by extrahepatic local production.

### 4.2 Incremental predictive value of CRP composite indices (CLR and CAR) for risk stratification

Despite being the most widely used inflammatory marker, CRP’s clinical utility is limited by its lack of specificity, as its levels can be influenced by age, sex, nutritional status, diabetes, and immune cell homeostasis (29). To address this, we evaluated the performance of two CRP composite indices, CLR and CAR. Our results showed associations of CLR and CAR with AID risk across the disease entities we examined, with dose-response relationships and preserved predictive value in subgroup analyses stratified by age, sex, and diabetes status.

The utility of these composite indices may come from their integration of complementary inflammatory dimensions, which helps reduce the confounding effects of non-specific CRP fluctuations. CLR combines CRP (a marker of innate immune activation) with lymphocyte count (a marker of adaptive immune homeostasis and inflammatory exhaustion). Persistent innate immune activation in AIDs often induces lymphocyte apoptosis and lymphopenia, so CLR reflects both pro-inflammatory drive and counter-regulatory immune responses, offering a broader inflammatory assessment than either marker alone. Similarly, CAR integrates CRP with serum albumin, a negative acute-phase reactant that also reflects nutritional status, hepatic synthetic function, and vascular permeability, all of which are frequently altered in AIDs. Normalizing CRP to albumin may help correct for non-specific effects of malnutrition, chronic liver disease, and comorbidities, potentially yielding a more stable and disease-specific biomarker.

Subgroup analyses showed effect modifications by sex, age, and diabetes status. For example, the association between elevated CRP and BD risk was more pronounced in female patients, possibly due to estrogen’s regulatory effect on hepatic CRP synthesis. In diabetic patients, chronic low-grade inflammation may elevate baseline CRP, which could reduce the diagnostic specificity of CRP alone for acute AID flares. In contrast, CLR and CAR maintained consistent predictive performance across these subgroups, as they inherently adjust for confounding effects of sex hormones, age-related immune senescence, and diabetes-associated metabolic inflammation.

These findings suggest that CLR and CAR could be considered for routine diagnostic and risk stratification workflows in AIDs, particularly in patients with comorbidities where CRP alone may be less reliable. Future prospective studies are needed to validate optimal cut-off values for these indices in specific AIDs and to assess their utility in monitoring treatment response and predicting long-term outcomes.

### 4.3 CRP connectivity in inflammatory networks of AIDs: proteomic observations

Historically, CRP has been regarded as a passive bystander marker of inflammation, with little focus on its direct functional role in AID pathogenesis (30). Our proteomic analysis showed that CRP was among the proteins with the highest degree of connectivity in the MAS network, which is the most severe hyperinflammatory complication of AIDs. Within this network, CRP was noted to interact with 42 proteins. Functional enrichment revealed a unique signature dominated by antibacterial humoral responses, cell killing, and immunoglobulin-mediated immunity, reflecting the fulminant cytokine storm and uncontrolled macrophage activation of MAS. Whether these interactions reflect any contribution to macrophage activation or cytokine amplification cannot be determined from the current data. CRP is one of the earliest and most markedly elevated markers in MAS, often preceding severe clinical manifestations, but this temporal association does not imply causation (31). These network-based observations are correlative and do not establish a specific functional role for CRP; functional studies are needed to clarify the nature of these associations.

Although anti-CRP antibodies have been tested in preclinical models, our proteomic observations are correlative and do not by themselves establish a rationale for therapeutic intervention in MAS. The translational relevance of these network findings remains unclear. Our pan-proteomic analysis further confirmed that CRP is predominantly expressed in the liver and systemic circulation, with only trace levels in extrahepatic tissues, consistent with our single-cell transcriptomic results. Malignant hepatocyte cell lines (HepG2, Huh7) retain robust CRP expression, while non-hepatic cancer cell lines do not, confirming the strict hepatocyte lineage restriction of CRP and providing a valid *in vitro* model for future functional studies.

Although our study focused on autoinflammatory diseases, comparing them to systemic lupus erythematosus (SLE), where type I interferon dominates, helps clarify disease-specific regulation of CRP. In SLE, despite significant systemic inflammation, CRP levels rise only modestly, mainly because type I interferon suppresses CRP transcription in the liver (26). In addition, pentameric CRP can convert to its monomeric form in peripheral tissues, and the monomeric form is more proinflammatory (26). These features explain why a normal CRP in SLE does not rule out active disease, while a clearly elevated CRP often signals infection or serositis. In our autoinflammatory cohort, hyperinflammatory conditions such as MAS are largely driven by interleukin-6 (IL-6), not type I interferon. Therefore, CRP remains a more direct and reliable marker of disease activity in these diseases. This distinction reinforces our main point: CRP interpretation must be disease-specific, and composite markers such as CLR and CAR may help overcome context-dependent limitations. Complementing this, our proteomic network analysis positions CRP as a highly connected node in MAS, indicating that in hyperinflammatory states, CRP does not act in isolation but is embedded within an interactive inflammatory protein network, a feature that may underlie its strong clinical correlations in such conditions.

### 4.4 Evidence of hepatocyte-exclusive CRP expression: single-cell and spatial transcriptomic insights

The cellular origin of circulating CRP has long been a subject of debate. While the liver is recognized as the primary site of CRP synthesis, several *in vitro* studies have reported CRP expression in extrahepatic cells (monocytes, macrophages, endothelial cells, smooth muscle cells), leading to the hypothesis that local CRP production may contribute to disease pathogenesis (30, 32–36). However, these studies were limited by artificial *in vitro* culture conditions and a lack of single-cell *in vivo* validation in human tissues.

Using integrated single-cell CITE-seq and spatial transcriptomics of healthy human liver tissue, we sought to address this controversy. At single-cell resolution, CRP transcripts were found to be highly expressed in hepatocytes, with no detectable expression in any non-parenchymal cells (immune cells, endothelial cells, fibroblasts, cholangiocytes). Spatial transcriptomics further showed that CRP-high regions overlapped with hepatocyte-enriched parenchymal areas, with no expression observed in portal tracts or bile duct zones. Our integrated single-cell and spatial transcriptomic data identify hepatocytes as the primary cellular source of CRP in healthy human liver, and thus the main contributor to circulating CRP.

This finding has several implications for CRP biology and AID pathogenesis. It suggests that local extrahepatic CRP production does not contribute substantially to circulating levels or to AID pathogenesis; rather, elevated serum CRP in AIDs appears to be driven primarily by hepatic synthesis induced by systemic inflammatory mediators, especially IL-6. It also points to hepatocytes as a cellular target for therapeutic modulation of CRP production, which may help minimize off-target effects. In addition, this finding helps explain the well documented clinical observation that patients with severe hepatic dysfunction often show blunted CRP elevation even in the setting of severe inflammation, a phenomenon that now has a cellular level mechanistic basis. Thus, the hepatocyte-centric origin of CRP provides a cellular anchor for both the disease-specific serum hierarchy observed clinically and the protein-level interactivity detected in proteomic datasets, linking the three levels of evidence.

Taken together, the clinical, cellular, and proteomic layers converge on a coherent picture: the disease-specific CRP levels reflect the intensity of systemic inflammatory signals acting on hepatocytes, the sole source of circulating CRP; once released, CRP is part of a broader inflammatory interactome, as observed in MAS. These correlations, while not establishing causality, provide a framework for interpreting CRP in AIDs and may help guide future mechanistic investigations.

### 4.5 Limitations of the study

This study has several limitations. First, it is a single-center retrospective cohort, which carries inherent selection bias. Our findings would benefit from validation in multi-center prospective international cohorts, especially given that our population is exclusively Chinese. Second, sample sizes for some rare AID subgroups (SS, n=15; KD, n=6) are small, which limits statistical power for detailed subgroup analyses. Future studies using international rare disease registries could help validate these preliminary findings. Third, we only analyzed baseline pre-treatment CRP levels. Longitudinal changes in CRP and its composite indices during disease progression and treatment, as well as their potential prognostic value for treatment response, relapse, and long-term outcomes, remain to be investigated. The limited PCT measurements in AOSD also precluded assessment of its incremental value over CRP. Fourth, diagnostic overlap existed between certain disease pairs in our cohort. For IBD and SpA, as well as for SJIA and MAS, only one overlapping case was identified in each pair, which precluded meaningful sensitivity analysis. For AOSD and MAS, among 31 AOSD patients, 21 (67.7%) also met MAS criteria. While this overlap reflects the well-recognized clinical entity of MAS secondary to active AOSD, it limits our ability to distinguish CRP-associated biomarker signatures unique to each condition. Due to the limited number of non-overlapping AOSD cases (n = 10), formal sensitivity analyses excluding overlapping patients were not statistically feasible. Fifth, our proteomic analysis relied on public datasets rather than paired serum samples from our clinical cohort. Future paired proteomic profiling may help identify disease-specific CRP protein-protein interaction networks across different AIDs. Finally, our data are observational and correlational in nature; they do not address causality or mechanisms. Thus, any mechanistic interpretations in this Discussion remain speculative. Further *in vitro* and *in vivo* studies are needed to explore this, particularly in the context of cytokine storm in MAS and AOSD. Notably, our findings are derived from a single-center retrospective cohort without external validation. Institutional biases and population-specific inflammatory patterns may limit generalizability. Therefore, our results should be interpreted as hypothesis-generating rather than conclusive for clinical practice.

## 5 Conclusions and future perspectives

This study presents a multi-dimensional characterization of C-reactive protein (CRP) across the full spectrum of adult and pediatric autoinflammatory diseases (AIDs). We have delineated disease-specific hierarchical expression patterns of CRP, demonstrated the risk stratification performance of the CRP/lymphocyte ratio (CLR) and CRP/albumin ratio (CAR), identified hepatocytes as the principal source of CRP in healthy human liver, and showed that CRP exhibited connectivity within the inflammatory interactome, although these are observational findings. While CRP remains a primary marker, CLR and CAR showed associations with disease risk in specific subgroups. These findings may inform our understanding of CRP biology and have potential clinical relevance for the diagnosis, risk stratification, and management of AIDs.

Building on these observations, the present study may serve as a reference for the translational application of CRP and its composite indices in the precision management of autoinflammatory diseases, with possible implications for both clinical practice and basic research. In clinical settings, we suggest that disease-specific CRP interpretation criteria be considered to replace the traditional unified threshold, and that clinical decision-support tools incorporating disease-specific CRP reference ranges be explored. Such tools may assist in the differential diagnosis of undifferentiated fever and the assessment of disease activity, particularly in distinguishing hyperinflammatory conditions such as adult-onset Still’s disease (AOSD) and macrophage activation syndrome (MAS) from milder autoinflammatory diseases including spondyloarthritis, sarcoidosis, and Behçet’s disease. Meanwhile, we propose that CLR and CAR be evaluated further as optimized inflammatory biomarkers in routine clinical practice, with disease-specific cutoffs to be explored for risk stratification, disease activity monitoring, and prognostic evaluation. Their value in predicting treatment response, relapse, and long-term outcomes in AIDs warrants further investigation. Given the potential effect modifications exerted by gender, age, diabetes, and other demographic and comorbidity factors, stratified assessment strategies integrating CRP, CLR, and CAR may be considered for targeted populations, such as female patients with Behçet’s disease, diabetic patients with sarcoidosis or relapsing polychondritis, and young or elderly patients with spondyloarthritis, to improve the accuracy of individualized patient management.

For basic and translational research, further efforts are needed to clarify the causal pathogenic role of CRP in autoinflammatory diseases, particularly its involvement in the cytokine storm associated with AOSD and MAS. Whether these network observations have any implications for anti-CRP targeting remains to be seen and is not addressed by this study. Multi-omics integrated research may help to dissect the molecular regulatory networks underlying disease-specific CRP expression. This could include single-cell transcriptomics of liver tissue from patients with active AIDs, genome-wide association studies, and Mendelian randomization analyses to elucidate the transcriptional regulation of CRP in hepatocytes and the genetic variants modulating circulating CRP levels. To assess the generalizability and robustness of our conclusions, the disease-specific CRP expression profiles and composite indices would benefit from validation in multi-center prospective international cohorts covering diverse ethnic populations, with expanded sample sizes for rare autoinflammatory diseases such as Sweet syndrome and Kawasaki disease. Longitudinal follow-up studies are also needed to examine the long-term predictive value of CLR and CAR for disease recurrence, progression, and treatment response. Ultimately, personalized biomarker panels integrating CRP, CLR, CAR, and other disease-specific molecules could be developed, and combined with targeted therapeutic markers such as those guiding interleukin-6 inhibitors or JAK inhibitors, to support the precision medicine of autoinflammatory diseases from inflammatory monitoring toward individualized treatment selection.

Besides, experimental validation will be essential to move beyond correlation. We therefore propose that future work includes RT-qPCR or Western blot analysis of CRP-associated genes in patient PBMCs, as well as THP-1 macrophage stimulation assays to assess whether the composite indices (CLR, CAR) reflect genuine changes in inflammatory pathway activity.

In summary, this study suggests that CRP may be reconsidered not merely as a non-specific inflammatory marker but as a disease-relevant biomarker in AIDs, and highlights the potential value of CLR and CAR as composite indices. Our findings may help address a gap in the systematic characterization of CRP in AIDs and offer a possible direction for future research and translational efforts in the precision medicine of innate immune-mediated inflammatory diseases. However, due to the lack of external validation, these suggestions remain preliminary and require confirmation in independent cohorts before translation into routine clinical use.

## Data Availability

The single-cell CITE-seq and spatial transcriptomic data analyzed in this study are publicly available in the Gene Expression Omnibus (GEO) database under accession number GSE192742. The macrophage activation syndrome (MAS)-specific proteomic dataset is available in the GEO database under accession number GSE197546. The clinical cohort data used in this study were collected, securely stored and standardizedly managed with technical support from the Yiduyun platform. These clinical data are not publicly available due to ethical restrictions and patient privacy regulations. De-identified clinical data can be obtained from the corresponding authors upon reasonable request, subject to approval by the Ethics Committee of Nanjing Drum Tower Hospital and signing of a data use agreement for non-commercial research purposes only. All other data supporting the findings of this study are available within the article and its **Supplementary Materials**.
