# Supplementary material for: Disease-specific biomarker value of C-reactive protein and its composite indices in autoinflammatory diseases: an integrated study of multi-modal omics and population cohorts
Source: Front Immunol. 2026 Aug 13;17:1874052. doi: 10.3389/fimmu.2026.1874052 (PMC13518195; doi:10.3389/fimmu.2026.1874052)
Supplement: Supplementary file 1 [file DataSheet1.docx]

**Supplementary figure 1 Cell-Specific Expression and Spatial Distribution of CRP in Healthy Human Liver Tissue Revealed by Single-Cell and Spatial Transcriptomics**

(a) UMAP dimensionality reduction and clustering plot based on single-cell CITE-seq data of healthy human liver tissue from the GSE192742 dataset, showing the clustering results of all cell lineages in liver tissue; (b) UMAP plot overlaid with the expression abundance of the CRP gene, where the color gradient represents the normalized expression level of CRP; (c) Spot-level cell type annotation map of spatial transcriptomic sequencing of healthy human liver tissue, showing the in situ spatial distribution of cell types in liver tissue. Scale bar: 50 μm; (d) Spatial expression heatmap of the CRP gene in the same healthy liver tissue section paired with panel c, where the color gradient represents the normalized expression level of CRP. Scale bar: 2 mm.

**Supplementary figure 2 Panoramic multi-dimensional biological characterization of the CRP gene based on Harmonizome database integration**

(a) Results from the Achilles Cell Line Gene Essentiality Profiles dataset showing cell lines with significantly altered proliferation fitness following CRP gene knockdown, with separate listings for associations with increased fitness (top) and decreased fitness (bottom); (b) Results from the ChEA Transcription Factor Targets dataset showing transcription factors validated to bind the CRP gene promoter via functional experiments or ChIP-seq; (c) Results from the CMAP Signatures of Differentially Expressed Genes for Small Molecules dataset showing small molecule compounds that significantly alter CRP gene expression levels; (d) Results from the curated CTD Gene-Chemical Interactions dataset showing experimentally verified chemicals that interact with the CRP gene or protein; (e) Results from the DISEASES Curated Gene-Disease Association Evidence Scores dataset showing disease categories with high-confidence associations with the CRP gene.
