# Supplementary material for: Disease-specific biomarker value of C-reactive protein and its composite indices in autoinflammatory diseases: an integrated study of multi-modal omics and population cohorts
Source: Front Immunol. 2026 Aug 13;17:1874052. doi: 10.3389/fimmu.2026.1874052 (PMC13518195; doi:10.3389/fimmu.2026.1874052)
Supplement: Supplementary file 2 [file Table1.docx]

**Table S1 Related baseline laboratory characteristics of AOSD and Non-AOSD control population**

| **Variables** | **Total (n = 812)** | **Non-AOSD Controls (n = 406)** | **AOSD Patients (n = 406)** | **Statistic** | **P** |  |
| --- | --- | --- | --- | --- | --- | --- |
| Ferritin, M (Q₁, Q₃) | 324.75 (131.07, 887.50) | 210.70 (103.80, 355.10) | 655.30 (203.70, 1403.50) | Z=-7.82 | <.001 |  |
| D-D, M (Q₁, Q₃) | 0.72 (0.26, 2.12) | 0.41 (0.22, 0.97) | 2.32 (0.91, 6.25) | Z=-11.30 | <.001 |  |
| Fbg, M (Q₁, Q₃) | 3.30 (2.60, 4.50) | 3.00 (2.50, 3.80) | 4.50 (2.75, 5.70) | Z=-7.50 | <.001 |  |
| WBC, M (Q₁, Q₃) | 7.50 (5.40, 11.55) | 6.00 (4.80, 7.60) | 10.80 (7.50, 15.20) | Z=-14.93 | <.001 |  |
| Neutrophil Count, M (Q₁, Q₃) | 5.20 (3.30, 9.10) | 3.70 (2.60, 5.20) | 8.50 (5.40, 12.90) | Z=-15.65 | <.001 |  |
| Lymphocyte Count, M (Q₁, Q₃) | 1.40 (1.00, 1.90) | 1.50 (1.10, 2.00) | 1.30 (0.90, 1.80) | Z=-2.97 | 0.003 |  |
| Eso count, M (Q₁, Q₃) | 0.07 (0.01, 0.14) | 0.09 (0.03, 0.15) | 0.03 (0.00, 0.10) | Z=-6.85 | <.001 |  |
| Mono count, M (Q₁, Q₃) | 0.50 (0.30, 0.60) | 0.40 (0.30, 0.50) | 0.50 (0.30, 0.70) | Z=-5.59 | <.001 |  |
| Hemoglobin, M (Q₁, Q₃) | 123.00 (110.00, 136.00) | 124.00 (113.00, 137.75) | 119.00 (107.50, 133.00) | Z=-2.88 | 0.004 |  |
| Platelet Count, M (Q₁, Q₃) | 231.00 (176.50, 296.00) | 217.50 (168.00, 268.75) | 247.00 (193.00, 325.00) | Z=-5.38 | <.001 |  |
| Complement C3, M (Q₁, Q₃) | 1.27 (1.08, 1.51) | 1.16 (1.01, 1.33) | 1.38 (1.15, 1.60) | Z=-6.69 | <.001 |  |
| Complement C4, M (Q₁, Q₃) | 0.27 (0.21, 0.33) | 0.24 (0.19, 0.30) | 0.29 (0.23, 0.34) | Z=-4.89 | <.001 |  |
| Immunoglobulin A, M (Q₁, Q₃) | 2.19 (1.69, 2.90) | 2.13 (1.65, 2.97) | 2.20 (1.72, 2.82) | Z=-0.12 | 0.901 |  |
| Immunoglobulin E, M (Q₁, Q₃) | 71.00 (18.00, 160.00) | 27.00 (10.00, 116.00) | 105.00 (41.00, 247.50) | Z=-7.35 | <.001 |  |
| Immunoglobulin G, M (Q₁, Q₃) | 11.80 (10.00, 14.20) | 11.40 (9.53, 13.55) | 12.00 (10.20, 14.50) | Z=-1.82 | 0.069 |  |
| Immunoglobulin M, M (Q₁, Q₃) | 1.17 (0.87, 1.61) | 1.02 (0.79, 1.43) | 1.29 (0.95, 1.75) | Z=-4.34 | <.001 |  |
| LDH, M (Q₁, Q₃) | 243.00 (185.00, 389.00) | 200.00 (170.00, 246.00) | 352.00 (239.25, 558.00) | Z=-14.55 | <.001 |  |
| CK, M (Q₁, Q₃) | 37.00 (22.00, 66.50) | 44.00 (29.00, 85.50) | 24.00 (20.00, 42.50) | Z=-6.97 | <.001 |  |
| ALT, M (Q₁, Q₃) | 23.90 (14.60, 46.90) | 19.70 (12.50, 34.00) | 31.50 (17.08, 61.07) | Z=-7.03 | <.001 |  |
| AST, M (Q₁, Q₃) | 23.80 (16.83, 41.00) | 19.35 (15.30, 29.00) | 31.75 (20.00, 55.62) | Z=-9.67 | <.001 |  |
| Creatinine, M (Q₁, Q₃) | 54.00 (45.00, 66.00) | 60.00 (50.00, 72.78) | 49.00 (41.00, 60.00) | Z=-9.17 | <.001 |  |
| Urea, M (Q₁, Q₃) | 4.90 (3.80, 6.40) | 5.20 (4.10, 6.70) | 4.40 (3.30, 5.60) | Z=-6.38 | <.001 |  |
| eGFR, M (Q₁, Q₃) | 128.10 (102.75, 153.80) | 114.70 (90.90, 139.60) | 149.60 (129.90, 181.80) | Z=-10.68 | <.001 |  |
| RF, M (Q₁, Q₃) | 10.55 (7.00, 15.00) | 10.60 (3.58, 15.40) | 10.55 (8.70, 14.35) | Z=-1.46 | 0.145 |  |
| ANA, n(%) |  |  |  | χ²=1.80 | 0.180 |  |
| Negative | 175 (71.72) | 111 (68.94) | 64 (77.11) |  |  |  |
| Positive | 69 (28.28) | 50 (31.06) | 19 (22.89) |  |  |  |
| Urine Occult Blood, n(%) |  |  |  | χ²=19.26 | <.001 |  |
| Negative | 493 (73.25) | 264 (71.93) | 229 (74.84) |  |  |  |
| Weak Positive | 57 (8.47) | 46 (12.53) | 11 (3.59) |  |  |  |
| Positive | 123 (18.28) | 57 (15.53) | 66 (21.57) |  |  |  |
| Urine Protein, n(%) |  |  |  | χ²=3.93 | 0.140 |  |
| Negative | 469 (69.48) | 255 (69.48) | 214 (69.48) |  |  |  |
| Weak Positive | 101 (14.96) | 62 (16.89) | 39 (12.66) |  |  |  |
| Positive | 105 (15.56) | 50 (13.62) | 55 (17.86) |  |  |  |

Abbreviations: D‑D, D‑dimer; Fbg, fibrinogen; WBC, white blood cell; LDH, lactate dehydrogenase; ALT, alanine transaminase; AST, aspartate transaminase; eGFR, estimated glomerular filtration rate; ANA, antinuclear antibody; M, median; Q1, first quartile; Q3, third quartile.

**Table S2 Related baseline laboratory characteristics of BD and Non-BD control population**

| **Variables** | **Total (n = 1058)** | **Non-BD Controls (n = 545)** | **BD patients (n = 513)** | **Statistic** | **P** |
| --- | --- | --- | --- | --- | --- |
| Complement C3, M (Q₁, Q₃) | 1.21 (1.04, 1.39) | 1.27 (1.14, 1.43) | 1.18 (1.01, 1.37) | Z=-3.88 | <.001 |
| Complement C4, M (Q₁, Q₃) | 0.25 (0.19, 0.31) | 0.27 (0.22, 0.32) | 0.24 (0.19, 0.30) | Z=-2.78 | 0.006 |
| Immunoglobulin A, M (Q₁, Q₃) | 2.34 (1.76, 3.11) | 2.37 (1.83, 3.15) | 2.34 (1.71, 3.08) | Z=-1.04 | 0.298 |
| Immunoglobulin E, M (Q₁, Q₃) | 56.00 (25.50, 144.50) | 32.00 (17.00, 70.00) | 76.50 (34.25, 169.00) | Z=-6.56 | <.001 |
| Immunoglobulin G, M (Q₁, Q₃) | 11.90 (9.95, 14.45) | 12.30 (10.40, 14.20) | 11.70 (9.70, 14.50) | Z=-1.74 | 0.082 |
| Immunoglobulin M, M (Q₁, Q₃) | 1.15 (0.84, 1.58) | 1.09 (0.86, 1.48) | 1.18 (0.83, 1.62) | Z=-1.51 | 0.131 |
| WBC, M (Q₁, Q₃) | 6.60 (5.20, 8.90) | 6.50 (5.30, 8.70) | 6.70 (5.05, 8.95) | Z=-0.34 | 0.732 |
| Neutrophil Count, M (Q₁, Q₃) | 4.10 (3.00, 6.20) | 3.90 (2.90, 5.55) | 4.20 (3.10, 6.40) | Z=-1.81 | 0.071 |
| Lymphocyte Count, M (Q₁, Q₃) | 1.60 (1.20, 2.20) | 1.90 (1.40, 2.30) | 1.50 (1.00, 2.00) | Z=-6.80 | <.001 |
| Hemoglobin, M (Q₁, Q₃) | 129.00 (116.00, 142.00) | 135.00 (121.00, 145.00) | 126.00 (112.00, 139.50) | Z=-5.28 | <.001 |
| Platelet Count, M (Q₁, Q₃) | 228.50 (185.25, 282.00) | 252.00 (201.50, 299.50) | 217.00 (175.00, 274.00) | Z=-4.75 | <.001 |
| Albumin, M (Q₁, Q₃) | 40.30 (36.20, 43.10) | 43.30 (40.60, 45.40) | 38.35 (34.80, 41.50) | Z=-14.09 | <.001 |
| ALT, M (Q₁, Q₃) | 20.40 (12.95, 37.00) | 17.00 (12.30, 27.00) | 22.90 (13.40, 42.62) | Z=-4.72 | <.001 |
| AST, M (Q₁, Q₃) | 21.00 (15.43, 30.78) | 21.00 (15.83, 26.00) | 21.35 (15.20, 36.52) | Z=-2.14 | 0.032 |
| Total Bilirubin, M (Q₁, Q₃) | 9.10 (6.60, 13.10) | 10.30 (7.60, 13.60) | 8.50 (6.30, 12.30) | Z=-3.97 | <.001 |
| Uric Acid, M (Q₁, Q₃) | 299.00 (236.25, 365.75) | 297.50 (243.00, 352.75) | 299.00 (234.00, 367.00) | Z=-0.22 | 0.826 |
| Creatinine, M (Q₁, Q₃) | 56.00 (46.00, 69.00) | 58.60 (49.15, 68.70) | 54.00 (45.00, 69.00) | Z=-2.77 | 0.006 |
| Urea, M (Q₁, Q₃) | 4.70 (3.70, 5.70) | 4.60 (3.80, 5.60) | 4.70 (3.70, 5.90) | Z=-0.53 | 0.595 |
| eGFR, M (Q₁, Q₃) | 130.65 (108.85, 154.90) | 129.30 (114.07, 144.95) | 131.95 (107.95, 158.18) | Z=-0.77 | 0.442 |
| Urine Occult Blood, n(%) |  |  |  | χ²=2.00 | 0.157 |
| Negative | 479 (73.47) | 115 (69.28) | 364 (74.90) |  |  |
| Positive | 173 (26.53) | 51 (30.72) | 122 (25.10) |  |  |
| Urine Protein, n(%) |  |  |  | - | 0.358 |
| Negative | 461 (70.71) | 124 (74.70) | 337 (69.34) |  |  |
| Weak Positive | 3 (0.46) | 0 (0.00) | 3 (0.62) |  |  |
| Positive | 188 (28.83) | 42 (25.30) | 146 (30.04) |  |  |

Abbreviations: WBC, white blood cell; ALT, alanine transaminase; AST, aspartate transaminase; eGFR, estimated glomerular filtration rate; M, median; Q1, first quartile; Q3, third quartile.

**Table S3 Related baseline laboratory characteristics of SpA and Non-SpA control population**

| **Variables** | **Total (n = 665)** | **Non-SpA Controls (n = 288)** | **SpA Patients (n = 377)** | **Statistic** | **P** |
| --- | --- | --- | --- | --- | --- |
| Complement C3, M (Q₁, Q₃) | 1.25 (1.08, 1.54) | 1.12 (0.87, 1.36) | 1.34 (1.17, 1.65) | Z=-2.97 | 0.003 |
| Complement C4, M (Q₁, Q₃) | 0.25 (0.18, 0.32) | 0.24 (0.18, 0.28) | 0.27 (0.19, 0.35) | Z=-1.29 | 0.196 |
| Immunoglobulin A, M (Q₁, Q₃) | 2.37 (1.82, 3.50) | 2.32 (1.68, 3.53) | 2.44 (1.91, 3.43) | Z=-0.28 | 0.779 |
| Immunoglobulin E, M (Q₁, Q₃) | 43.00 (17.00, 98.00) | 45.00 (17.00, 106.00) | 41.50 (17.75, 95.00) | Z=-0.38 | 0.704 |
| Immunoglobulin G, M (Q₁, Q₃) | 11.70 (10.20, 13.80) | 11.30 (10.10, 12.70) | 12.25 (10.40, 14.05) | Z=-1.11 | 0.267 |
| Immunoglobulin M, M (Q₁, Q₃) | 1.21 (0.82, 1.61) | 0.99 (0.74, 1.51) | 1.25 (0.91, 1.65) | Z=-1.33 | 0.182 |
| WBC, M (Q₁, Q₃) | 6.60 (5.50, 8.00) | 6.30 (5.30, 7.75) | 6.90 (5.80, 8.10) | Z=-2.68 | 0.007 |
| Neutrophil Count, M (Q₁, Q₃) | 4.00 (3.10, 5.30) | 3.70 (3.00, 4.80) | 4.30 (3.40, 5.40) | Z=-3.90 | <.001 |
| Lymphocyte Count, M (Q₁, Q₃) | 1.90 (1.50, 2.40) | 1.90 (1.50, 2.40) | 1.90 (1.60, 2.40) | Z=-0.45 | 0.650 |
| Hemoglobin, M (Q₁, Q₃) | 140.00 (127.00, 152.25) | 140.00 (128.00, 151.00) | 140.00 (127.00, 154.00) | Z=-0.04 | 0.971 |
| Platelet Count, M (Q₁, Q₃) | 245.00 (198.00, 288.00) | 228.00 (180.50, 266.00) | 262.00 (218.00, 310.00) | Z=-6.68 | <.001 |
| Albumin, M (Q₁, Q₃) | 43.00 (40.90, 45.20) | 41.75 (39.80, 43.60) | 44.45 (42.20, 46.20) | Z=-9.44 | <.001 |
| ALT, M (Q₁, Q₃) | 18.90 (12.70, 29.00) | 18.35 (12.50, 31.92) | 19.00 (13.00, 28.00) | Z=-0.12 | 0.906 |
| AST, M (Q₁, Q₃) | 21.00 (16.10, 27.10) | 18.95 (14.97, 24.02) | 24.00 (18.45, 29.98) | Z=-6.17 | <.001 |
| Total Bilirubin, M (Q₁, Q₃) | 9.90 (7.40, 13.80) | 9.80 (7.30, 13.40) | 10.00 (7.60, 14.38) | Z=-0.61 | 0.542 |
| Uric Acid, M (Q₁, Q₃) | 323.50 (258.00, 400.75) | 336.50 (264.75, 421.00) | 314.00 (247.25, 381.75) | Z=-2.56 | 0.011 |
| Creatinine, M (Q₁, Q₃) | 60.20 (50.63, 73.12) | 59.00 (50.00, 74.00) | 60.75 (51.75, 72.00) | Z=-0.17 | 0.862 |
| Urea, M (Q₁, Q₃) | 5.00 (4.10, 6.10) | 5.00 (4.10, 6.23) | 4.90 (4.30, 6.00) | Z=-0.69 | 0.491 |
| eGFR, M (Q₁, Q₃) | 126.30 (109.70, 147.50) | 123.15 (108.07, 142.65) | 135.50 (117.40, 152.80) | Z=-3.75 | <.001 |
| Urine Occult Blood, n(%) |  |  |  | χ²=3.15 | 0.207 |
| Negative | 243 (71.05) | 189 (70.00) | 54 (75.00) |  |  |
| Weak Positive | 38 (11.11) | 28 (10.37) | 10 (13.89) |  |  |
| Positive | 61 (17.84) | 53 (19.63) | 8 (11.11) |  |  |
| Urine Protein, n(%) |  |  |  | χ²=1.75 | 0.416 |
| Negative | 268 (78.36) | 211 (78.15) | 57 (79.17) |  |  |
| Weak Positive | 38 (11.11) | 28 (10.37) | 10 (13.89) |  |  |
| Positive | 36 (10.53) | 31 (11.48) | 5 (6.94) |  |  |

Abbreviations: WBC, white blood cell; ALT, alanine transaminase; AST, aspartate transaminase; eGFR, estimated glomerular filtration rate; M, median; Q1, first quartile; Q3, third quartile.

**Table S4 Related baseline laboratory characteristics of IBD and Non-IBD control population**

| **Variables** | **Total (n = 732)** | **Non-IBD Controls (n = 359)** | **IBD Patients (n = 373)** | **Statistic** | **P** |
| --- | --- | --- | --- | --- | --- |
| Fecal Occult Blood, n(%) |  |  |  | χ²=14.93 | <.001 |
| No | 179 (49.72) | 88 (62.41) | 91 (41.55) |  |  |
| Yes | 181 (50.28) | 53 (37.59) | 128 (58.45) |  |  |
| Complement C3, M (Q₁, Q₃) | 1.18 (1.00, 1.34) | 1.07 (0.90, 1.26) | 1.21 (1.05, 1.37) | Z=-3.13 | 0.002 |
| Complement C4, M (Q₁, Q₃) | 0.25 (0.19, 0.31) | 0.23 (0.18, 0.30) | 0.25 (0.20, 0.31) | Z=-2.17 | 0.030 |
| Immunoglobulin A, M (Q₁, Q₃) | 2.13 (1.66, 2.82) | 2.11 (1.69, 2.70) | 2.16 (1.63, 2.91) | Z=-0.16 | 0.870 |
| Immunoglobulin E, M (Q₁, Q₃) | 30.00 (14.00, 81.25) | 41.50 (14.50, 126.75) | 27.00 (14.00, 69.75) | Z=-1.52 | 0.129 |
| Immunoglobulin G, M (Q₁, Q₃) | 11.40 (9.50, 13.40) | 11.45 (9.65, 13.00) | 11.40 (9.50, 13.57) | Z=-0.04 | 0.965 |
| Immunoglobulin M, M (Q₁, Q₃) | 0.95 (0.71, 1.24) | 1.00 (0.77, 1.24) | 0.94 (0.69, 1.24) | Z=-0.73 | 0.466 |
| WBC, M (Q₁, Q₃) | 5.90 (4.70, 7.50) | 5.70 (4.50, 7.40) | 6.10 (4.80, 7.75) | Z=-2.32 | 0.020 |
| Neutrophil Count, M (Q₁, Q₃) | 3.60 (2.60, 5.00) | 3.40 (2.40, 4.80) | 3.70 (2.90, 5.30) | Z=-3.41 | <.001 |
| Lymphocyte Count, M (Q₁, Q₃) | 1.50 (1.20, 2.10) | 1.60 (1.20, 2.10) | 1.50 (1.10, 2.00) | Z=-1.07 | 0.284 |
| Hemoglobin, M (Q₁, Q₃) | 129.00 (113.00, 143.00) | 129.00 (116.00, 143.00) | 128.00 (110.50, 144.50) | Z=-0.50 | 0.617 |
| Platelet Count, M (Q₁, Q₃) | 221.50 (178.25, 278.00) | 234.00 (187.00, 304.50) | 208.00 (166.00, 254.00) | Z=-4.52 | <.001 |
| Albumin, M (Q₁, Q₃) | 39.80 (36.70, 41.90) | 39.60 (36.90, 41.50) | 39.80 (36.40, 42.30) | Z=-0.35 | 0.726 |
| ALT, M (Q₁, Q₃) | 17.30 (11.00, 28.60) | 19.10 (12.10, 29.20) | 15.00 (10.25, 27.30) | Z=-2.94 | 0.003 |
| AST, M (Q₁, Q₃) | 18.30 (14.60, 25.30) | 19.60 (15.10, 26.30) | 17.50 (13.85, 23.05) | Z=-3.27 | 0.001 |
| Total Bilirubin, M (Q₁, Q₃) | 10.25 (7.10, 14.70) | 11.00 (8.00, 15.60) | 9.10 (6.75, 13.20) | Z=-3.99 | <.001 |
| Uric Acid, M (Q₁, Q₃) | 310.00 (249.00, 376.50) | 318.00 (263.50, 380.50) | 301.00 (238.00, 370.50) | Z=-2.85 | 0.004 |
| Creatinine, M (Q₁, Q₃) | 60.60 (50.95, 70.00) | 61.00 (50.20, 70.70) | 60.00 (51.00, 70.00) | Z=-0.17 | 0.866 |
| Urea, M (Q₁, Q₃) | 4.50 (3.50, 5.60) | 4.80 (3.80, 5.90) | 4.20 (3.20, 5.20) | Z=-4.79 | <.001 |
| eGFR, M (Q₁, Q₃) | 126.80 (108.38, 147.62) | 121.90 (105.10, 143.75) | 131.80 (112.30, 151.00) | Z=-3.58 | <.001 |
| Urine Occult Blood, n(%) |  |  |  | χ²=6.28 | 0.043 |
| Negative | 436 (84.01) | 237 (80.61) | 199 (88.44) |  |  |
| Weak Positive | 43 (8.29) | 28 (9.52) | 15 (6.67) |  |  |
| Positive | 40 (7.71) | 29 (9.86) | 11 (4.89) |  |  |
| Urine Protein, n(%) |  |  |  | χ²=6.00 | 0.050 |
| Negative | 364 (70.27) | 215 (73.13) | 149 (66.52) |  |  |
| Weak Positive | 113 (21.81) | 53 (18.03) | 60 (26.79) |  |  |
| Positive | 41 (7.92) | 26 (8.84) | 15 (6.70) |  |  |

Abbreviations: WBC, white blood cell; ALT, alanine transaminase; AST, aspartate transaminase; eGFR, estimated glomerular filtration rate; M, median; Q1, first quartile; Q3, third quartile.

**Table S5 Related baseline laboratory characteristics of SAR and Non-SAR control population**

| **Variables** | **Total (n = 582)** | **Non-SAR Controls (n = 328)** | **SAR Patients (n = 254)** | **Statistic** | **P** |
| --- | --- | --- | --- | --- | --- |
| Complement C3, M (Q₁, Q₃) | 1.22 (1.06, 1.36) | 1.13 (0.96, 1.26) | 1.25 (1.19, 1.46) | Z=-4.21 | <.001 |
| Complement C4, M (Q₁, Q₃) | 0.25 (0.21, 0.31) | 0.25 (0.19, 0.30) | 0.26 (0.22, 0.32) | Z=-1.86 | 0.064 |
| Immunoglobulin A, M (Q₁, Q₃) | 2.30 (1.69, 2.86) | 2.07 (1.53, 2.87) | 2.32 (1.90, 2.85) | Z=-1.00 | 0.319 |
| Immunoglobulin E, M (Q₁, Q₃) | 41.00 (18.00, 127.00) | 28.00 (12.00, 124.00) | 56.50 (26.00, 130.00) | Z=-1.97 | 0.049 |
| Immunoglobulin G, M (Q₁, Q₃) | 11.70 (10.20, 13.50) | 11.50 (10.20, 12.80) | 12.30 (10.20, 13.93) | Z=-1.11 | 0.269 |
| Immunoglobulin M, M (Q₁, Q₃) | 0.95 (0.74, 1.24) | 0.94 (0.74, 1.33) | 0.97 (0.74, 1.21) | Z=-0.08 | 0.939 |
| WBC, M (Q₁, Q₃) | 5.80 (4.80, 7.60) | 5.70 (4.80, 7.10) | 6.30 (4.80, 7.90) | Z=-2.06 | 0.040 |
| Neutrophil Count, M (Q₁, Q₃) | 3.50 (2.70, 4.77) | 3.40 (2.70, 4.50) | 3.80 (2.80, 5.18) | Z=-2.29 | 0.022 |
| Lymphocyte Count, M (Q₁, Q₃) | 1.60 (1.20, 2.10) | 1.60 (1.20, 2.10) | 1.50 (1.12, 1.98) | Z=-1.53 | 0.125 |
| Hemoglobin, M (Q₁, Q₃) | 131.50 (120.00, 145.00) | 131.00 (119.00, 145.00) | 132.00 (120.00, 144.00) | Z=-0.03 | 0.979 |
| Platelet Count, M (Q₁, Q₃) | 198.00 (160.00, 242.00) | 187.00 (153.75, 233.25) | 208.50 (171.00, 256.75) | Z=-3.82 | <.001 |
| Albumin, M (Q₁, Q₃) | 40.40 (38.10, 42.10) | 40.20 (37.90, 41.90) | 40.70 (38.40, 42.70) | Z=-2.14 | 0.033 |
| ALT, M (Q₁, Q₃) | 17.80 (12.80, 27.60) | 17.00 (12.30, 29.72) | 18.20 (13.10, 27.00) | Z=-0.19 | 0.848 |
| AST, M (Q₁, Q₃) | 19.60 (16.22, 25.80) | 19.30 (16.00, 26.05) | 19.65 (16.92, 25.40) | Z=-0.75 | 0.451 |
| Total Bilirubin, M (Q₁, Q₃) | 10.60 (7.80, 13.90) | 10.40 (7.57, 13.80) | 10.80 (8.00, 14.00) | Z=-0.98 | 0.327 |
| Uric Acid, M (Q₁, Q₃) | 339.50 (274.25, 409.75) | 338.00 (277.50, 414.25) | 343.50 (271.25, 408.75) | Z=-0.47 | 0.635 |
| Creatinine, M (Q₁, Q₃) | 64.00 (52.00, 78.00) | 64.75 (52.00, 78.00) | 63.00 (52.00, 77.00) | Z=-0.59 | 0.558 |
| Urea, M (Q₁, Q₃) | 5.50 (4.60, 6.90) | 5.60 (4.70, 7.10) | 5.35 (4.50, 6.50) | Z=-2.36 | 0.019 |
| eGFR, M (Q₁, Q₃) | 108.85 (90.23, 128.38) | 108.30 (90.68, 128.33) | 109.05 (90.12, 128.30) | Z=-0.26 | 0.798 |
| Urine Occult Blood, n(%) |  |  |  | χ²=9.52 | 0.009 |
| Negative | 360 (70.18) | 225 (72.35) | 135 (66.83) |  |  |
| Weak Positive | 67 (13.06) | 46 (14.79) | 21 (10.40) |  |  |
| Positive | 86 (16.76) | 40 (12.86) | 46 (22.77) |  |  |
| Urine Protein, n(%) |  |  |  | χ²=1.23 | 0.542 |
| Negative | 380 (74.07) | 231 (74.28) | 149 (73.76) |  |  |
| Weak Positive | 68 (13.26) | 44 (14.15) | 24 (11.88) |  |  |
| Positive | 65 (12.67) | 36 (11.58) | 29 (14.36) |  |  |

Abbreviations: WBC, white blood cell; ALT, alanine transaminase; AST, aspartate transaminase; eGFR, estimated glomerular filtration rate; M, median; Q1, first quartile; Q3, third quartile.

**Table S6 Related baseline laboratory characteristics of RP and Non-RP control population**

| **Variables** | **Total (n = 247)** | **Non-RP Controls (n = 153)** | **RP Patients (n = 94)** | **Statistic** | **P** |
| --- | --- | --- | --- | --- | --- |
| Complement C3, M (Q₁, Q₃) | 1.21 (0.99, 1.34) | 1.11 (0.89, 1.27) | 1.25 (1.09, 1.40) | Z=-2.59 | 0.010 |
| Complement C4, M (Q₁, Q₃) | 0.25 (0.20, 0.33) | 0.22 (0.15, 0.29) | 0.27 (0.22, 0.34) | Z=-2.64 | 0.008 |
| Immunoglobulin A, M (Q₁, Q₃) | 2.37 (1.63, 3.23) | 2.73 (1.62, 3.90) | 2.31 (1.65, 3.03) | Z=-1.11 | 0.268 |
| Immunoglobulin E, M (Q₁, Q₃) | 61.00 (22.00, 156.00) | 37.00 (8.00, 169.00) | 65.50 (34.50, 143.50) | Z=-1.10 | 0.271 |
| Immunoglobulin G, M (Q₁, Q₃) | 11.25 (9.07, 14.40) | 11.20 (10.20, 14.70) | 11.50 (8.70, 14.30) | Z=-0.59 | 0.555 |
| Immunoglobulin M, M (Q₁, Q₃) | 0.90 (0.67, 1.41) | 0.89 (0.71, 1.26) | 0.95 (0.66, 1.52) | Z=-0.60 | 0.550 |
| WBC, M (Q₁, Q₃) | 6.60 (5.20, 8.90) | 5.90 (4.90, 7.40) | 8.85 (6.27, 10.85) | Z=-6.41 | <.001 |
| Neutrophil Count, M (Q₁, Q₃) | 4.20 (2.90, 6.40) | 3.40 (2.60, 4.60) | 6.30 (3.98, 8.55) | Z=-6.64 | <.001 |
| Lymphocyte Count, M (Q₁, Q₃) | 1.60 (1.10, 2.20) | 1.60 (1.10, 2.08) | 1.70 (1.20, 2.40) | Z=-1.50 | 0.135 |
| Hemoglobin, M (Q₁, Q₃) | 130.00 (115.00, 144.00) | 128.00 (118.00, 144.00) | 131.00 (108.50, 141.50) | Z=-0.66 | 0.512 |
| Platelet Count, M (Q₁, Q₃) | 220.00 (167.25, 272.50) | 192.00 (156.00, 237.75) | 258.50 (212.25, 324.00) | Z=-6.22 | <.001 |
| Albumin, M (Q₁, Q₃) | 39.45 (36.82, 41.98) | 39.40 (37.30, 41.30) | 39.50 (35.10, 42.70) | Z=-0.77 | 0.441 |
| ALT, M (Q₁, Q₃) | 18.50 (12.90, 30.80) | 18.50 (12.50, 31.20) | 18.50 (14.20, 29.70) | Z=-0.66 | 0.510 |
| AST, M (Q₁, Q₃) | 19.55 (15.80, 25.25) | 20.20 (16.30, 26.50) | 17.70 (14.30, 23.15) | Z=-2.59 | 0.010 |
| Total Bilirubin, M (Q₁, Q₃) | 10.10 (6.65, 14.85) | 10.50 (6.80, 16.30) | 9.70 (6.60, 12.10) | Z=-2.36 | 0.018 |
| Uric Acid, M (Q₁, Q₃) | 324.00 (259.00, 407.00) | 354.00 (292.00, 430.00) | 272.00 (221.75, 349.00) | Z=-5.39 | <.001 |
| Creatinine, M (Q₁, Q₃) | 62.00 (49.75, 74.85) | 66.00 (55.00, 80.00) | 55.60 (45.00, 64.00) | Z=-4.75 | <.001 |
| Urea, M (Q₁, Q₃) | 5.90 (4.70, 7.50) | 6.20 (5.00, 8.30) | 5.20 (4.00, 6.40) | Z=-3.94 | <.001 |
| eGFR, M (Q₁, Q₃) | 112.65 (91.37, 134.78) | 105.40 (81.20, 128.90) | 129.20 (115.40, 155.20) | Z=-4.86 | <.001 |
| Urine Occult Blood, n(%) |  |  |  | χ²=3.01 | 0.222 |
| Negative | 158 (75.24) | 108 (75.52) | 50 (74.63) |  |  |
| Weak Positive | 14 (6.67) | 12 (8.39) | 2 (2.99) |  |  |
| Positive | 38 (18.10) | 23 (16.08) | 15 (22.39) |  |  |
| Urine Protein, n(%) |  |  |  | χ²=3.17 | 0.205 |
| Negative | 143 (68.10) | 95 (66.43) | 48 (71.64) |  |  |
| Weak Positive | 35 (16.67) | 22 (15.38) | 13 (19.40) |  |  |
| Positive | 32 (15.24) | 26 (18.18) | 6 (8.96) |  |  |

Abbreviations: WBC, white blood cell; ALT, alanine transaminase; AST, aspartate transaminase; eGFR, estimated glomerular filtration rate; M, median; Q1, first quartile; Q3, third quartile.

**Table S7 Related baseline laboratory characteristics of gout and Non-gout control population**

| **Variables** | **Total (n = 654)** | **Non-gout Controls (n = 327)** | **Gout Patients (n = 327)** | **Statistic** | **P** |
| --- | --- | --- | --- | --- | --- |
| Serum urine acid, M (Q₁, Q₃) | 389.00 (300.00, 493.00) | 347.00 (277.00, 426.00) | 479.00 (371.00, 572.50) | Z=-9.41 | <.001 |
| WBC, M (Q₁, Q₃) | 6.60 (5.30, 8.50) | 6.20 (5.00, 8.00) | 7.60 (6.27, 9.70) | Z=-5.97 | <.001 |
| Neutrophil Count, M (Q₁, Q₃) | 4.10 (3.00, 5.90) | 3.70 (2.80, 5.10) | 4.95 (3.70, 7.12) | Z=-6.08 | <.001 |
| Lymphocyte Count, M (Q₁, Q₃) | 1.70 (1.20, 2.30) | 1.60 (1.20, 2.20) | 1.70 (1.30, 2.30) | Z=-1.63 | 0.104 |
| Hemoglobin, M (Q₁, Q₃) | 137.00 (120.00, 150.00) | 135.00 (118.50, 147.00) | 143.00 (123.75, 155.25) | Z=-3.09 | 0.002 |
| Platelet Count, M (Q₁, Q₃) | 223.00 (175.50, 276.50) | 216.00 (172.50, 271.00) | 235.00 (188.50, 284.75) | Z=-2.36 | 0.018 |
| TG, M (Q₁, Q₃) | 1.29 (0.87, 1.92) | 1.21 (0.82, 1.76) | 1.66 (1.15, 2.61) | Z=-5.13 | <.001 |
| TC, M (Q₁, Q₃) | 4.30 (3.67, 5.00) | 4.20 (3.60, 4.93) | 4.67 (3.93, 5.41) | Z=-3.73 | <.001 |
| LDL, M (Q₁, Q₃) | 2.49 (1.93, 3.04) | 2.42 (1.89, 2.93) | 2.77 (2.20, 3.35) | Z=-3.66 | <.001 |
| HDL, M (Q₁, Q₃) | 1.03 (0.85, 1.26) | 1.03 (0.85, 1.27) | 1.02 (0.85, 1.24) | Z=-0.66 | 0.509 |
| Albumin, M (Q₁, Q₃) | 40.60 (37.12, 43.20) | 39.50 (36.30, 42.00) | 42.90 (40.00, 45.60) | Z=-8.10 | <.001 |
| ALT, M (Q₁, Q₃) | 22.50 (14.60, 39.30) | 20.30 (13.70, 34.50) | 28.70 (17.15, 46.35) | Z=-3.83 | <.001 |
| AST, M (Q₁, Q₃) | 21.00 (15.90, 27.90) | 18.90 (15.10, 25.60) | 24.00 (19.90, 32.88) | Z=-5.64 | <.001 |
| Total Bilirubin, M (Q₁, Q₃) | 9.75 (6.80, 13.38) | 8.60 (6.40, 12.10) | 11.90 (8.50, 14.90) | Z=-5.54 | <.001 |
| Creatinine, M (Q₁, Q₃) | 71.85 (59.00, 86.00) | 64.00 (53.75, 75.00) | 85.00 (75.00, 103.00) | Z=-10.69 | <.001 |
| Urea, M (Q₁, Q₃) | 5.40 (4.40, 6.90) | 5.40 (4.40, 6.80) | 5.40 (4.40, 7.30) | Z=-0.68 | 0.500 |
| eGFR, M (Q₁, Q₃) | 112.10 (88.20, 135.62) | 120.00 (97.65, 147.25) | 93.20 (70.20, 112.05) | Z=-7.34 | <.001 |
| Urine Occult Blood, n(%) |  |  |  | χ²=4.19 | 0.123 |
| Negative | 309 (80.05) | 255 (81.99) | 54 (72.00) |  |  |
| Weak Positive | 22 (5.70) | 15 (4.82) | 7 (9.33) |  |  |
| Positive | 55 (14.25) | 41 (13.18) | 14 (18.67) |  |  |
| Urine Protein, n(%) |  |  |  | χ²=3.26 | 0.196 |
| Negative | 289 (74.87) | 238 (76.53) | 51 (68.00) |  |  |
| Weak Positive | 48 (12.44) | 38 (12.22) | 10 (13.33) |  |  |
| Positive | 49 (12.69) | 35 (11.25) | 14 (18.67) |  |  |

Abbreviations: WBC, white blood cell; ALT, alanine transaminase; AST, aspartate transaminase; eGFR, estimated glomerular filtration rate; TG, triglyceride; TC, total cholesterol; LDL, low-density lipoprotein; HDL, high-density lipoprotein; M, median; Q1, first quartile; Q3, third quartile.
